# Protonated Forms of Naringenin and Naringenin Chalcone: Proteiform Bioactive Species Elucidated by IRMPD Spectroscopy, IMS, CID-MS, and Computational Approaches

**DOI:** 10.1021/acs.jafc.2c07453

**Published:** 2023-02-27

**Authors:** Davide Corinti, Lucretia Rotari, Maria Elisa Crestoni, Simonetta Fornarini, Jos Oomens, Giel Berden, Aura Tintaru, Barbara Chiavarino

**Affiliations:** † Dipartimento di Chimica e Tecnologie del Farmaco, Sapienza Università di Roma, Piazzale Aldo Moro 5, 00185 Roma, Italy; ‡ FELIX Laboratory, Institute for Molecules and Materials, Radboud University, Toernooiveld 7, Nijmegen 6525ED, Netherlands; § CNRS, Centre Interdisciplinaire de Nanoscience de Marseille, CINaM UMR 7325, Aix Marseille University, Marseille 13288, France

**Keywords:** flavanones, structural elucidation, conformational analysis, tandem mass spectrometry, isomeric discrimination, IRMPD action spectroscopy, naringenin

## Abstract

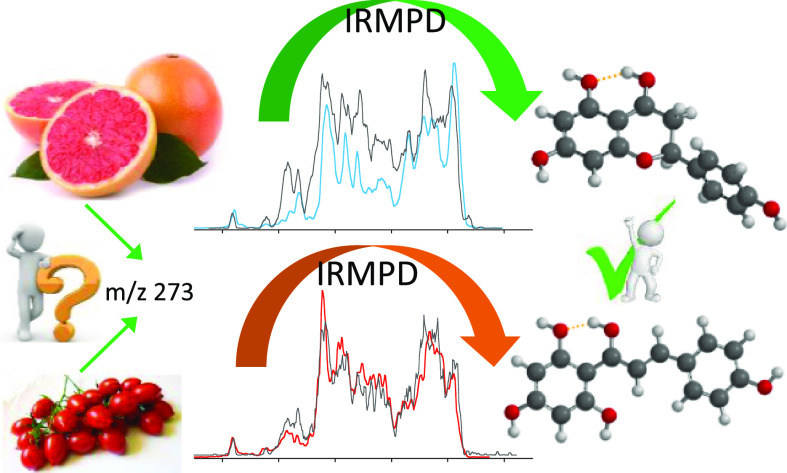

Naringenin (Nar) and its structural isomer, naringenin chalcone (ChNar), are two natural phytophenols with beneficial health effects belonging to the flavonoids family. A direct discrimination and structural characterization of the protonated forms of Nar and ChNar, delivered into the gas phase by electrospray ionization (ESI), was performed by mass spectrometry-based methods. In this study, we exploit a combination of electrospray ionization coupled to (high-resolution) mass spectrometry (HR-MS), collision-induced dissociation (CID) measurements, IR multiple-photon dissociation (IRMPD) action spectroscopy, density functional theory (DFT) calculations, and ion mobility-mass spectrometry (IMS). While IMS and variable collision-energy CID experiments hardly differentiate the two isomers, IRMPD spectroscopy appears to be an efficient method to distinguish naringenin from its related chalcone. In particular, the spectral range between 1400 and 1700 cm^–1^ is highly specific in discriminating between the two protonated isomers. Selected vibrational signatures in the IRMPD spectra have allowed us to identify the nature of the metabolite present in methanolic extracts of commercial tomatoes and grapefruits. Furthermore, comparisons between experimental IRMPD and calculated IR spectra have clarified the geometries adopted by the two protonated isomers, allowing a conformational analysis of the probed species.

## Introduction

A comprehensive identification of metabolites in complex biological matrices is of utmost importance to identify the molecular basis of their nutraceutical, biological, and pharmaceutical properties.^1^ To this end, numerous analytical techniques are exploited.^2^ Among them, mass spectrometry (MS) represents the method of choice for metabolite profiling, offering high accuracy, specificity, and sensitivity.^3,4^ However, MS analysis is not always able to distinguish between isomeric forms, so a multidimensional approach is required to obtain a clear assignment of the observed species. This notion appears particularly true concerning families of compounds such as flavonoids, flavones, and related molecules, which are highly populated in biological matrices and possess a vast isomeric variability.^5^ Flavonoids are an important class of phytochemicals, ubiquitous in plants, fruits, vegetables, flowers, cereals, and plant derivatives such as wines, teas, honey, and bee pollen.^6^ These natural polyphenols possess an extensive range of pharmacological activities, comprising antioxidative, anti-inflammatory, anticancer, antimutagenic, antiviral, and anti-ischemic effects. They are mainly present as glycosides, but free aglycones are also found in many plants. Due to their wide range of biological activities and the existence of numerous natural isomers, many efforts have aimed at differentiating and characterizing individual aglycones by tandem mass spectrometry.^7−10^ In particular, collision-induced dissociation (CID) mass spectrometry studies have successfully characterized several isomeric flavonoids belonging to distinct subgroups, through their different fragmentation patterns.^11^ However, it was not possible to apply this powerful technique to the direct discrimination of isomeric chalcones and flavanones due to their similar behavior under CID.^7,12^ Particularly, the discrimination of naringenin (Nar) and its structural isomer, naringenin chalcone (ChNar), in complex mixtures by MS appears to be a difficult task, without preliminary separation. In fact, the reference CID mass spectra for Nar and ChNar recorded either in positive or negative mode show identical fragmentation patterns.^13,14^

**Figure 1:**
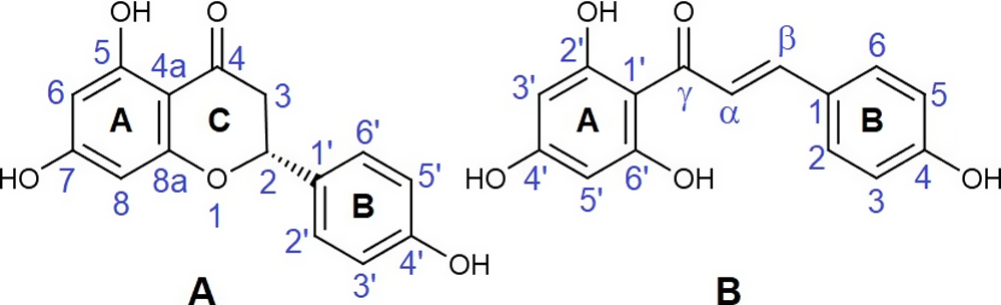
Schematic representation and numbering schemes of (A) naringenin and (B) naringenin chalcone. The numbering of the atoms is reported according to convention.

Nar (4′,5,7-trihydroxyflavanone) belongs to the flavanone group, and is a natural antioxidant abundant, especially in citrus fruits with numerous health-promoting effects.^15,16^ Nar is produced by stereo-specific isomerization catalyzed by chalcone isomerase from ChNar ((2E)-3-(4-hydroxyphenyl)-1-(2,4,6-trihydroxilphenyl)-2-propen-1on).^17,18^ Thus, the two species can be both present in many vegetables. Structurally, Nar shows the typical C6–C3–C6 flavan nucleus, formed by two aromatic rings (A and B) joined by a 2,3-dihydro-4-pyrone ring (C), as shown in Figure 1. There are numerous theoretical and experimental studies in the literature that have described the structure of neutral naringenin.^19−21^ The C-ring adopts a flattened chair-type conformation, in which the chiral C2 atom deviates from the plane defined by the other five atoms of the ring and binds mainly in the equatorial position of the B-ring. In nature, Nar is found predominantly as the (*S*)-enantiomer.^17^ On the other hand, ChNar is an open-chain flavonoid, in which the two aromatic rings A and B are joined by an α,β-unsaturated carbonyl system (Figure 1). The α,β-double bond of natural ChNar is always in *trans* configuration, while the *cis* isomer is reported to be extremely unstable. With respect to Nar, the absence of the central C-ring in ChNar allows internal rotations around the single bonds C1′–Cγ, Cγ–Cα, and Cβ–C1. From a conformational and energetic point of view, the most important rotation is around the Cγ–Cα single bond, leading to two distinct conformers referred to as *s-cis* and *s-trans*, depending on the relative position of the keto group, Cγ=O, and the double bond Cα=Cβ with respect to the single bond Cγ–Cα. Previous theoretical conformational studies on neutral chalcones have indicated that the *s-cis* conformation is the most stable geometry, with an energy gap to the *s-trans* species of approximately 24.5 kJ mol^–1^.^22,23^ Generally, chalcones in the *s-cis* conformation assume a relatively planar geometry. In contrast, the *s-trans* conformers are definitely nonplanar.^23^ In both Nar and ChNar, there is a strong intramolecular hydrogen bond between the carbonyl oxygen atom and the H atom of the adjacent hydroxyl group on the A ring.

Because Raman and IR spectra of neutral Nar and ChNar reported in the literature are different,^24^ it was decided to sample the protonated forms of both isomers by IR multiple-photon dissociation (IRMPD) spectroscopy. IRMPD is an “action” spectroscopy technique that allows one to obtain IR spectra of ionic species mass-isolated in the storage cell of a mass spectrometer.^25−28^ Comparison of experimental spectra with those obtained by DFT calculations of plausible structures can be used to discriminate isomers and investigate molecular properties, conformational analysis, and binding interactions. This strategy has been applied to a large diversity of biomolecular ions and metal adducts, such as nucleotides,^29−31^ modified amino acids and peptides,^32−34^ metabolites,^35−37^ polyphenols,^38−42^ and chalcones.^43^

The main purpose of this contribution is to determine structural features and conformational distribution of protonated Nar and ChNar isolated in the gas phase by exploiting a combined approach based on electrospray ionization (ESI) coupled to (high-resolution) mass spectrometry, IRMPD action spectroscopy, theoretical DFT calculations, and ion mobility-mass spectrometry (IMS). This study was carried out on protonated species to avoid possible naringenin/naringenin chalcone isomerization during the ionization processes in the ESI source or solution. It has in fact been reported that an equilibrium in chalcone—flavanone—isomerization occurs promptly in neutral and alkaline media, thus behaving as proteiform species.^12,24,44^ In the proposed equilibrium mechanism, deprotonation on the C2′ hydroxyl group of naringenin chalcone is followed by the cyclization reaction yielding the enolate form of naringenin.^24^ Furthermore, IRMPD spectroscopy was also used to investigate the nature of the metabolite present in methanolic extracts of commercial tomatoes and grapefruits, chosen for their distinct flavonoid content.

## Materials and Methods

### Chemical Reagents and Materials

Naringenin and Naringenin chalcone (purity ≥ 95%) are research-grade commercial products (Merck Life Science S.r.l. Milan, Italy) and were used as received. Italian cherry tomatoes (*Solanum lycopersicum* L.) cultivar “datterino” and red grapefruits (*Citrus Paradisi*) cultivar “Star Ruby” were purchased from a local grocery store in Rome, Italy. Gaseous ions [Nar + H]^+^ and [ChNar + H]^+^, both at *m/z* 273, were obtained by electrospray ionization mass spectrometry (ESI-MS) in positive ion mode, through direct infusion by a syringe pump of a solution (10 μM) of either Nar or ChNar in acidified (1% HCOOH) methanol to favor protonation. Naringenin and Naringenin chalcone has been extracted by maceration from grapefruit and tomato peel.^45−47^ Details of the tomato and grapefruit sample preparation are described in the Supporting Information.

### High-Resolution FT-ICR-MS

High-resolution mass analyses were performed using a Bruker BioApex 4.7T Fourier transform ion cyclotron resonance (FT-ICR) mass spectrometer equipped with an Apollo I ESI source, (FT-ICR lab, Sapienza Università di Roma), with internal calibration (ensuring Δ*m* < 2 ppm).^48^

### CID Experiments

CID experiments at variable energy were made using a 2000 Q-Trap instrument (Applied Biosystems), a commercial hybrid triple quadrupole linear ion trap mass spectrometer with a Q1q2Q_LIT_ configuration. The operating procedures are reported in the Supporting Information. Phenomenological threshold energies (TEs) of the various fragment ions can be obtained by CID at variable collision energy as described previously^48−51^ (see the Supporting Information for more details).

### Ion Mobility Experiments

Traveling wave ion mobility-mass spectrometry (TWIMS-MS) experiments were performed with a Synapt G2 HDMS quadrupole/time-of-flight mass spectrometer (Waters, Manchester, UK) equipped with an ESI source. The operating details are described in the Supporting Information.

### IRMPD Experiments

Sample solutions were directly infused in a 3D quadrupole ion trap (QIT) mass spectrometer (Bruker, Amazon Speed ETD, Bremen, Germany) coupled to the beamline of the free-electron laser for Infrared eXperiments (FELIX),^52^ as explained in the Supporting Information. During an IRMPD “action” spectroscopy experiment, the absorption of multiple resonant IR photons leads to an increase in the internal energy of the analyte, causing its unimolecular dissociation and, consequently, the appearance of fragment ions in the mass spectrum. To prevent excessive depletion of the parent ions and to minimize the formation of fragment ions below the low mass cut of the QIT, the IR spectra were recorded at several levels of laser pulse energy attenuation.^53^

### Computational Details

To obtain the lowest energy conformations of candidate isomers of the sampled [Nar + H]^+^ and [ChNar + H]^+^ ions, two different strategies were adopted as illustrated in the Supporting Information. Optimized geometries, thermodynamic properties (electronic energy values, zero point energy (ZPE) and thermal corrections, entropies, and free energies at 298 K), and harmonic vibrational frequencies were calculated in the gas phase using density functional theory with the B3LYP functional and 6-311++G(d,p) basis set.^54,55^ All quantum chemical calculations were performed with the Spartan′16 software package. CCSs were calculated from the B3LYP-D3/6-311++G(d,p) optimized structures with the MobCal software as appropriately modified by Kim et al. for mobility separations employing N_2_ as drift gas.^56,57^

## Results and Discussion

### HR-MS, CID Experiments

MS is extensively used for the characterization of flavonoid compounds in foods. However, discriminating between naringenin (Nar) and its chalcone isomer (ChNar), directly infused into a mass spectrometer without prior (U)HPLC separation, is a challenging task.^13,58−60^ The high-resolution mass spectrum of protonated naringenin recorded in the FT-ICR mass spectrometer displays the monoisotopic peak at *m/z* 273.07647 in good agreement with the calculated elemental composition [C_15_H_13_O_5_]^+^ (error + 2.6 ppm), as reported in Figure S1.

Under CID conditions, the MS/MS spectrum of protonated naringenin [Nar + H]^+^ reported in Figure S2 shows four fragment ions assigned to characteristic cleavages of the C-ring in the flavanone, as presented in Scheme S1.^61,62^ The two primary fragment ions at *m/z* 153 and 147 correspond to the ^1,3^A^+^ and ^1,4^B^+^–2H cleavages, respectively. Two other ions, namely *m/z* 119 and 91, are due to subsequent losses of one and two CO molecules from the^1,4^B^+^–2H fragment. Threshold energy values are not directly measurable;^49,63,64^ however, phenomenological TEs of the two competitive and sequential fragmentation paths can be obtained from comparative analysis, as shown in the breakdown curves of Figure S3 and reported in Table S1. The relatively low TE values of 0.70 and 1.0 eV found for the formation of *m/z* 147 and 153, respectively, confirm the facile cleavage of the C-ring in flavanones.^61^ Subsequent losses of CO require more energy, as indicated by TE values of 2.30 and 3.30 eV found for ions at *m/z* 119 and 91, respectively. As expected from the literature, the behavior of [ChNar + H]^+^ under CID displays quite identical characteristics.^13,14^ The MS/MS spectrum of [ChNar + H]^+^ reveals the same four fragment ions observed in the case of [Nar + H]^+^ (Figure S2, orange trace), namely ions at *m/z* 153, 147, 119, and 91 with phenomenological TEs (Figure S4 and Table S1) of 1.15, 1.00, 2.50, and 3.60 eV, respectively. All TE values found for the fragmentation paths of ChNar are slightly higher than those obtained for Nar; however, these small differences do not produce substantial differences in the spectra of the two isomers.

### IRMPD and Computational Study

#### Comparison of IRMPD Spectra for [Nar + H]^+^ and [ChNar + H]^+^

**Figure 2:**
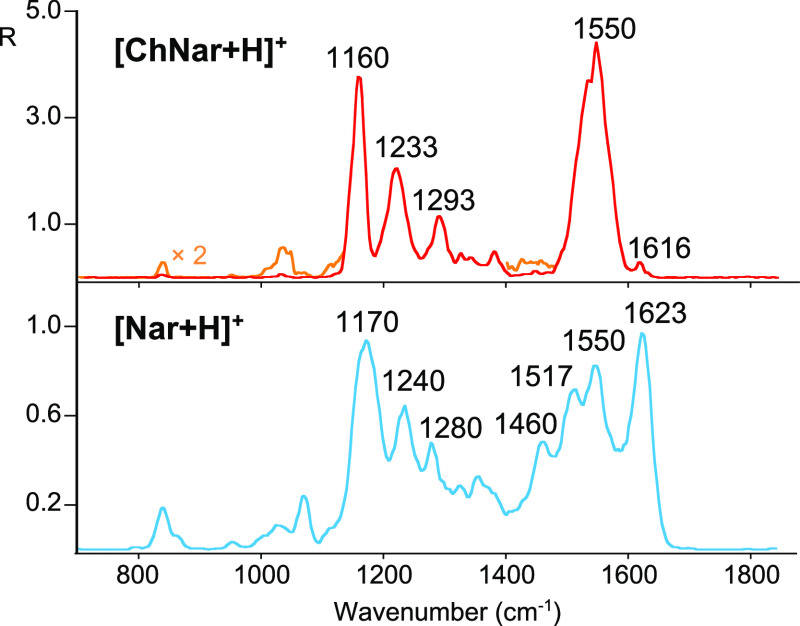
Comparison between the IRMPD spectra of protonated naringenin (light blue trace) and naringenin chalcone (red trace, magnified by a factor of 2, as indicated, in the yellow trace).

To discriminate between the protonated forms of the two isomers, ions at *m/z* 273, generated by ESI-MS from the methanolic solution of naringenin or naringenin chalcone, were isolated in the mass spectrometer and interrogated by IRMPD spectroscopy in the fingerprint region (700–1850 cm^–1^). Comparison of the IRMPD spectra of [Nar + H]^+^ and [ChNar + H]^+^ shown in Figure 2 indicates the spectral range between 1400 and 1700 cm^–1^ to be specific and appropriate to distinguish between the two isomers. In this range, the IRMPD spectrum of protonated ChNar displays only one strong absorption at 1550 cm^–1^, while the experimental spectrum of [Nar + H]^+^ presents four major bands at 1460, 1513, 1550, and 1623 cm^–1^. In particular, this latter absorption, predominant in the IR spectrum of [Nar + H]^+^, together with the band at 1460 cm^–1^ can be chosen as vibrational signatures of naringenin in foodstuff analysis. Conversely, a predominant feature at 1550 cm^–1^ could be indicative of the presence of ChNar. In contrast, the spectral range between 700 and 1400 cm^–1^ is not suitable for differentiating between the two species. In this interval, both isomers present similar characteristics, namely [Nar + H]^+^ exhibits three pronounced absorptions at 1170, 1240, and 1280 cm^–1^, which are superimposable onto the IR bands observed at 1160, 1233, and 1293 cm^–1^ in the spectrum of [ChNar + H]^+^. Weaker features at ca. 840, 957, 1040, and 1326 cm^–1^ are also present in the IRMPD spectra of both isomers. The assignment of the experimental vibrational modes of [Nar + H]^+^ and [ChNar + H]^+^ will be discussed in the next two sections.

Another distinctive aspect of the IRMPD investigations of Nar and ChNar lies in the different photofragmentation yields, R, of the two isomers. Upon IRMPD of both species, the fragment ions observed are identical to the ones seen in the CID experiments. Nonetheless, the R value of protonated chalcone is five times higher than that of naringenin, despite only slightly higher TE values of [ChNar + H]^+^. Of course, this finding is not a conclusively discriminating facet, but it could be taken into consideration in food analysis as a valuable parameter to distinguish between the two isomers.

### Protonation Site and Conformational Analysis

#### Protonated Naringenin

**Figure 3:**
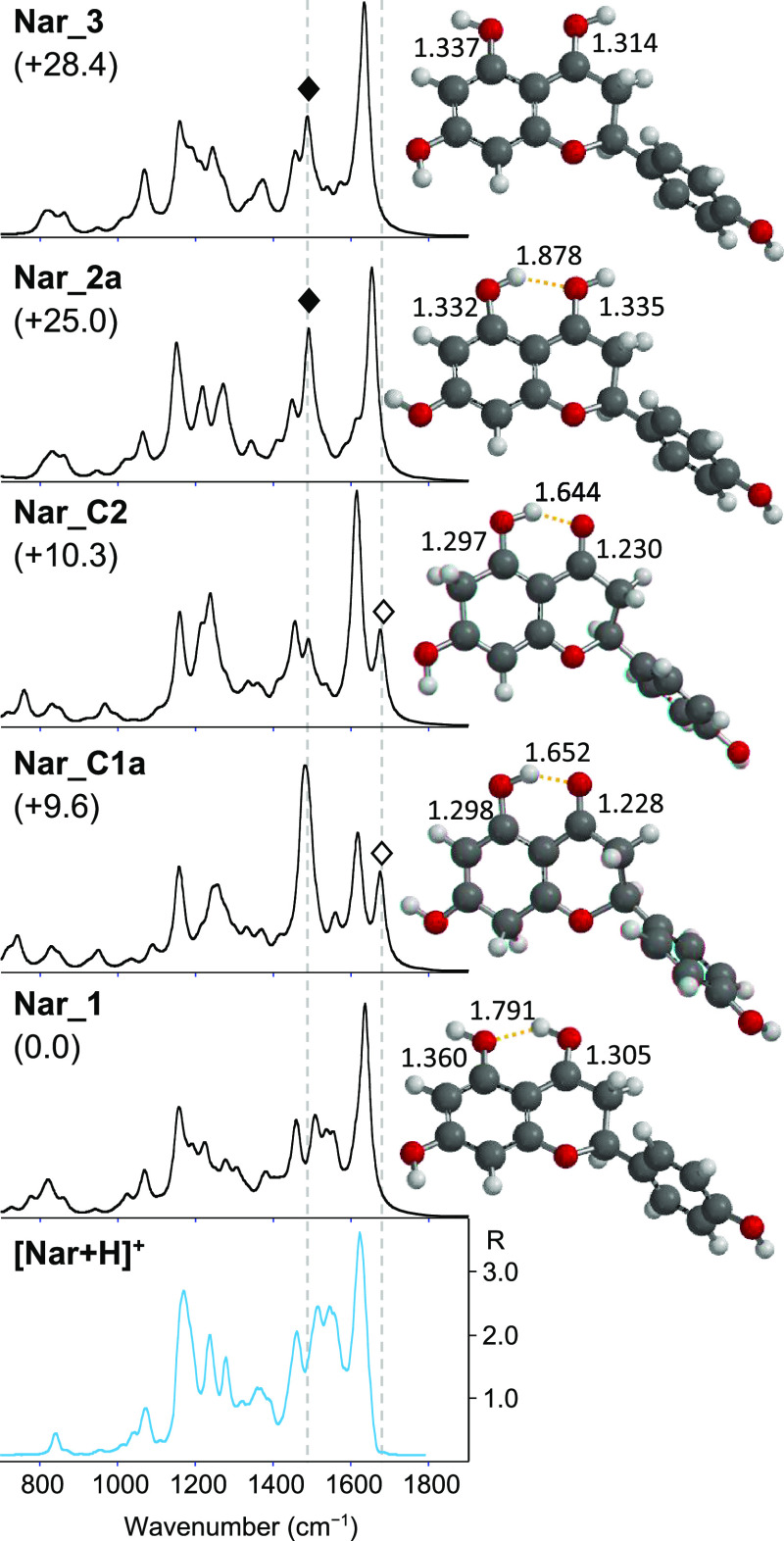
IRMPD spectrum of [Nar + H]^+^ (bottom panel) compared with calculated IR spectra of ***Nar_1***, ***Nar_C1a***, ***Nar_C2***, ***Nar_2a***, and ***Nar_3***, whose optimized structures are on the right. Relative free energies at 298 K are reported in kJ mol^–1^. Distances are given in Å. An open diamond indicates the calculated C4=O stretching mode at 1676 cm^–1^ for ***Nar_C1a*** and ***Nar_C2***, and a black diamond indicates the C5–OH stretching mode expected at 1490 cm^–1^ for ***Nar_2a*** and ***Nar_3***. The y-scale of calculated IR intensity ranges up to 1200 km mol^–1^.

Due to its structure, naringenin presents several possible protonation sites, namely the carbonyl oxygen in C4, the three hydroxyl oxygens in C5, C7, and C4′, and carbon atoms such as C6 and C8 of the A-ring. To elucidate the [Nar + H]^+^ structure, its experimental spectrum is compared with the calculated IR spectra of potential protonated naringenin structures. The structures, relative free energies, and IR calculated spectra of selected isomers of [Nar + H]^+^ are presented in Figure 3 together with the IRMPD spectrum of [Nar + H]^+^. A complete set of optimized geometries investigated in this study is reported in the Supporting Information Figures S5–S9. Focusing first on the isomers protonated on the carbonyl oxygen (4–O), three families of conformers are conceivable, differing by the orientation of the hydroxyl groups in C4 and C5. The ***Nar_1*** family (Figures 3, S5, and S6) is characterized by an anticlockwise orientation of both hydroxyl groups (4 and 5). This orientation allows the formation of an intramolecular hydrogen bond between the hydrogen atom of the 4–OH and the oxygen in C5. Conversely, the clockwise alignment of both hydroxyl group in C4 and C5, typical of the ***Nar_2*** conformers (Figures 3 and S7, upper panel) yields a hydrogen bond between the hydrogen atom in C5 and the oxygen in C4, as observed in neutral naringenin.^20,21^ All ***Nar_1*** conformers are more stable than ***Nar_2*** species (***Nar_2a***, the more stable conformer among the ***Nar_2*** family, lies 25.0 kJ mol^–1^ higher in energy relative to the global minimum ***Nar_1***). A possible explanation for this difference in stability is that the counterclockwise orientation allows for a stronger intramolecular hydrogen bond than the clockwise arrangement, as previously observed in the case of protonated o-hydroxybenzaldehyde, where the most stable geometry is stabilized by the intramolecular H-bond occurring between the proton on the formyl group and the phenolic oxygen atom.^65^ In fact, in the counterclockwise orientation, it is the best hydrogen-bonding acceptor atom, i.e., the phenolic oxygen sp^3^, that binds to the hydrogen of the best hydrogen-bonding donor atom, i.e., the carbonyl oxygen sp^2^.^66,67^ Moreover, the calculated intramolecular hydrogen bond lengths of the ***Nar_1*** conformers are between 1.786 and 1.796 Å, which are significantly shorter than those for the ***Nar_2*** species varying between 1.878 and 1.883 Å. Finally, the ***Nar_3*** family, lying over 28.0 kJ mol^–1^ higher in energy relative to the ground-state minimum, presents the C5–OH and C4–OH group with anti-clockwise and clockwise orientation respectively (Figures 3 and S7, lower panel). This arrangement does not consent to any intramolecular H-bonding.

Protonation on aromatic carbon C8 or C6 yields stable isomers, ***Nar_C1*** and ***Nar_C2*** (Figures 3 and S8), with comparable stabilities. They possess the shorter intramolecular hydrogen bond, (computed lengths 1.652–1.644 Å) and the most stable conformers of these types of isomers, namely ***Nar_C1_a*** and ***Nar_C2***, are located at only 9.6 and 10.3 kJ mol^–1^, respectively, above the global minimum. As already reported for neutral naringenin,^20^ the C-ring of all considered conformers adopts a chair-type conformation where only the C2 atom is located out of the plane defined by the A and C rings. Bound to C2, the B-ring prefers the equatorial position with a dihedral angle τ_Nar_1, formed by C3–C2–C1′–C2′ atoms, varying between 50 and 60° in good agreement with the value of 60.18° measured by X-ray crystallography.^20^ The torsional angles τ_Nar_2, formed by C6–C7–O(7)–H(7) atoms, and τ_Nar_3 formed by C3′–C4′–O(4′)–H(4′), adopt values of either 0 or 180°, approximately. A complete scheme with all the species investigated (showing relative free energies, dihedral angles τ_Nar_1, τ_Nar_2, and τ_Nar_3, hydrogen bonding length and C–O lengths, total electronic energies, and thermal corrections) is provided in the Supporting Information (Tables S2–S4), together with the XYZ coordinates of the most stable conformer of each family of rotamers/isomers.

Looking at Figure 3, the IRMPD spectrum is well interpreted by the computed spectrum of ***Nar_1***. Conversely, any contribution of ring-protonated species in the sampled population can be excluded. As shown in Figures 3 and S10, the computed IR spectra for the ***Nar_C1*** and ***Nar_C2*** optimized structures are very different from the experiment. Moreover, the typical feature at 1676 cm^–1^ due to the stretching mode of the carbonyl involved in H-bonding, is missing. We may discard protonation on 7-OH or 4′-OH since the calculated free energy of ***Nar_4*** and ***Nar_5*** (Figure S9), representative isomers protonated on 7–OH and 4′–OH, are higher in energy compared with the global minimum (over 169 kJ mol^–1^). Moreover, their computed spectra are not compatible with the experimental IRMPD spectrum. To complete the study, also the protonation at the O1 position was examined; however, all efforts to optimize this geometry yielded an open C-ring structure, that was not further investigated. Comparison of the calculated spectra with the experimental one of [Nar + H]^+^, reported in Figures 3 and S11, leads us to exclude also any contributions from ***Nar-2*** and ***Nar-3*** family conformers in the ion population of [Nar + H]^+^. The calculated spectra of all conformers of ***Nar_2*** and ***Nar_3*** types present an important feature at ca. 1490 cm^–1^, corresponding to the C5–OH stretching, which does not appear in the experimental spectrum and in the calculated spectra of the ***Nar_1*** conformers. This vibrational mode occurs red-shifted at 1225 cm^–1^ for ***Nar_1*** conformers, as a consequence of the H-bond between 5–O and 4–HO. The anticlockwise alignment of ***Nar-1*** conformers elongates the C5–OH bond. The calculated bond lengths of C5–OH are 1.360, 1.332, and 1.337 Å for ***Nar_1***, ***Nar_2***, and ***Nar_3***, respectively. On the other hand, to allow the H-bond between 4–O and 5–HO, the C4–O bond length of ***Nar_2*** conformers is increased compared to the ***Nar_1*** species. As a result, the C4–OH stretching mode for ***Nar_1***, preserving its partial double bond character (1.305 Å), is expected at 1504 cm^–1^ and clearly visible in the experimental IRMPD spectrum of [Nar + H]^+^, while it is red-shifted at 1340 cm^–1^ in the calculated spectra of ***Nar_2*** (1.335 Å). Furthermore, the distinct experimental absorption band at 1623 cm^–1^ is well interpreted by the CC stretching mode (A ring) of ***Nar_1*** predicted at 1636 cm^–1^, while it appears blue-shifted at 1653 cm^–1^ in the calculated spectra of ***Nar_2*** species.

**Scheme 1:**
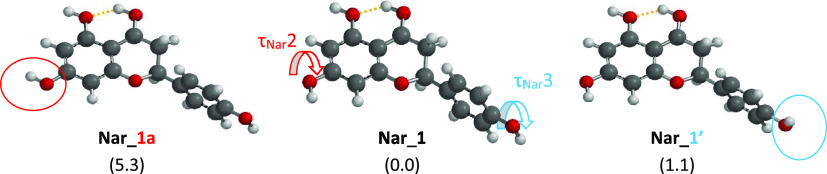
Exemplary Notation of Conformers

Having therefore confirmed that protonated naringenin adopts the anticlockwise alignment typical of ***Nar_1*** conformers, it is possible to estimate a conformational analysis on these geometries. Each family of structures includes multiple conformers differing in the arrangement of the hydroxyl group bound in C7–O or C4′–O in which the dihedral angles τ_Nar_2 and τ_Nar_3 can assume a value of 0 and 180°. Throughout this paper, the notation **na** indicates that the value of dihedral angle τ_Nar_2 is about 0° while the notation **n**′ means that the dihedral τ_Nar_3 has a the value of 0° (Scheme 1).

In general, for all conformers considered, the 180° rotation of C4′–OH has little influence on relative energies, yielding species with similar free energy as observed for neutral naringenin.^20^ Furthermore, in agreement with similar findings in an IRMPD study on deprotonated genistein^68^and with an IR study on neutral naringenin,^69^ the calculated IR spectra for each pair of such conformers are completely superimposable as can be noted in the calculated spectra IR collected in the Supporting Information (Figures S10–S12).

**Figure 4:**
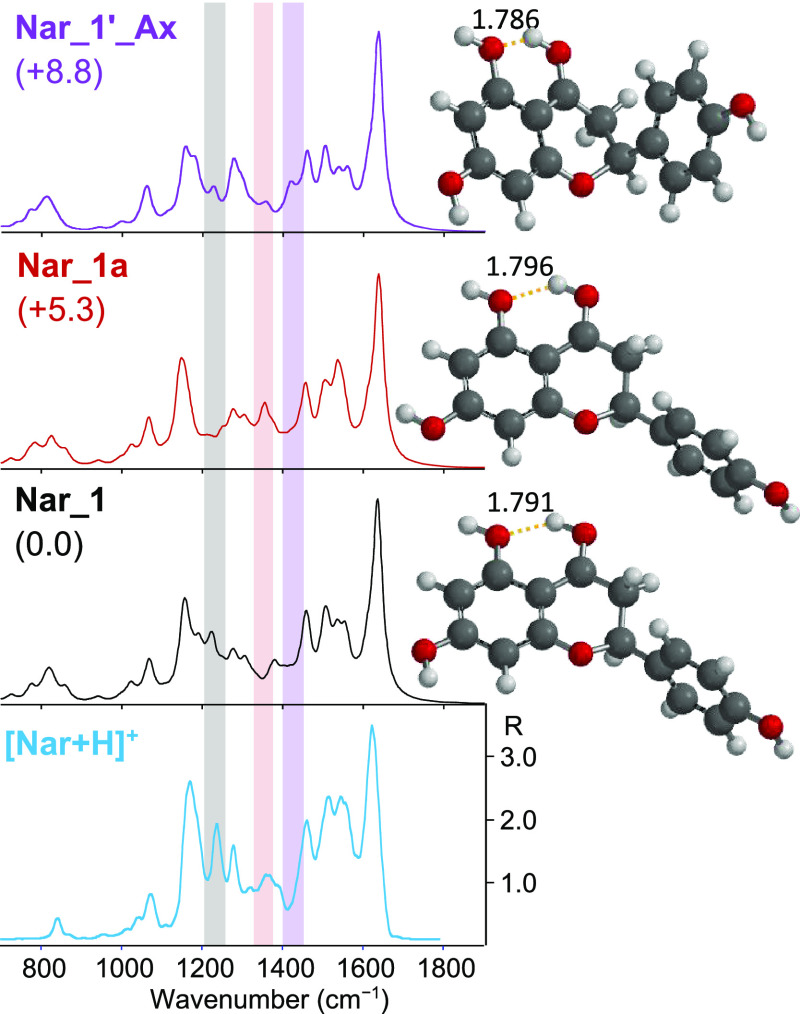
IRMPD spectrum of [Nar + H]^+^ (light blue profile) compared with calculated IR spectra of ***Nar_1***, ***Nar_1a***, and ***Nar_1_Ax*** (gray, red, and violet profiles, respectively), whose optimized structures are reported on the right. Distances are given in Å. Relative free energies at 298 K are reported in kJ mol^–1^. The y-scale of calculated IR intensity ranges up to 1200 km mol^–1^.

Focusing on the ***Nar_1*** family, the global minimum ***Nar_1*** shows an anticlockwise orientation of the three hydroxyl substituents of the A and C rings. The 180° rotation of the dihedral angle τ_Nar_3 leads to conformer ***Nar_1***′ lying only 1.1 kJ mol^–1^ higher in energy. The calculated energy barrier for the interconversion from ***Nar_1*** to ***Nar_1***′ is 16.5 kJ mol^–1^ as shown in Figure S13, where the potential energy surface (PES) of the isomerization paths between selected geometries of ***Nar_1*** is reported. This low rotational barrier allows isomerization between these minima in protonated naringenin as in the case of the neutral molecule. The single bond character of C4′–OH (calculated length of 1.355 Å), associated with the CO stretching mode computed and found in the IRMPD spectrum of [Nar + H]^+^ at 1275 and 1280 cm^–1^ respectively, further supports these findings. ***Nar_1a*** and ***Nar_1a***′ display a clockwise orientation of the C7 hydroxyl group and are at 5.3 and 6.3 kJ mol^–1^ in relative energy. The relatively high energy (30.1 kJ mol^–1^) of the transition state for ***Nar_1***/***Nar_1a*** interconversion, via rotation of the C7–OH bond (Figure S13), reflects the presence of an extensive π conjugation through the A ring between the protonated carbonyl and the para hydroxyl substituent, as recently observed for protonated *p*-hydroxybenzaldehyde.^65^ It follows that, compared to the C4′–OH, the C7–OH bond features an increased double-bond character with a calculated length of 1.332 Å, leading the CO stretching mode to be blue-shifted at 1459 cm^–1^, in good agreement with the experimental feature at 1460 cm^–1^ in the IRMPD spectrum of [Nar + H]^+^. Nevertheless, a comparison between the calculated spectra of ***Nar_1*** and ***Nar_1a*** with the IRMPD spectrum of protonated naringenin, presented in Figure 4, confirms the presence of both conformers in the ion population. In general, both calculated spectra of ***Nar_1*** and ***Nar_1a*** are in good agreement with the experimental IRMPD spectrum of [Nar + H]^+^. The experimental features at 1240 cm^–1^ (highlighted in gray in Figure 4), well predicted in ***Nar_1*** and ***Nar_1***′ computed spectra and not present in the ***a*** counterpart, and at 1356 cm^–1^ (highlighted in pink), matching only with the calculated spectra of ***Nar_1a*** and ***Nar_1a***′ rotamers, can be taken as vibrational signatures of both pairs of conformers. The vibrational modes of [Nar + H]^+^ can be assigned as described in Table S5, which lists the experimental IRMPD features together with the calculated IR bands of the global minimum ***Nar_1*** with the exception of the absorption at 1356 cm^–1^ explained with the calculated mode of ***Nar_1a***. To complete the conformational analysis of the ***Nar_1*** family, the conformers bearing the B-ring in the axial position were also considered. With respect to their equatorial counterparts, they differ only in the position of the C2 atom relative to the plane defined by the C and A ring and in the value of the dihedral angle τ_Nar_1 that falls between −172.02 and −176.30°. As in the case of neutral naringenin, the axial arrangement is less stable (>8.8 kJ mol^–1^) as compared to the ***Nar_1*** equatorial conformers. The optimized geometries are reported in Figure S6. The IR computed spectra of ***Nar_1_Ax*** (Figures 4 and S12) are quite similar to those of the ***Nar_1*** equatorial conformer with the exception of a small band calculated at 1415 cm^–1^ (highlighted in light violet in Figure 4) corresponding to the CH_2_ scissoring vibrational mode. This feature is not discernible in the experimental spectrum. The axial configuration does not appear to be present in the ion population, in agreement with a predicted Boltzmann distribution which indicate the equatorial conformers to be the main contributor (over 98%) to the ground-state population of neutral S-Naringenin.^20^

#### Protonated Naringenin Chalcone

**Figure 5:**
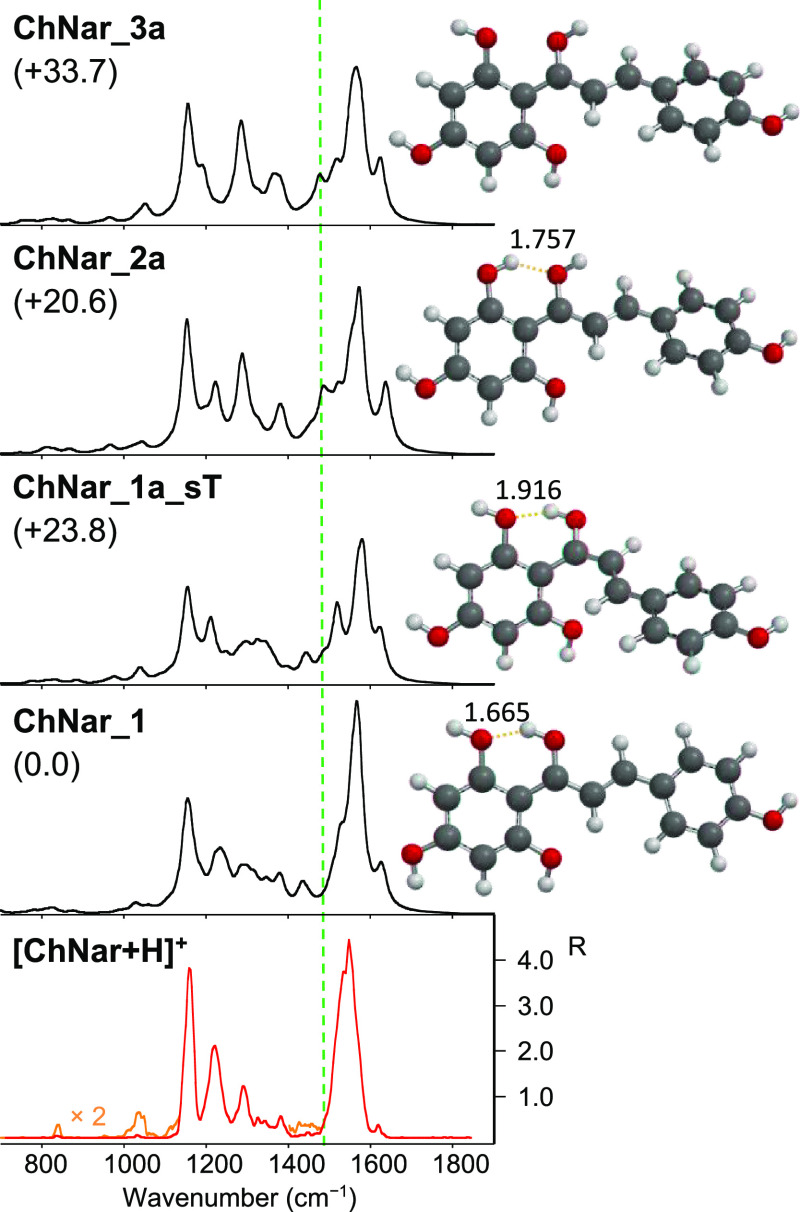
IRMPD spectrum of [ChNar + H]^+^ (bottom panel, in red) compared with calculated IR spectra of ***ChNar_1***, ***ChNar_1a_sT***, ***ChNar_2a***, and ***ChNar_3a***, whose optimized structures are reported on the right. Distances are given in Å. Relative free energies at 298 K are reported in kJ mol^–1^. The *y*-scale of calculated IR intensity ranges up to 3500 km mol^–1^.

Compared with naringenin, the presence of the α,β unsaturated bond and of an additional hydroxyl group, increases the possible sites of protonation of naringenin chalcone, whose structure is depicted in Figure 1. In addition to the carbonyl oxygen in Cγ, the three hydroxyl groups in C2′, C4′ (of the A-ring), and C4 (of the B-ring), and the aromatic 3′ and 5′ carbon atoms of the A ring, already considered in the case of naringenin, also the hydroxyl in C6′ and the double bond Cα=Cβ can be envisioned as protonation sites. The complete survey of all geometries considered is reported in the Supporting Information (Figures S14–18). Figure 5 shows the optimized geometries and their IR calculated spectra of selected isomers of [ChNar + H]^+^ protonated on the carbonyl oxygen (Cγ) plotted together with the IRMPD spectrum of [ChNar + H]^+^. Similarly to Naringenin, protonation on the carbonyl oxygen (Cγ) yields three different families of conformers distinguished by the motif of the hydroxyl groups at Cγ and C2′. The ***ChNar_1*** family (Figures 5 and S14 and S15) displays the anticlockwise arrangement of both hydroxyl groups, forming an intramolecular hydrogen bond between the hydrogen atom of the Cγ–OH and the O atom in C2′. On the other hand, hydrogen bonding occurs between the C2′OH group and the Cγ–O atom in the ***ChNar_2*** species characterized by the clockwise alignment of these two hydroxyl groups (Figures 5 and S16). In the ***ChNar_3*** conformers, the orientation of these neighboring hydroxyl groups does not allow any hydrogen bonding (Figures 5 and S16). As observed for naringenin, each family of conformers comprises multiple rotamers with comparable energies, depending on the disposition of the hydroxyl group in C4′ or C4 around the C–O bond. To be consistent with naringenin, the notation **na** indicates that the value of dihedral angle τ_Ch_4, formed by C3′–C4′–O(4′)–H(4′) atoms of the A ring, is about 0° while the notation **n**′ means that the dihedral τ_Ch_5, formed by C3–C4–O(4)–H(4) atoms of the B-ring, has a value of 0°.

Focusing on the *s-cis* configuration, again the anticlockwise pattern of hydroxyl groups in Cγ and C2′ increases the stability of the ion. The energy difference is more than 20 kJ mol^–1^ in favor of ***ChNar_1*** conformers versus the ***ChNar_2*** family. The enhanced intramolecular hydrogen bond, favored also by the short distance between H(γ) and O(2′) of about 1.665 Å, and the substantial planarity of the entire geometry with dihedral angles of about −10° for τ_Ch_1 (formed by C2′–C1′–Cγ–O(γ), −1,7° for τ_Ch_2 (O(γ)–Cγ–Cα–Cβ) and 0.3° for τ_Ch_3 (Cα–Cβ–C1–C2), play an important role in the global stabilization of the ***ChNar_1*** species. The complete survey with all species investigated (listing relative free energies, dihedral angles τ_Ch_1, τ_Ch_2, τ_Ch_3, τ_Ch_4, and τ_Ch_5 values and hydrogen bonding length, total electronic energies and thermal corrections, together with the cartesian coordinates of the most stable conformer of each family of rotamers/isomers) is provided in the Supporting Information (Tables S6 and S7). The clockwise motif increases the intramolecular hydrogen bond length, with the calculated distance between H(2′) and O(γ) for the ***ChNar_2*** conformers being ca. 1.760 Å. Furthermore, the co-planarity between the A- and B-ring is lost in both protonated ***ChNar_2*** and ***ChNar_3*** geometries characterized by the OH (γ) in clockwise orientation, due to steric hindrance between the two hydrogen atoms in γ and β position, as shown by τ_Ch_1, τ_Ch_2, τ_Ch_3 values reported in Table S6. Again, the lack of an intramolecular hydrogen bond in ***ChNar_3*** makes decreases their stability further, the whole conformers lying ca. 34 kJ mol^–1^ higher in energy. ***ChNar_1_sT*** conformers are the *s-trans* counterpart of ***ChNar_1***. The nonplanarity of the A-ring relative to the rest of the molecule in the *s-trans* conformation is essentially due to steric repulsion between the O and H atoms in 6′ and β position and illustrated by a τ_Ch_1 and τ_Ch_2 values of about −33 and 160°, respectively. The elongation of the intramolecular H-bond (1.900 Å) in ***ChNar_1_sT*** is a consequence of the A-ring nonplanarity, leading to a loss in stability of ca. 24 kJ mol^–1^. As in the case of naringenin, the combination of high free energy values (lying over 200 kJ mol^–1^ over the global minimum) and the noncorrespondence between computed and experimental spectra of ***ChNar_4*** and ***ChNar_5*** (Figure S17) allow us to exclude protonation on the hydroxyl oxygen atom in 4′- or 4- position. All attempts to optimize 6′–O protonated geometries resulted in a proton shift to the double bond. The so-formed isomers, which lie over 59 kJ mol^–1^ in energy with respect to the global minimum, are protonated at Cα as can be seen in ***ChNar_C3*** (Figure S18). Protonation on the aromatic 3′ or 5′ carbon atoms generates isomers with different stability depending on the orientation of the hydroxyl group ortho-positioned relatively to the protonated carbon (Figure S18). Among them, the most stable ***ChNar_C1*** and ***ChNar_C2a*** lie at 16.3 and 27.0 kJ mol^–1^ above the global minimum, ***ChNar_1***. However, the poor agreement between the experimental and computed spectrum of the carbon-protonated isomers (Figure S19) allows us to exclude their contribution to the ion population.

Looking at Figure 5, ***ChNar_1*** displays a theoretical IR spectrum in very good agreement with the experimental features of [ChNar + H]^+^. Additionally, the computed spectrum of ***ChNar_1a_sT*** shows similar features to the experimental one. The two distinct absorptions, expected at 1510 and 1576 cm^–1^ for ***ChNar_1a_sT***, can be accounted for in the intense experimental feature at 1550 cm^–1^ typical of [ChNar + H]^+^. Consequently, a contribution of *s-trans* conformers with anti-clockwise alignment cannot be excluded in the sampled [ChNar + H]^+^ population. Rotations around the C4–OH and C4′–OH bonds do not change the IR spectra of individual rotamers of a given family, as can be seen in the spectra depicted in Figures S20 and S21 in the Supporting Information. The calculated energy barriers for the interconversion from ***ChNar_1*** to either ***ChNar_1***′ or ***ChNar_1a*** are shown in Figure S22, where the potential energy surface (PES) of the isomerization paths between selected geometries of ***ChNar_1*** is reported.

In the calculated spectra of the geometries with OH(γ) clockwise oriented, namely ***ChNar_2*** and ***_3***, a feature is predicted at 1485 cm^–1^, which does not find a counterpart in the experimental spectrum. This band corresponds to the bending mode of the OH group in the γ position coupled with CC stretches. The same vibrational mode is expected at 1434 and 1439 cm^–1^ in the calculated spectra of ***ChNar_1*** and ***ChNar_1-sT*** and appears in the experimental spectra of [ChNar + H]^+^. Moreover, the vibrational Cα=Cβ stretching mode coupled to the bending of the OH group in γ position is predicted at 1283 cm^–1^ for all the ChNar optimized geometries protonated on the carbonyl oxygen (Cγ) and is particularly evident in the computed spectra of ***ChNar_2*** and ***_3***. The calculated intensity of this vibrational mode, however, seems better interpreted in the ***ChNar_1*** spectrum. These considerations lead us to exclude a significant contribution of ***ChNar_2*** and ***_3*** in the analyzed population. Therefore, from the analysis of the IRMPD spectrum, similar to our findings for naringenin, one may conclude that the two adjacent hydroxyl groups in the 2′ and γ positions adopt an anticlockwise arrangement in protonated naringenin chalcone. The vibrational modes of [ChNar + H]^+^ can be assigned as described in Table S8, which lists the experimental IRMPD features together with the calculated IR bands of the global minimum ***ChNar_1***. With this OH arrangement, while [Nar + H]^+^ adopts the equatorial conformation, [ChNar + H]^+^ may assume both the *s-cis* and the *s-trans* conformations. To confirm these conclusions, an ion mobility analysis was performed on [ChNar + H]^+^ and [Nar + H]^+^.

### Ion Mobility Experiments

Considering the possible different conformations of ***Nar_1***, ***Nar_1***′***Ax***, ***ChNar_1***, and ***ChNar_1a_sT***, ion mobility-mass spectrometry (IMS) could be useful to further characterize our samples. However, the two investigated compounds could not be discriminated via our IMS experiments. ESI (+)-IMS performed on an equimolar mixture of [Nar + H]^+^ and [ChNar + H]^+^ shows a unique drift time value, *D_T_* (mixture) = 2.63 ms (Figure S20 upper panel). In addition, both species when analyzed separately revealed identical *D_T_* values in the mobility diagram, with an arrival time of 2.63 ms for both [Nar + H]^+^ and [ChNar + H]^+^. However, the peak width observed in the mobilogram appears slightly different for the two isomers, analyzed in the same μ-molar concentration. While the mobility diagram of [Nar + H]^+^ shows a narrow peak, the [ChNar + H]^+^ one appears slightly broader. A similar broadening is observed for the [Nar + H]^+^/[ChNar + H]^+^mixture. This observation suggests that two distinct conformations might be present for [ChNar + H]^+^.

To support this hypothesis, the drift times were correlated to the collisional cross-section (CCS) of both isomers, using polyalanine oligomers to calibrate the mobility data,^70^ and compared with the calculated CCS for ***Nar_1***, ***Nar_1***′***Ax***, ***ChNar_1***, and ***ChNar_1a_sT***. The results are reported in Table S9. The CCS values of 160.8 and 160.1 Å^2^ calculated for ***Nar_1*** and ***ChNar_1***, respectively, are in good agreement with the identical values of 160.9 Å^2^ obtained for [Nar + H]^+^ and [ChNar + H]^+^, supporting that IMS analysis does not allow for differentiation between the two isomers. The calculated values for the axial conformer of ***Nar_1*** (162.4 Å^2^ for ***Nar_1Ax***) and *s-trans* conformer of ***ChNar_1*** (158.3 Å^2^ for ***ChNar_1_sT***) appear slightly different. There is a difference of ±1.8 Å^2^ from their more stable conformer. Clearly, this small difference is not sufficient to separate the two conformers in arrival time distribution data but may cause the broadening of the peak observed for [ChNar + H]^+^ but not for [Nar + H]^+^.

### Analysis of Metabolites in Complex Matrix

**Figure 6:**
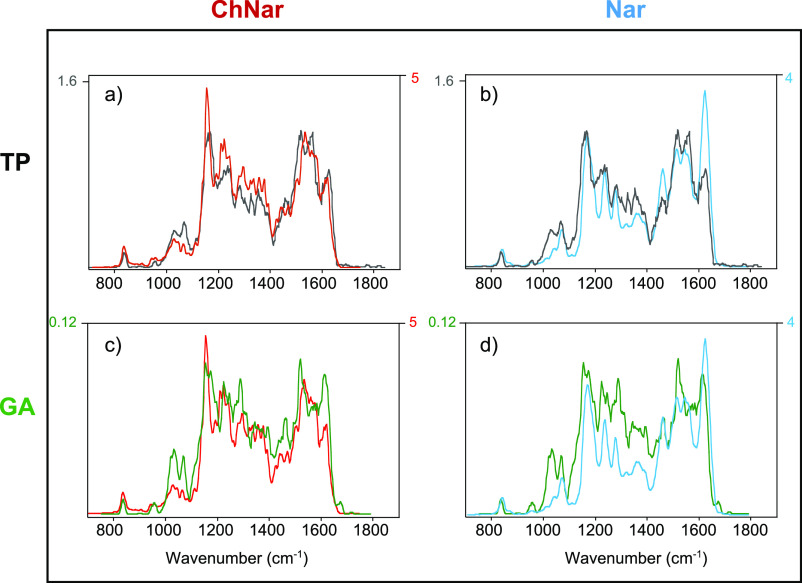
Comparison between the IRMPD spectra of mass-selected ions at *m/z* 273 from (1) the methanolic extract of tomato peel (upper panel (a and b), black trace) and (2) the methanolic extract of grapefruit albedo (lower panel (c and d), green trace) with standard solution of naringenin chalcone (on the left (a and c), red trace) and of naringenin (on the right (b and d) light blue trace) recorded at higher energy.

Considering the spectroscopic discrimination obtained for the standard solutions, we decided to assay by IRMPD spectroscopy the methanolic extracts of tomato peel (**TP**) and grapefruit albedo (**GA**), chosen for their high flavonoid content. Among the *Citrus* species, grapefruit is recognized as one of the fruits with the highest concentration of naringenin.^16,71^ In particular, its albedo is rich in naringin (naringenin-7-neohesperoside), one of the glycoside forms of naringenin. In contrast, fresh tomatoes are particularly interesting for their high content of naringenin chalcone, especially concentrated especially in the peel.^45^ However, also naringenin was found in tomato skin.^72,73^ For both phytochemical extracts, IRMPD spectroscopy was carried out on the ion at *m/z* 273 mass-selected in the ion trap from the mass spectrum of the complex matrix solutions directly electrosprayed into the mass spectrometer. Unfortunately, the full scan spectrum of both natural samples exhibited a low abundance of the compound at *m/z* 273. Despite the low signal-to-noise ratio of the mass-selected ion in the natural samples, we were able to record both IRMPD spectra, using higher pulse energies to increase the photofragmentation yield. For comparative purpose, knowing that IRMPD spectra may look different depending on the power level due to saturation effects, we also recorded the spectra of the reference compounds again at increased laser pulse energy as shown in Figure S24 where the high-power IRMPD spectra of the standard are plotted together with their IRMPD spectra recorded at lower laser energy. The resulting high-power IRMPD spectra of [Nar + H]^+^ and [ChNar + H]^+^ are shown in Figure 6 (in light blue and red, respectively), and compared with the IRMPD spectra of the ion at *m/z* 273 isolated from the **TP** extract (upper panel, black trace) and the methanolic solution of **GA** (lower panel, green trace). Focusing first on the IRMPD spectra of the two standards performed at low and high laser pulse energy, for [Nar + H]^+^, the improvements led to an overall increase in photofragmentation yield, R (by a factor of 5) without changing the spectrum profile (light blue trace versus blue trace, lower panel Figure S24). For [ChNar + H]^+^, on the other hand, due to the very high IRMPD yield (R) observed in the low-laser pulse energy spectrum, saturation is observed on the strong absorption at 1550 cm^–1^ in the high laser power spectrum. Consequently, all other bands appear to relatively increase as shown in the upper panel of Figure S24. Switching to the phytochemical extracts, the species at *m/z* 273 from **TP** extract yields the same photofragments at *m/z* 153, 147, 119, and 91, as observed for both [Nar + H]^+^ and [ChNar + H]^+^. To determine the presence of one or both isomers in the **TP** extract, the three IRMPD spectra can be compared, focusing mainly on the absorptions in the 1400–1800 cm^–1^ spectral range (Figure 6, upper panel). At first glance, the entire spectrum of the *m/z* 273 species from **TP** is very similar to the high laser energy spectrum of the ChNar standard (Figure 6, panel a). In particular, the pronounced absorption at 1550 cm^–1^ confirms the presence of ChNar in the sample of TP. However, the two absorptions typical of naringenin at 1460 and 1623 cm^–1^ emerge a little more in the spectrum of **TP** when compare to the ChNar standard spectrum. Therefore, in this particular sample of tomato skin, both isomers appear to contribute to the ion at *m/z* 273, with a higher prevalence of ChNar. Looking the IRMPD spectrum of the mass-selected ion at *m/z* 273 from GA extract, it appears similar to that of the [Nar + H]^+^ standard and shows clearly the two vibrational features at 1463 and 1617 cm^–1^, suggesting a predominant contribution of naringenin in **GA** (Figure 6, panel d). The unexpectedly intense feature at 1520 cm^–1^ found in the **GA** IRMPD spectrum can be attributed to some contribution of ChNar in the mass-selected ion population at *m/z* 273 (Figure 6, panel c). In conclusion, in the natural extracts analyzed by IRMPD, the presence of Nar and ChNar is likely. However, our experiments also suggest a clear prevalence of naringenin chalcone in the TP, while the GA contains mainly naringenin.

In summary, while IMS and CID experiments hardly differentiate the two isomers, IRMPD spectroscopy turned out as an efficient method for distinguishing naringenin from its chalcone. The present results confirm IRMPD spectroscopy in combination with DFT calculations to be a powerful tool not only to determine the structure and conformational space of food metabolites but also to design new approaches for targeted metabolomics.

## Supporting Information Available

Breakdown curves, optimized geometries, comparison between experimental and calculated IR spectra, potential energy surface (PES), operating details, complete survey of all geometries and Table of IRMPD absorptions and calculated vibrational frequencies (PDF)
