# Supplementary material for: Protonated Forms of Naringenin and Naringenin Chalcone: Proteiform Bioactive Species Elucidated by IRMPD Spectroscopy, IMS, CID-MS, and Computational Approaches
Source: J Agric Food Chem. 2023 Feb 27;71(9):4005–15. doi: 10.1021/acs.jafc.2c07453 (PMC9999425; doi:10.1021/acs.jafc.2c07453)
Supplement: Supplementary file 1 — jf2c07453_si_001.pdf [file jf2c07453_si_001.pdf]

Supporting informations

Protonated forms of Naringenin and Naringenin Chalcone: proteiform bioactive species elucidated by IRMPD spectroscopy, IMS, CID-MS, and computational approaches.

Davide Corinti[a], Lucretia Rotari[a], Maria Elisa Crestoni[a], Simonetta Fornarini[a], Jos Oomens[b], Giel Berden[b], Aura Tintaru[c] and Barbara Chiavarino[a]*

[a]Dipartimento di Chimica e Tecnologie del Farmaco, Sapienza Università di Roma, Piazzale Aldo Moro 5, 00185 Roma, Italy
[b]Radboud University, FELIX Laboratory, Institute for Molecules and Materials, Toernooiveld 7, Nijmegen, 6525ED, Netherlands
[c]Aix Marseille Univ, CNRS, Centre Interdisciplinaire de Nanoscience de Marseille, CINaM UMR 7325, Marseille, 13288, France
*corresponding author

Contents

Supporting figures……………………………………………………………………S2

Operating details……………………………………………………………………S25

Supporting tables……………………………………………………………………S27

XYZ coordinates of optimized geometries……………………………………………S36

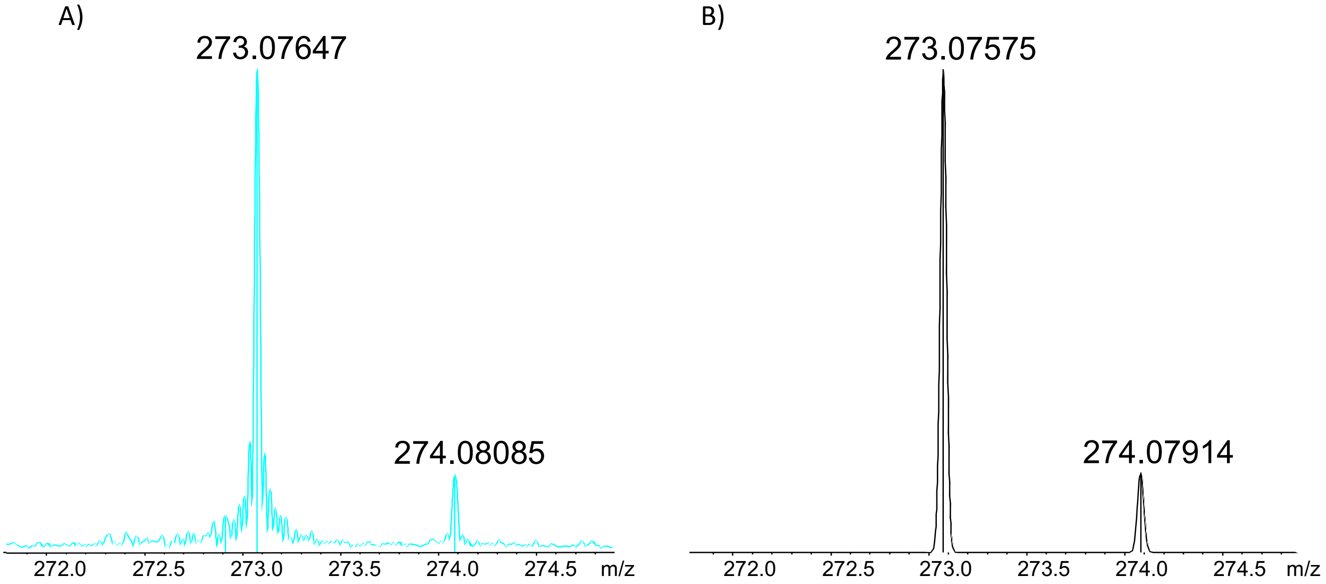

Figure S1. ESI FT-ICR mass spectrum of protonated naringenin, $[Nar+H]^+$. Experimental (panel A, light blue) and theoretical (panel B, black) isotope distribution.

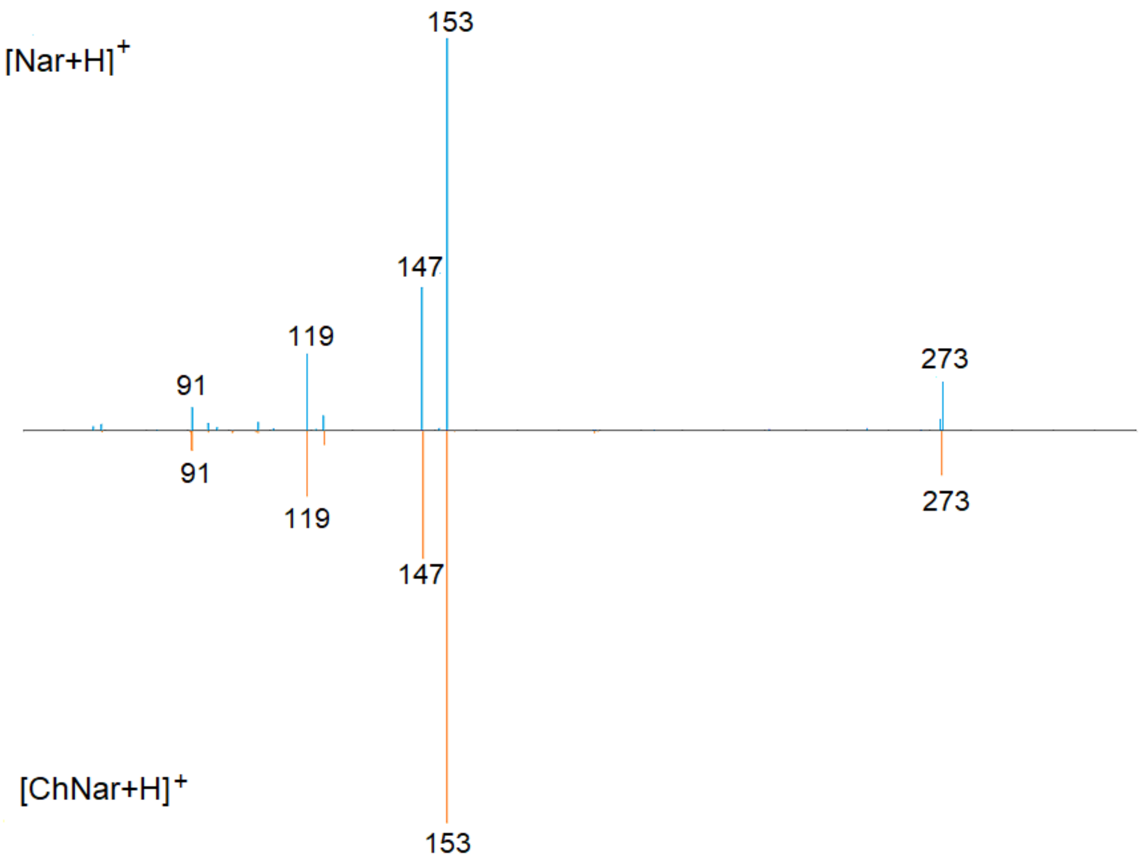

Figure S2. Comparison of CID mass spectra of [Nar+H]$^+$ and [ChNar+H]$^+$ (in blue and orange respectively), recorded at CE of 35 eV.

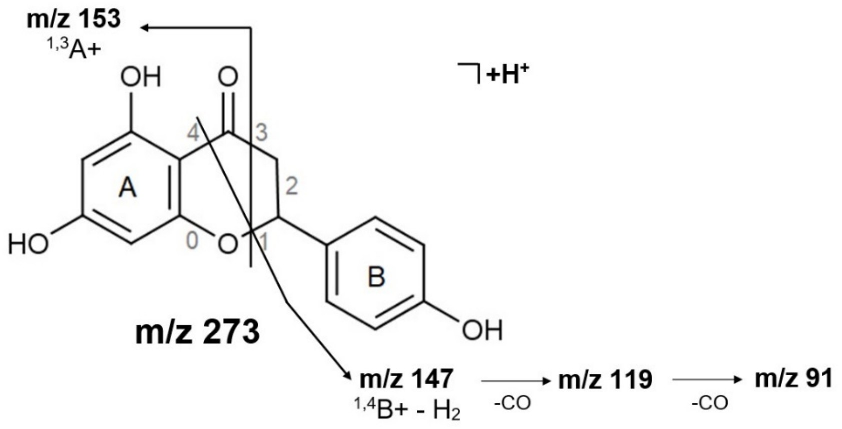

Scheme S1. Fragmentation pathways of protonated naringenin. The numbering of the C ring bonds is shown in gray.

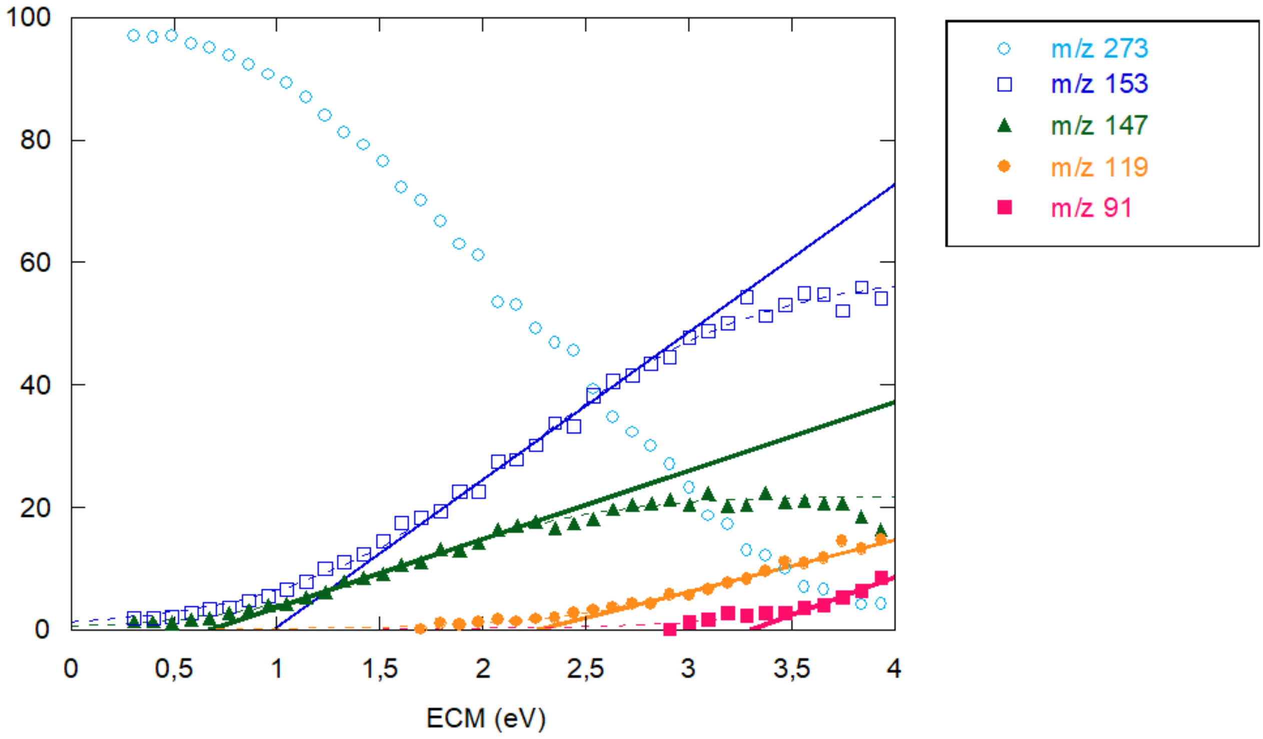

Figure S3. Breakdown curves for [Nar+H]$^+$ (*m/z* 273 light blue diamond).

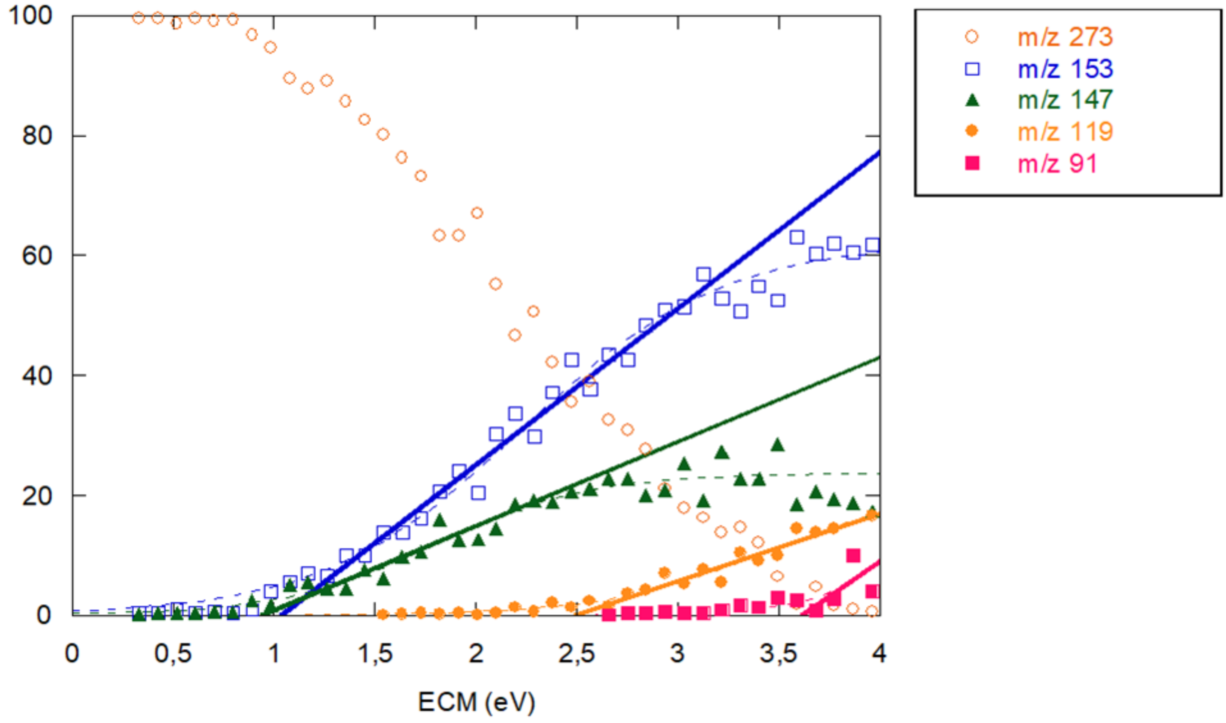

Figure S4. Breakdown curves for [ChNar+H]$^+$ (*m/z* 273 orange diamond)

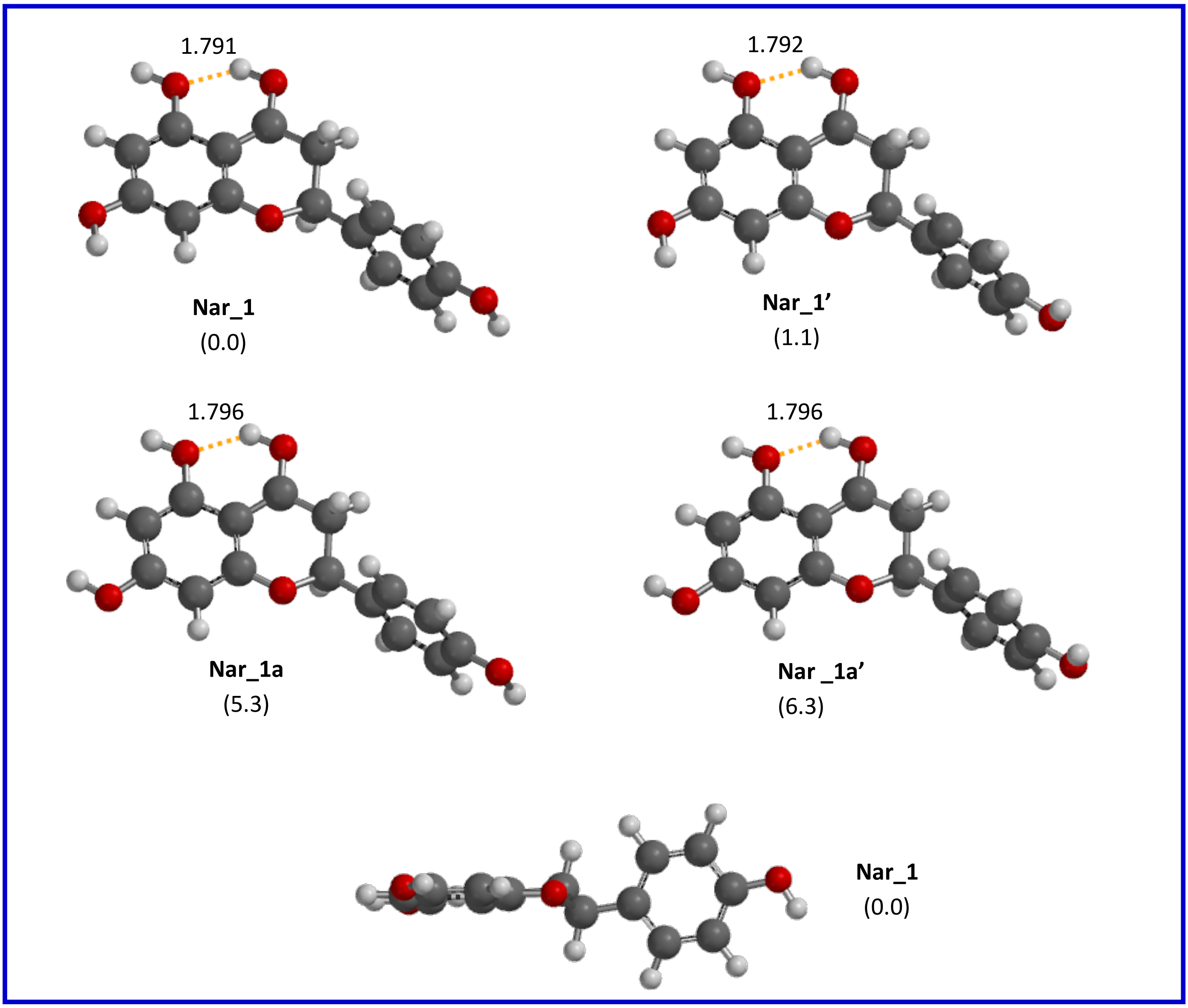

Figure S5. Optimized geometries and relative free energies (kJ mol^{-1}, in parenthesis) of conformers with 4-hydrophenyl group in equatorial position, belonging to the ***Nar_1*** family, calculated at the B3LYP/6-311++G(d,p) level of theory. The relative free energies are calculated with respect to the global minimum **Nar_1.** Distances are given in Å.

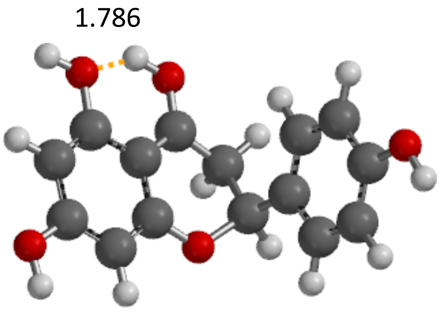

Nar_1'_Ax
(8.8)

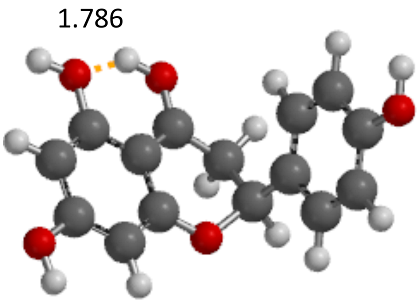

Nar_1_Ax
(9.0)

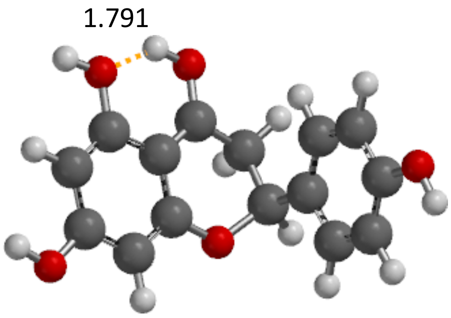

Nar_1a'_Ax
(13.8)

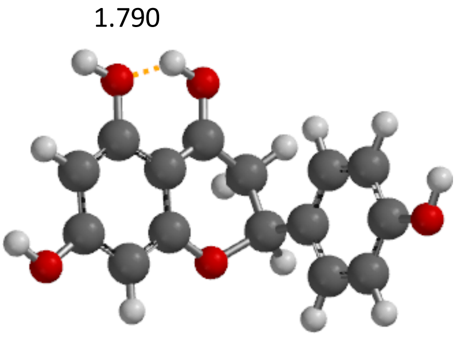

Nar_1a_Ax
(15.2)

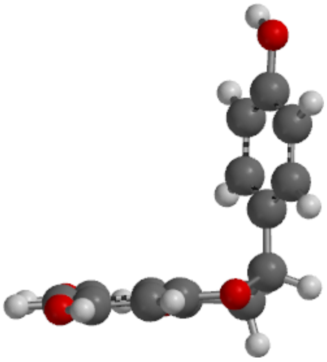

Nar_1'_Ax
(8.8)

Figure S6. Optimized geometries and relative free energies (kJ mol^{-1}, in parenthesis) of ***Nar_1*** family conformers with 4-hydrophenyl group in axial position, calculated at the B3LYP/6-311++G(d,p) level of theory. The relative free energies are calculated with respect to the global minimum **Nar_1.** Distances are given in Å.

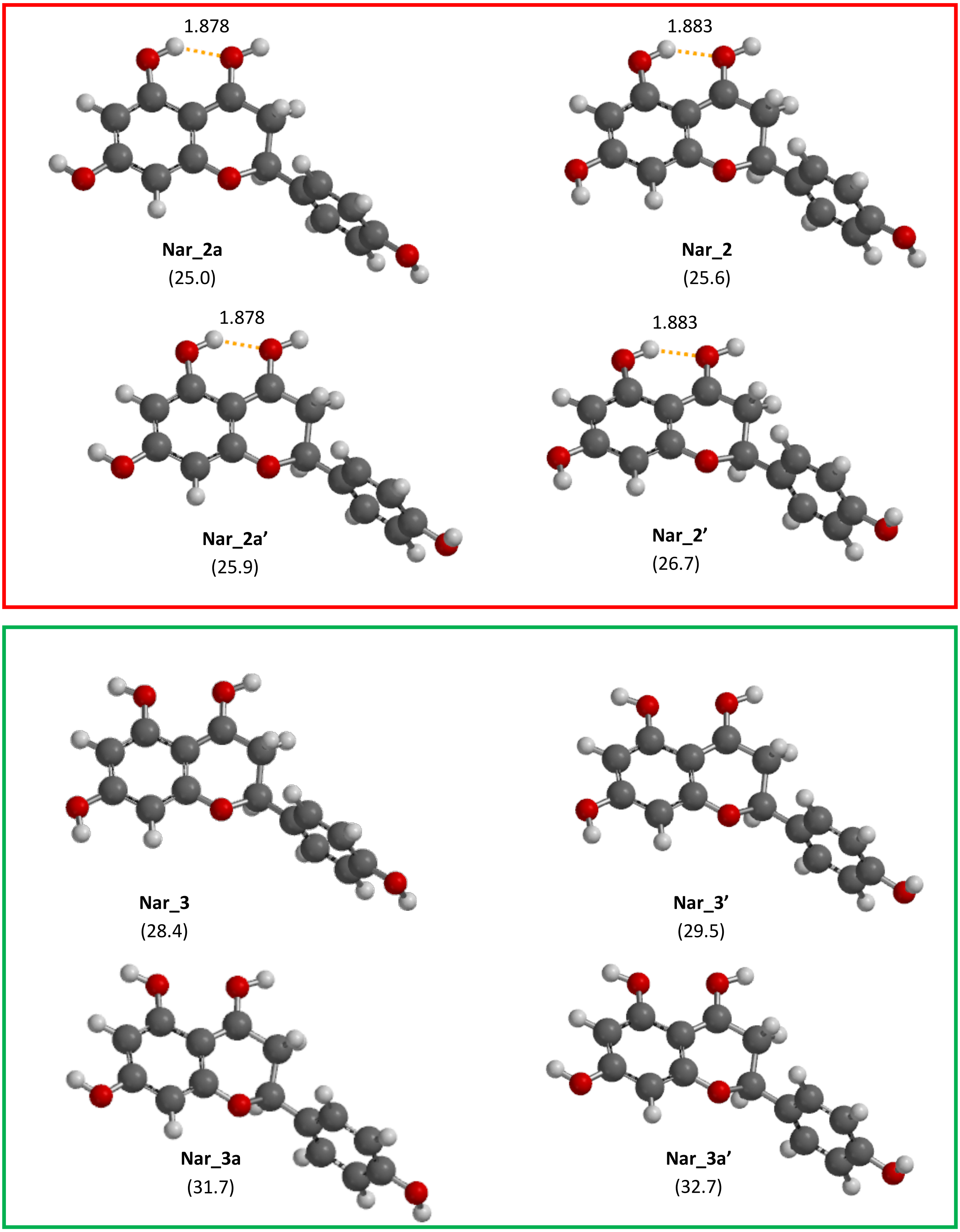

Figure S7. Optimized geometries and relative free energies (kJ mol^{-1}, in parenthesis) of conformers with 4-hydrophenyl group in equatorial position, belonging to the ***Nar_2*** (upper red panel) and ***Nar_3*** (lower, green panel) families, calculated at the B3LYP/6-311++G(d,p) level of theory. The relative free energies are calculated with respect to the global minimum **Nar_1.** Distances are given in Å.

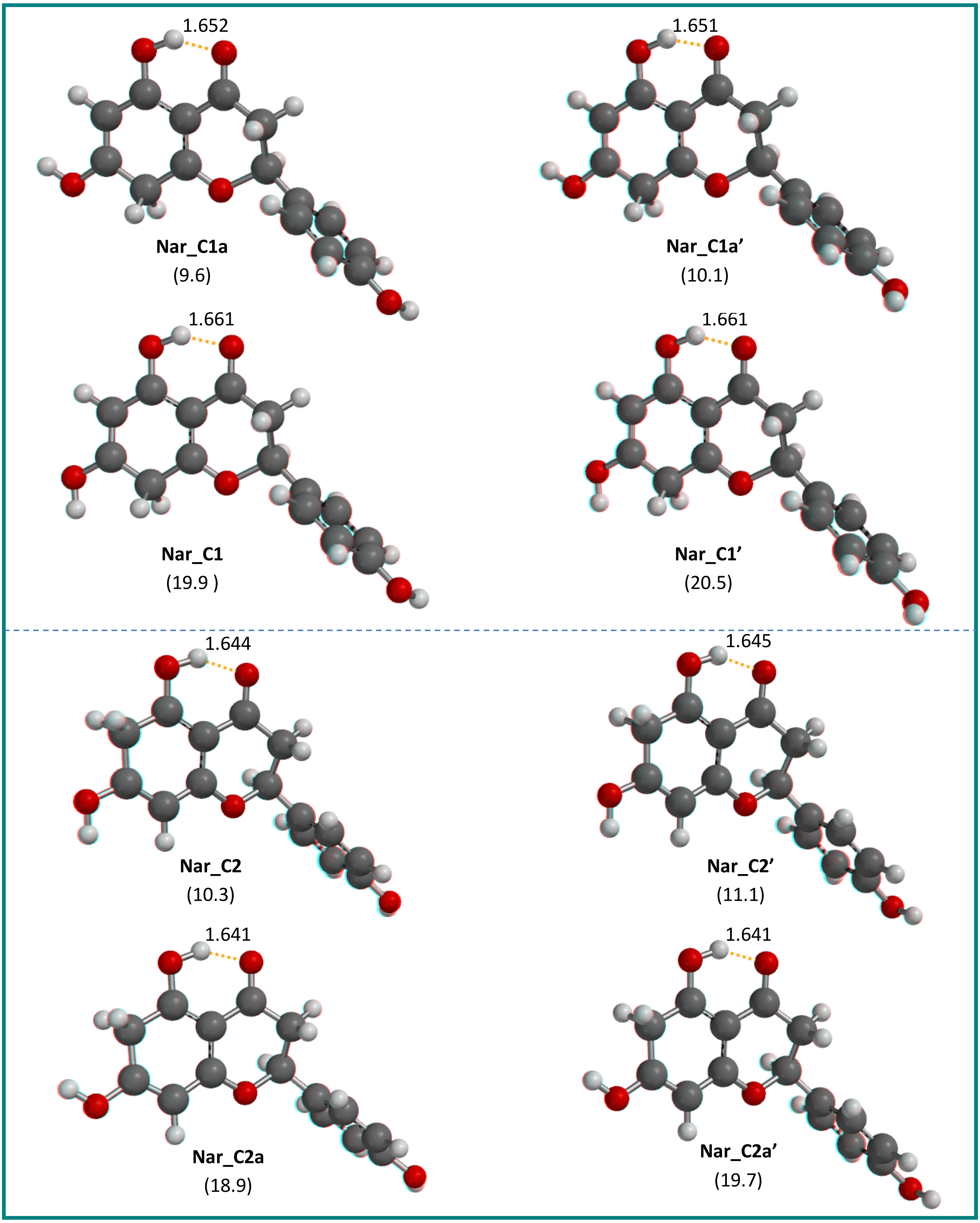

Figure S8. Optimized geometries and relative free energies (kJ mol^{-1}, in parenthesis) of conformers with 4-hydrophenyl group in equatorial position, belonging to the ***Nar_C1*** (upper panel) and ***Nar_C2*** (lower panel) families, calculated at the B3LYP/6-311++G(d,p) level of theory. The relative free energies are calculated with respect to the global minimum **Nar_1.** Distances are given in Å.

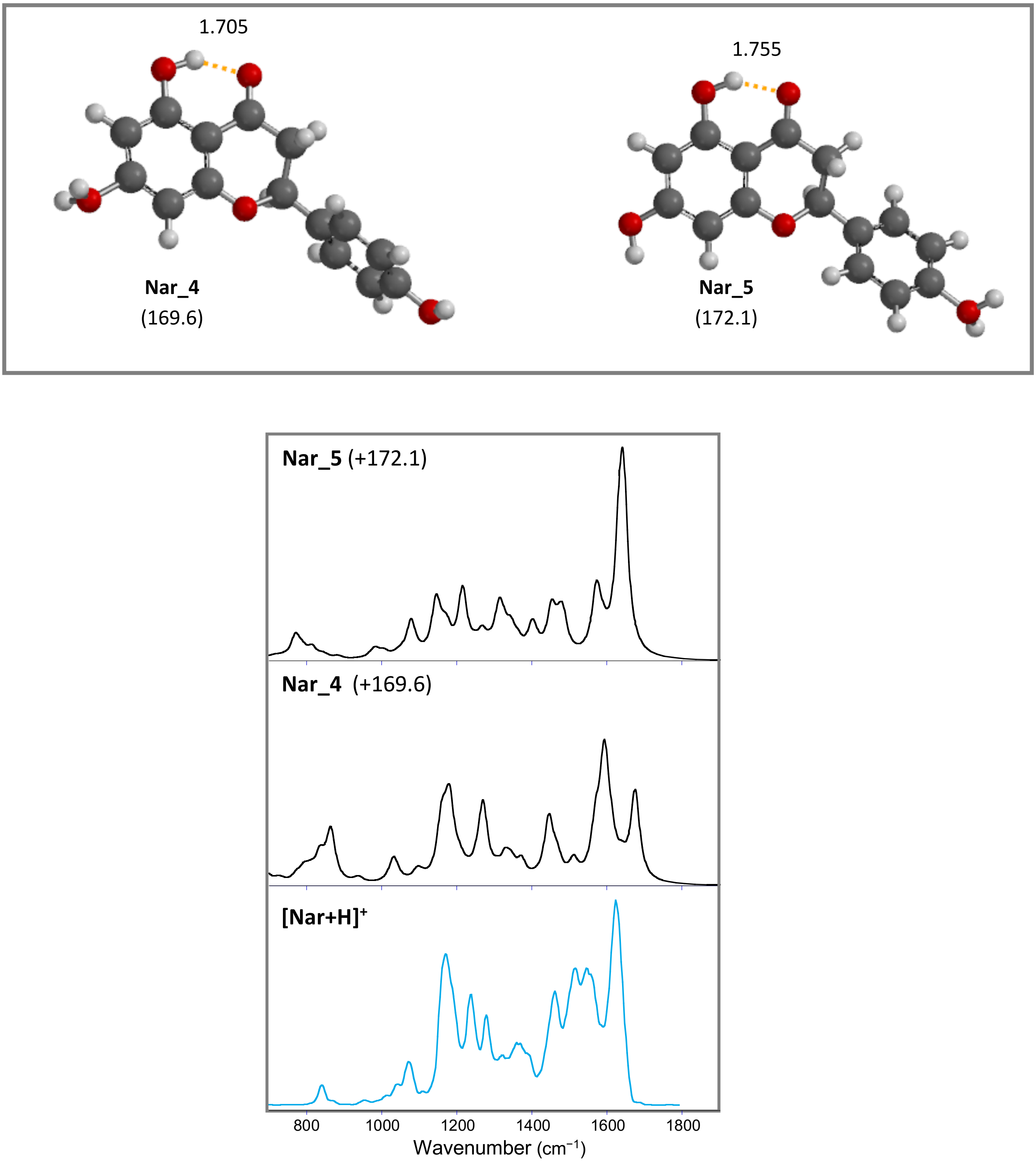

Figure S9. Experimental IRMPD spectrum of [[Nar+H]$^+$ (light blue profile) compared with the IR spectra calculated for representative isomers of naringenin protonated on 7-OH (**Nar_4**) or 4’-OH (**Nar_5**) position, whose optimized geometries are shown on the upper panel. Optimized geometries and IR calculated spectra are computed at the B3LYP/6-311++G(d,p) level of theory. The relative free energies (kJ mol^{-1}) with respect to the global minimum **Nar_1** are reported in parenthesis. Distances are given in Å. Calculated IR intensities (y-axis) are reported in the same scale (0-1000 Km mol^{-1})

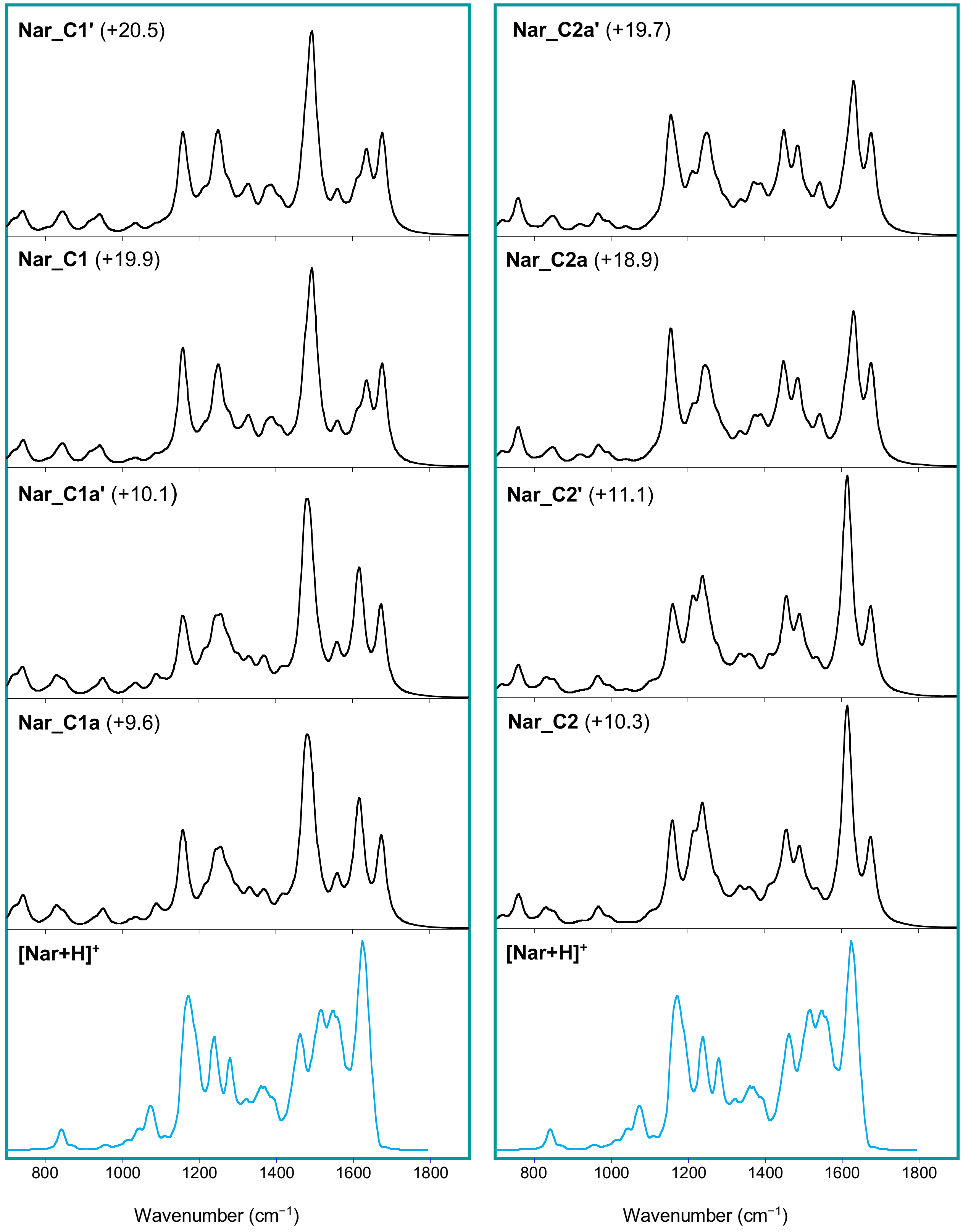

Figure S10. IRMPD spectrum (light blue profile) compared to the calculated IR spectra (black profiles) of the four lowest lying conformers of protonated naringenin, protonated in C8 (**Nar_C1a**, **Nar_C1'**, **Nar_C1** and **Nar_C1'**, left panel), and in C6 position (**Nar_C2**, **Nar_C2'**, **Nar_C2a** and **Nar_C2a'**, right panel). Free energies relative to **Nar_1**, are reported in parenthesis (kJ mol^{-1}). Calculated IR intensities (y-axis) are plotted in the same scale (0-1200 Km mol^{-1})

.

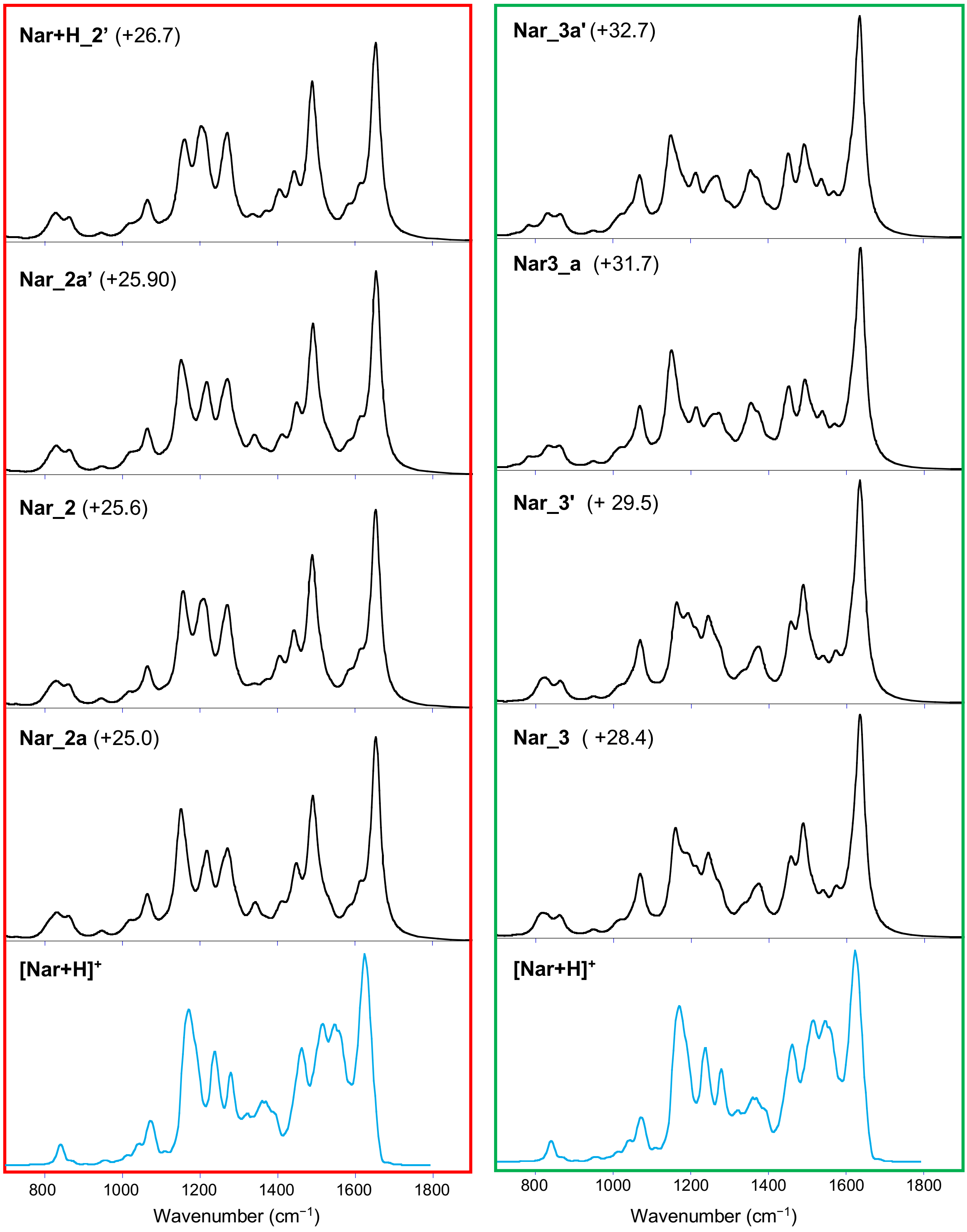

Figure S11. IRMPD spectrum (light blue profile) compared to the calculated IR spectra (black profiles) of the four lowest lying conformers of protonated naringenin, with clockwise orientation of both hydrogen atoms in 5OH and 4OH position (**Nar_2a**, **Nar_2**, **Nar_2a'** and **Nar_2'**, red panel), and without H-bonding motif between 5OH and 4OH (**Nar_3**, **Nar_3'**, **Nar_3a** and **Nar_3a'**, green panel). Free energies relative to **Nar_1**, are reported in parenthesis (kJ mol^{-1}). Calculated IR intensities (y-axis) are plotted in the same scale (0-1200 Km mol^{-1})

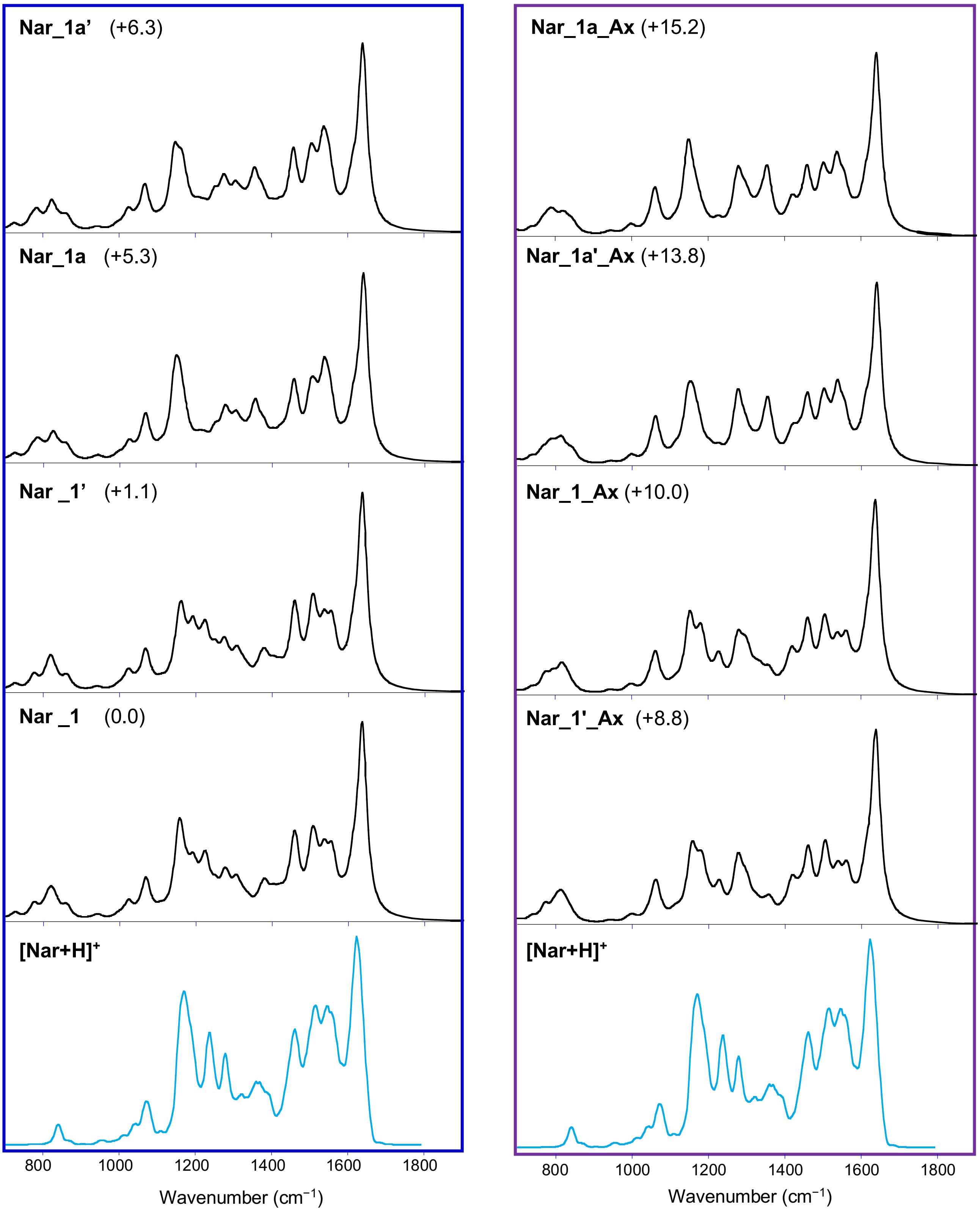

Figure S12. IRMPD spectrum (light blue profile) compared to the calculated IR spectra (black profiles) of the four lowest lying conformers of protonated naringenin, belonging to the ***Nar_1*** family that carry the B-ring in 1) equatorial position (**Nar_1**, **Nar_1’**, **Nar_1a** and **Nar_1a’**,left, blue panel), or 2) in axial position (**Nar_1’_Ax**, **Nar_1_Ax**, **Nar_1a’_Ax** and **Nar_1a’_Ax**, right scarlet panel). Free energies relative to **Nar_1**, are reported in parenthesis (kJ mol^{-1}). Calculated IR intensities (y-axis) are plotted in the same scale (0-1200 Km mol^{-1}).

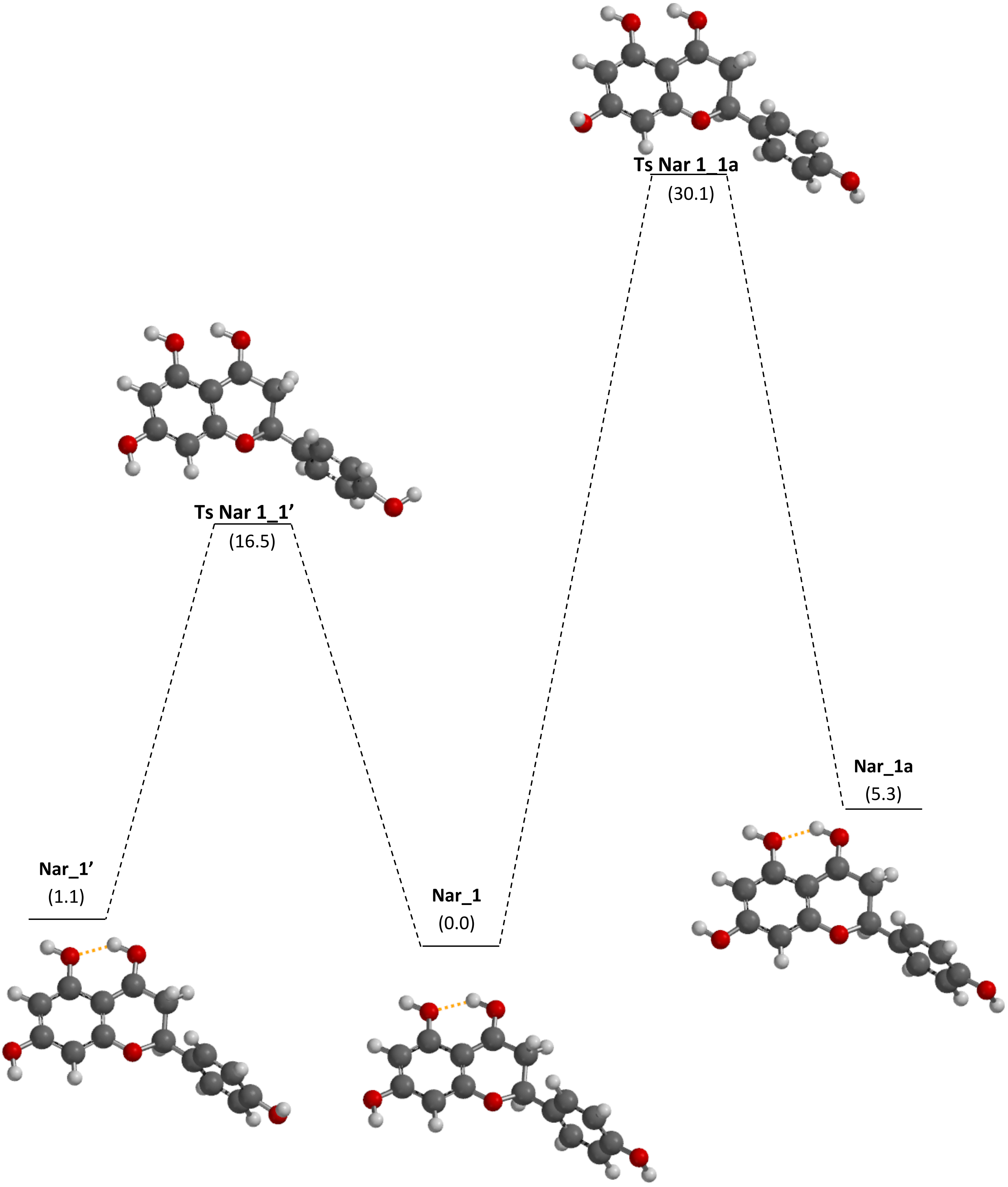

Figure S13. PES for the isomerization paths: **Nar_1'** ⟷ **Nar_1** ⟷ **Nar_1a** .
Geometries of the optimized structures and the transition state are computed at the B3LYP/6-311++G(d,p) level. Relative free energies (in parenthesis) are given in kJ mol^{-1}.

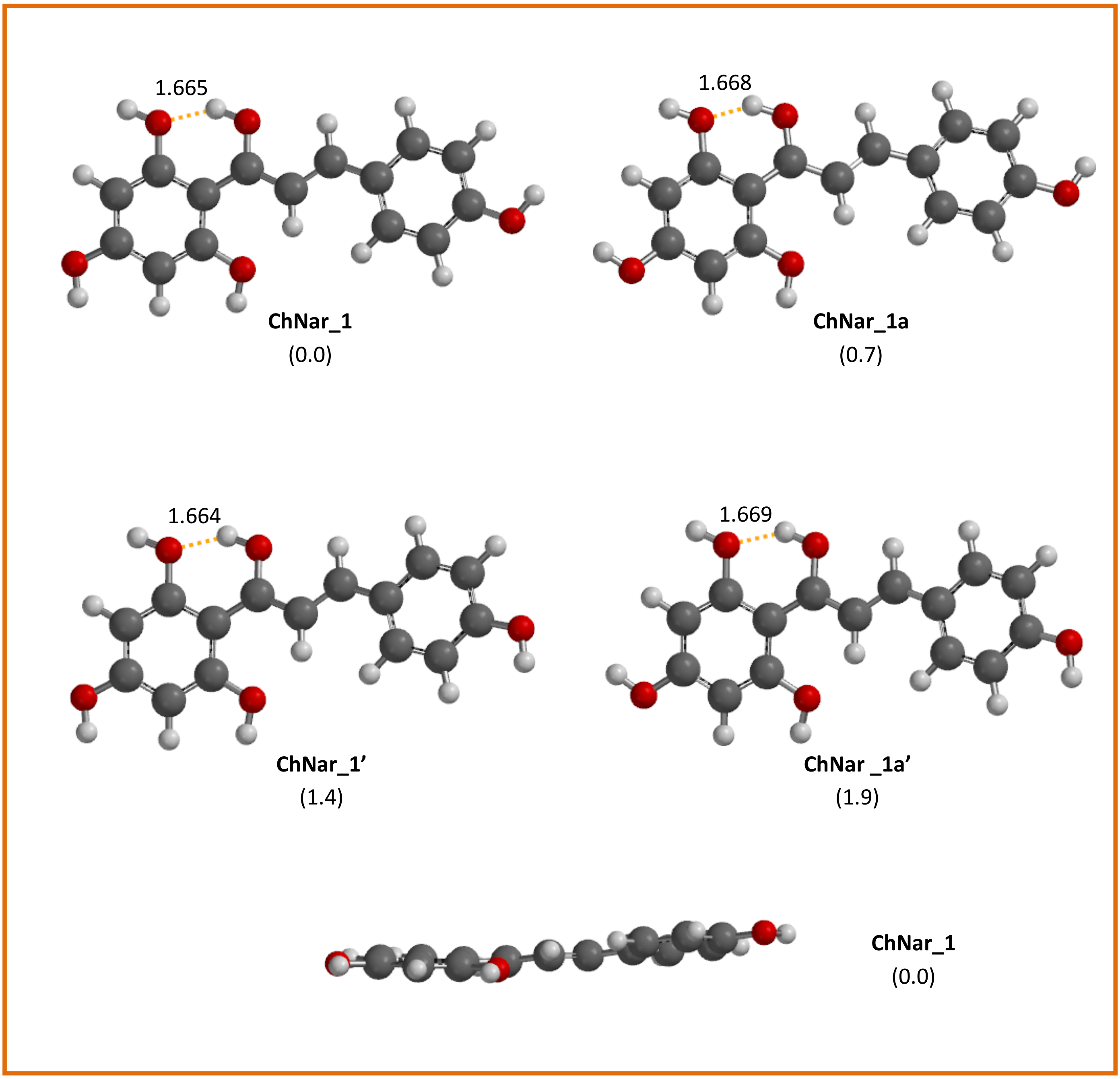

Figure S14. Optimized geometries and relative free energies (kJ mol^{-1}, in parenthesis) of conformers in *s-cis* configuration, belonging to the **Ch*Nar_1*** family, calculated at the B3LYP/6-311++G(d,p) level of theory. The relative free energies are calculated with respect to the global minimum **ChNar_1.** Distances are given in Å.

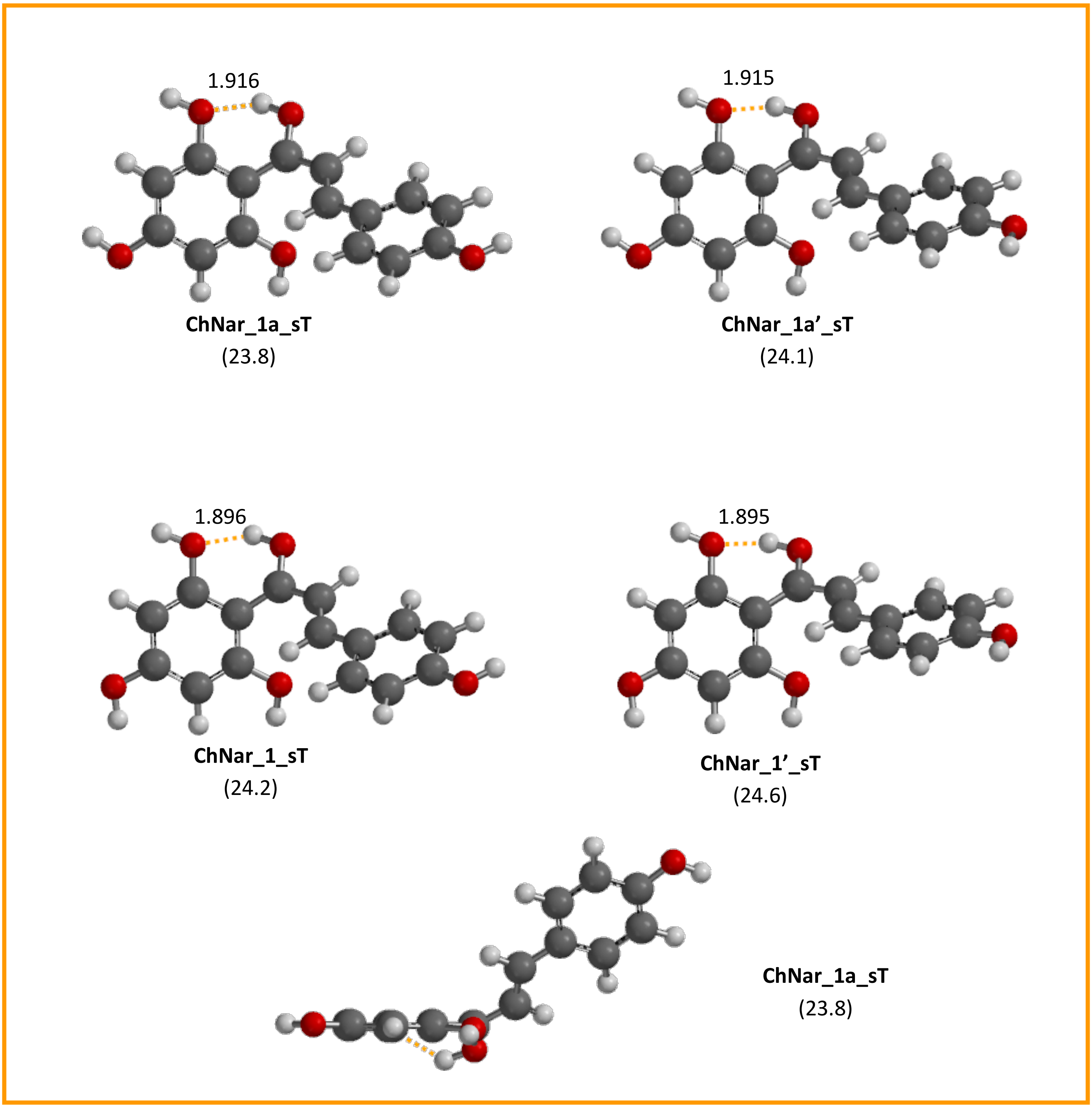

Figure S15. Optimized geometries and relative free energies (kJ mol^{-1}, in parenthesis) of conformers in *s-trans* configuration, belonging to the **Ch*Nar_1*** family, calculated at the B3LYP/6-311++G(d,p) level of theory. The relative free energies are calculated with respect to the global minimum **ChNar_1.** Distances are given in Å.

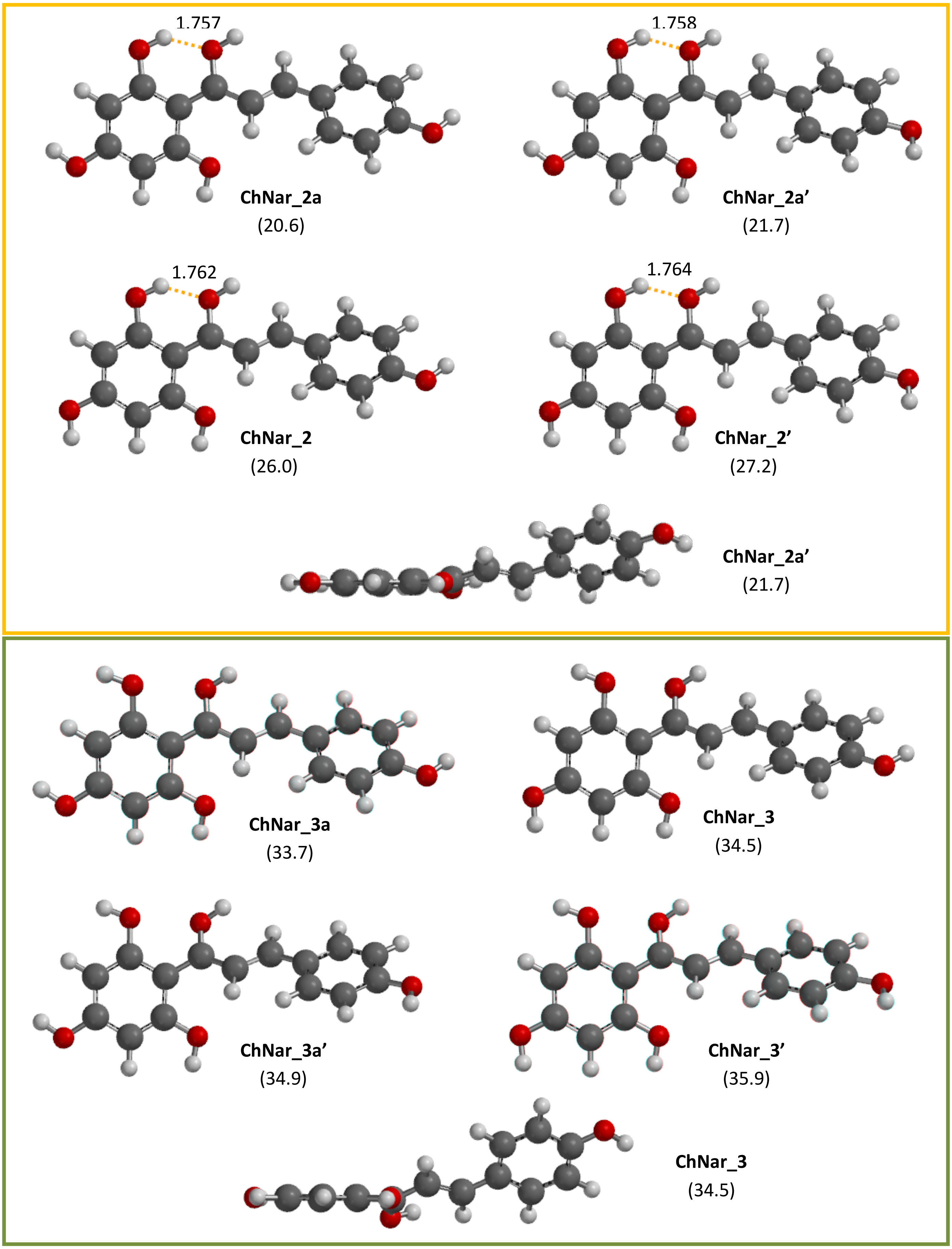

Figure S16. Optimized geometries and relative free energies (kJ mol^{-1}, in parenthesis) of conformers in *s_cis* configuration, belonging to the ***ChNar_2*** (upper yellow panel) and ***ChNar_3*** (lower, green panel) families, calculated at the B3LYP/6-311++G(d,p) level of theory. The relative free energies are calculated with respect to the global minimum **ChNar_1.** Distances are given in Å.

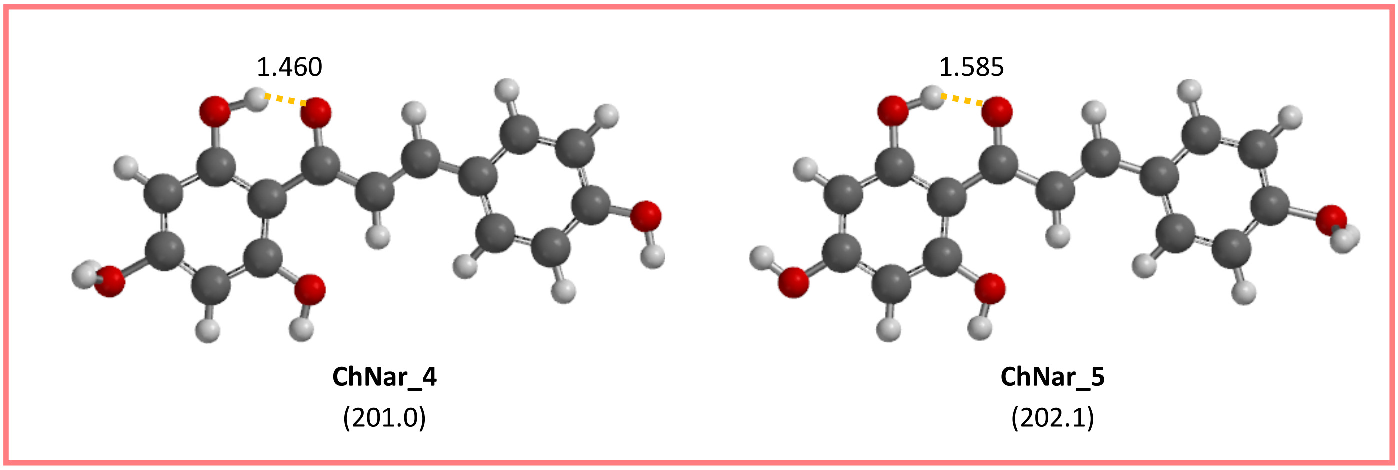

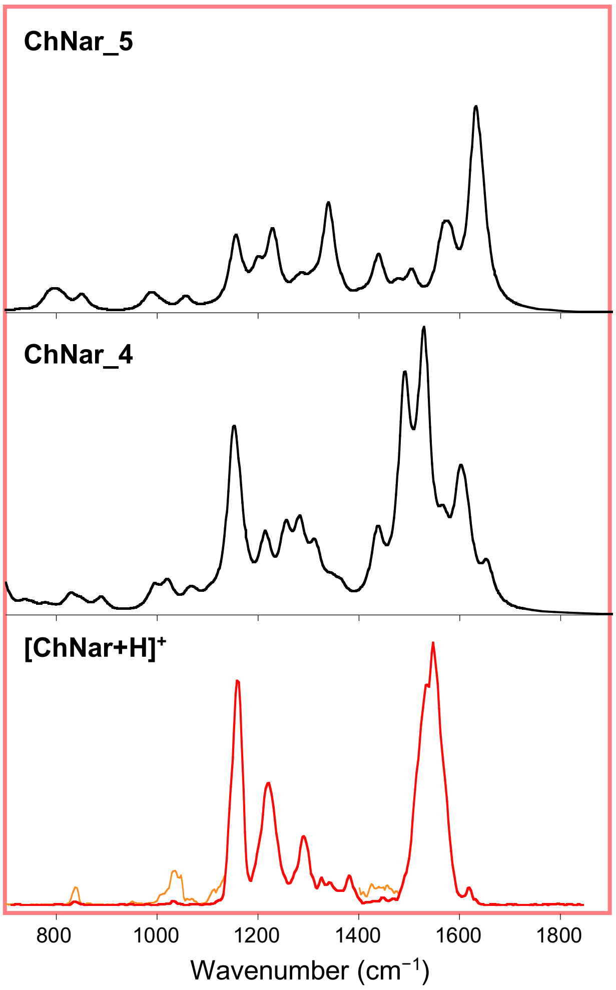

Figure S17. Experimental IRMPD spectrum of $[ChNar+H]^+$ (red profile) compared to the calculated IR spectra (black profiles) of representative isomers of naringenin chalcone protonated on 4’-OH (**ChNar_5**) or 4-OH (**ChNar_6**) position, whose optimized geometries are shown on the upper panel. Optimized geometries and IR calculated spectra are computed at the B3LYP/6-311++G(d,p) level of theory. The relative free energies (kJ mol^{-1}) with respect to the global minimum **ChNar_1** are reported in parenthesis. Distances are given in Å. Calculated IR intensities (y-axis) are reported in the same scale (0-1500 Km mol^{-1})

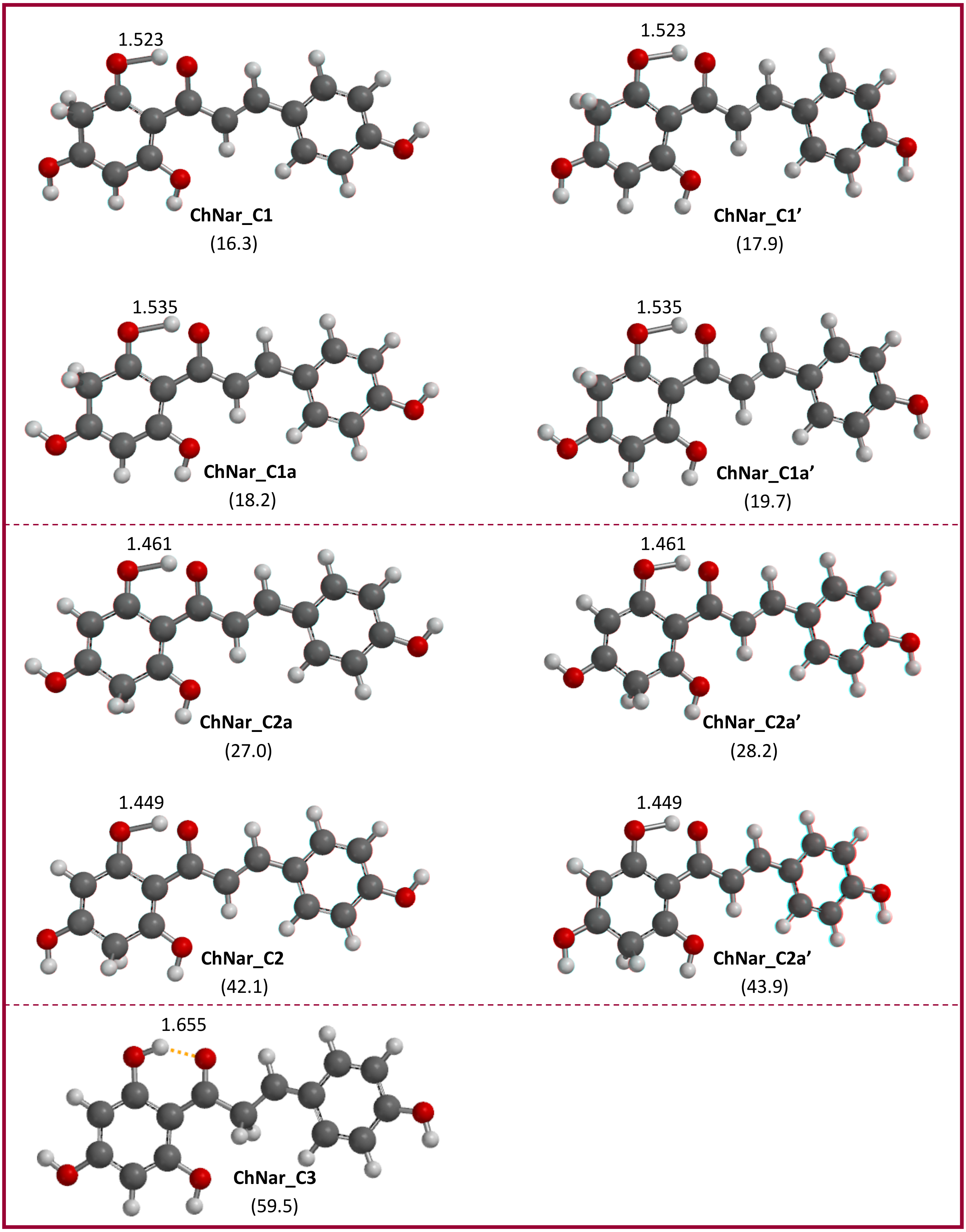

Figure S18. Optimized geometries and relative free energies (kJ mol^{-1}, in parenthesis) of conformers, belonging to the ***ChNar_C1*** (upper panel, protonated in C3’) and **Ch*Nar_C2*** (lower panel, protonated in C5’) families, and a representative conformer protonated in C$_\alpha$ **ChNar_C3** calculated at the B3LYP/6-311++G(d,p) level of theory. The relative free energies are calculated with respect to the global minimum **ChNar_1.** Distances are given in Å.

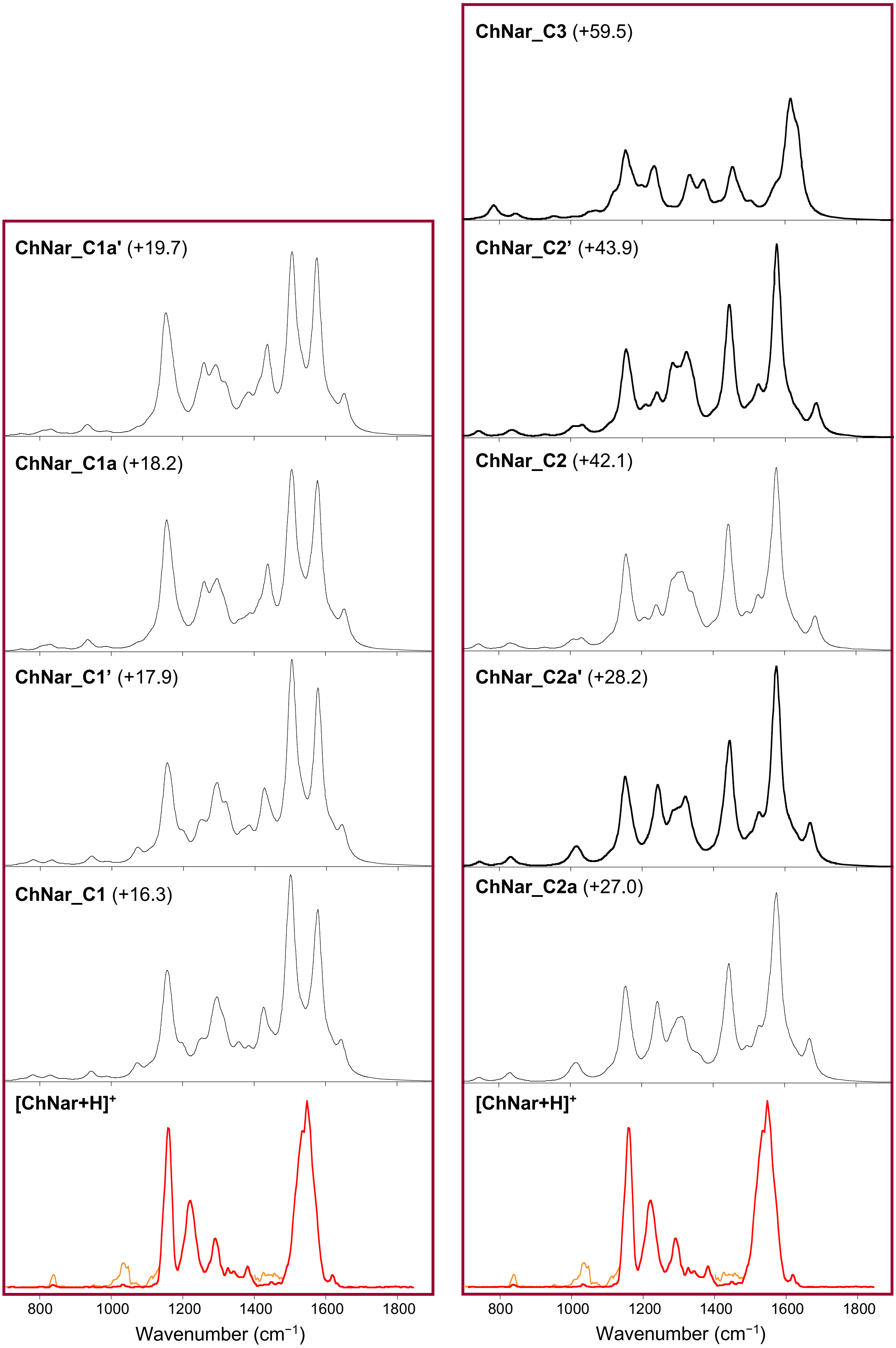

Figure S19. IRMPD spectrum (red profile) compared to the calculated IR spectra (black profiles) isomer of naringenin chalcone protonated in C3', in C5' or in Cα position. Free energies relative to **ChNar_1**, are reported in parenthesis (kJ mol^{-1}). Calculated IR intensities (y-axis) are plotted in the same scale (0-2500 Km mol^{-1})

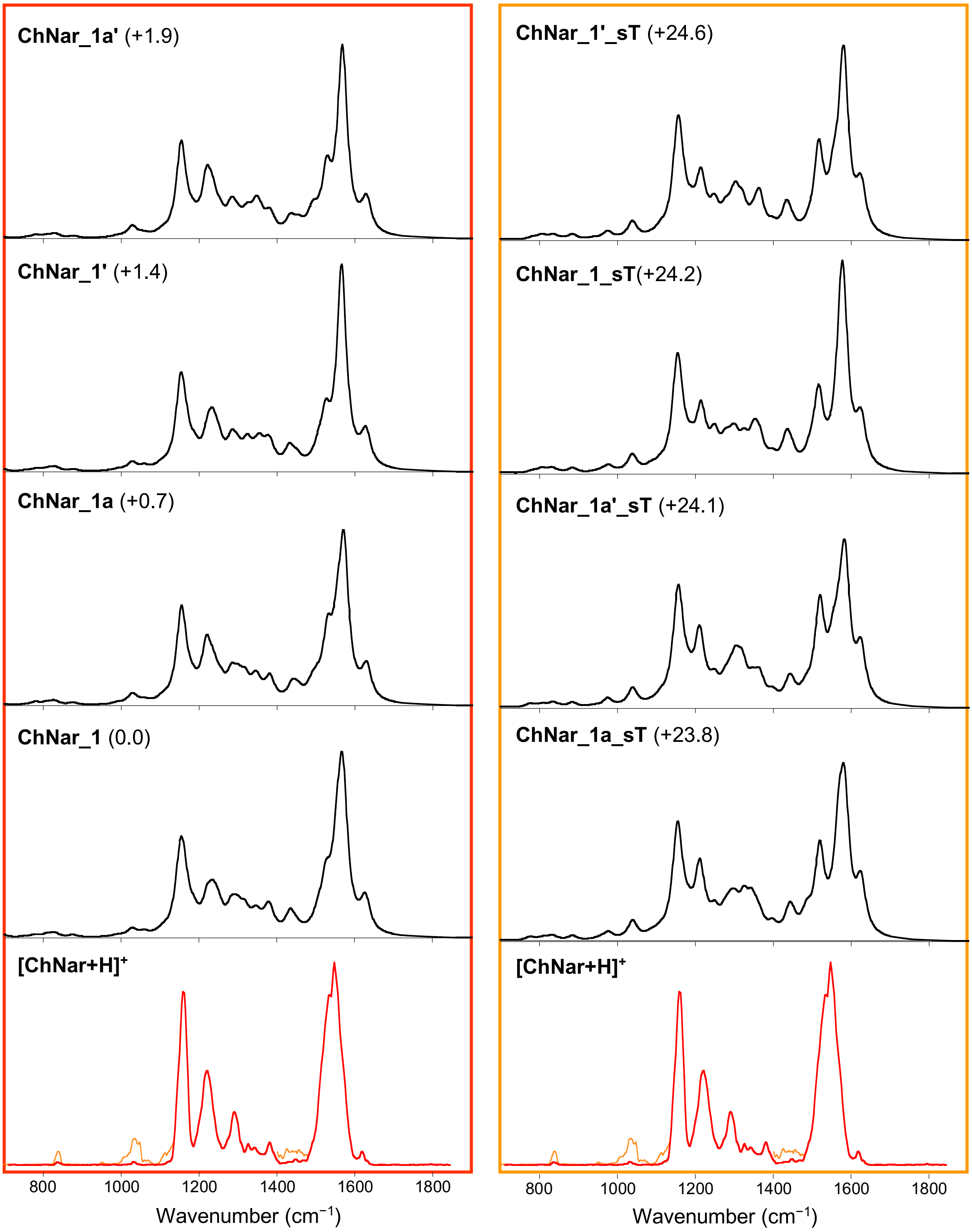

Figure S20. IRMPD spectrum (orange profile) compared to the calculated IR spectra (black profiles) of the four lowest lying conformers of protonated naringenin chalcone, belonging to the **Ch*Nar_1*** family in 1) *s-cis* configuration (left, orange panel), or 2) in *s-trans* configuration (right, yellow panel) whose geometries are shown in Figure 6 and S17-18. Free energies relative to **ChNar_1**, are reported in parenthesis (kJ mol^{-1}). Calculated IR intensities (Y axis) are plotted in the scale: 0-3500 and 0-2500 Km mol^{-1} for the *s-cis* and *s-trans* conformers, respectively.

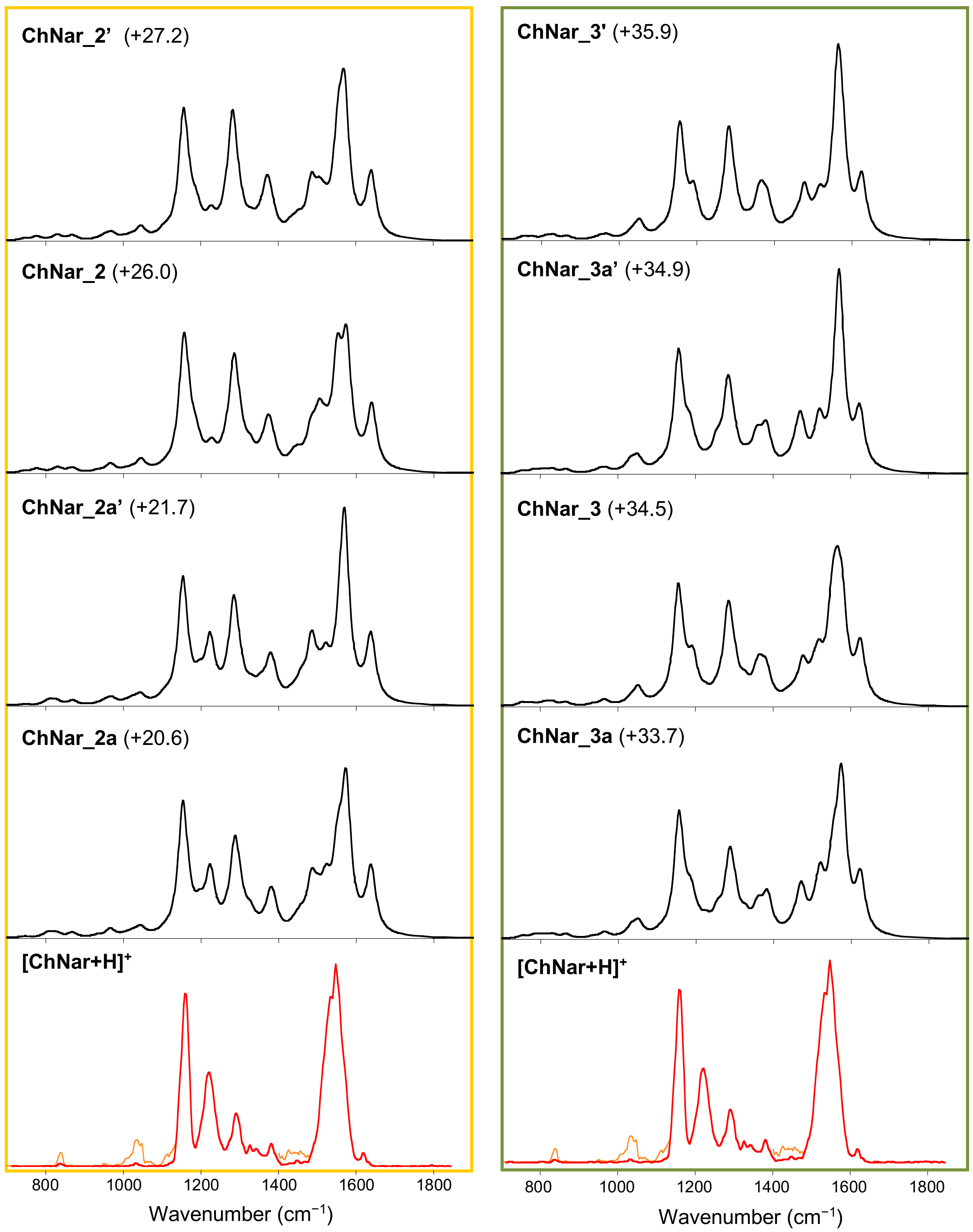

Figure S21. IRMPD spectrum (orange profile) compared to the calculated IR spectra (black profiles) of the four lowest lying conformers of protonated naringenin chalcone belonging to 1) the **Ch*Nar_2*** family, (left, yellow panel), and 2) to the **Ch*Nar_3*** family (right, green panel). Free energies relative to **Nar_1**, are reported in parenthesis (kJ mol^{-1}). Calculated IR intensities (Y axis) are plotted in the same scale (0-3000 Km mol^{-1})

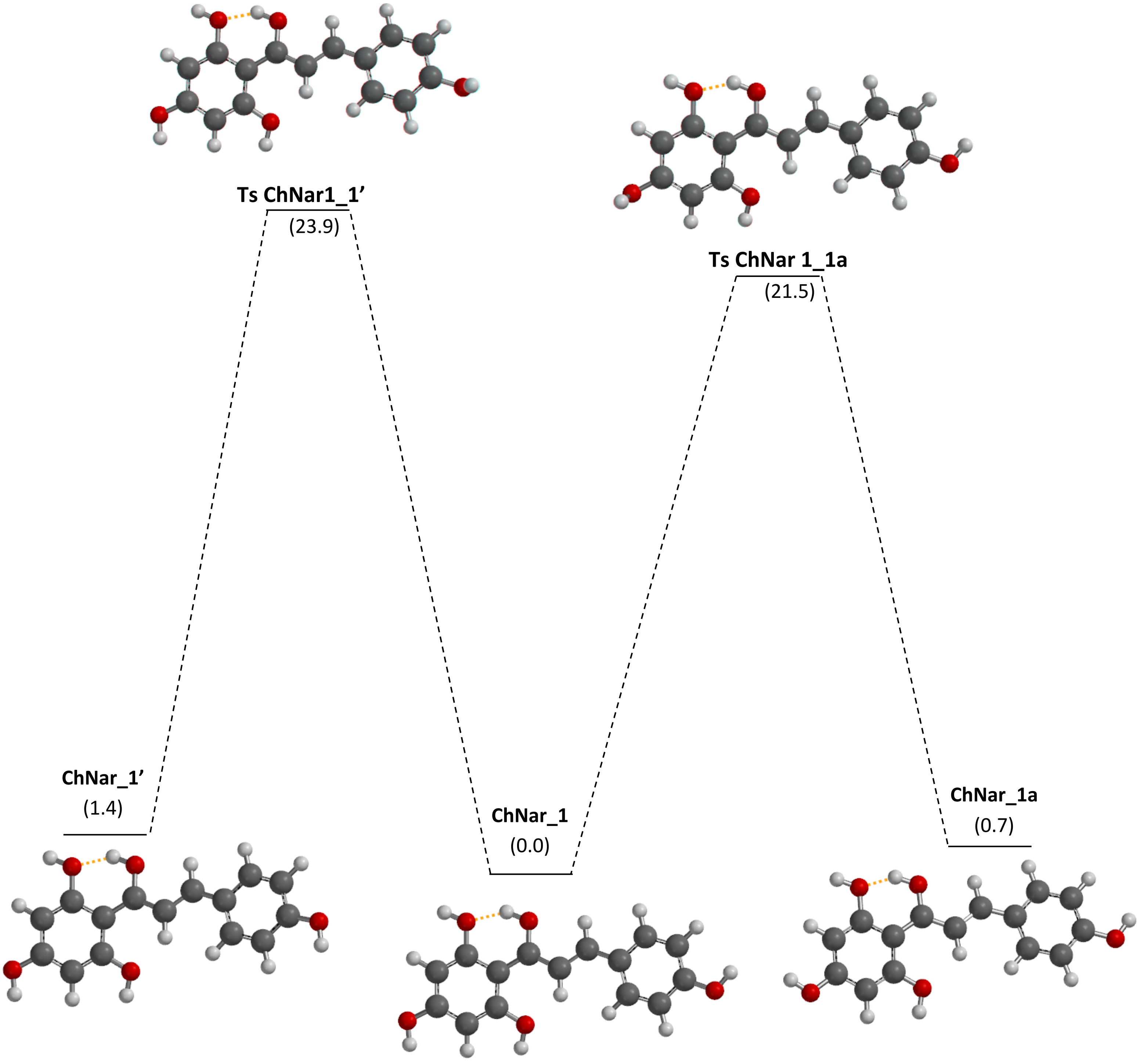

Figure S22. PES for the isomerization paths: **ChNar_1’** ⟷ **ChNar_1** ⟷ **ChNar_1a**. Geometries of the optimized structures and the transition state are computed at the B3LYP/6-311++G(d,p) level. Relative free energies (in parenthesis) are given in kJ mol^{-1}.

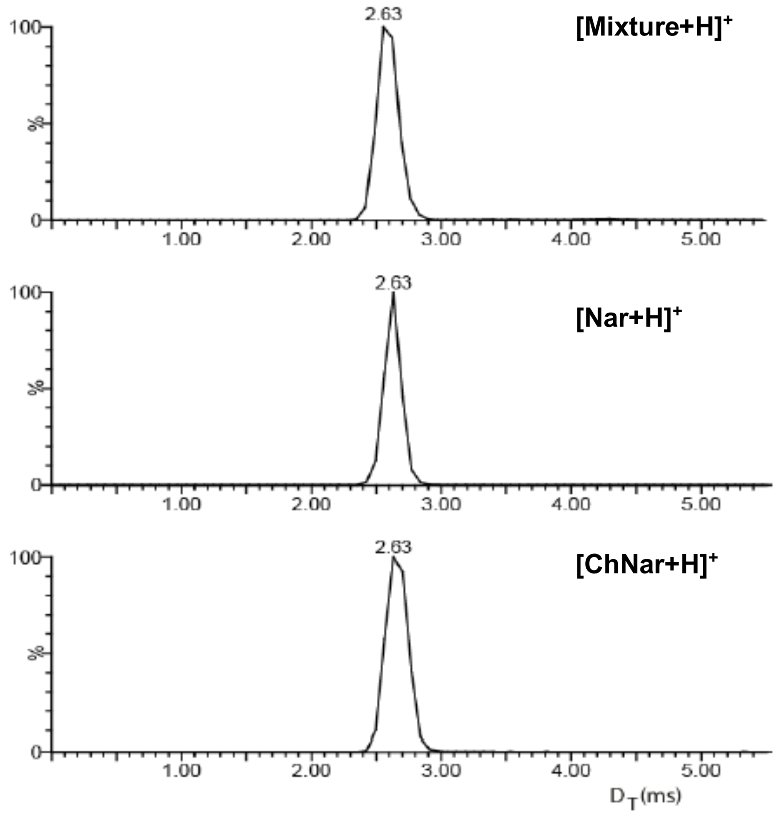

Figure S23. Extracted mobilogram of different protonated species observed at m/z 273

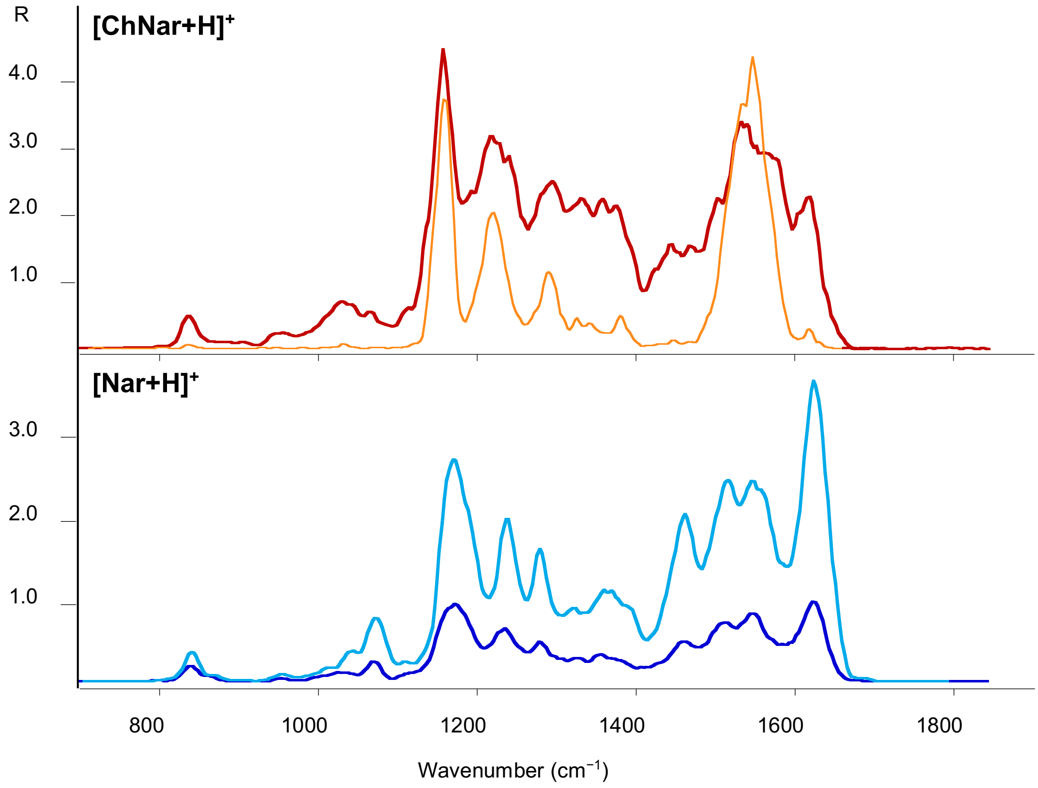

Figure S24. Comparison between the IRMPD spectra of mass-selected ions at m/z 273 from a) standard solution of naringenin recorded at higher and lower laser energy in light blue and blue trace respectively b) standard solution of naringenin chalcone recorded at higher and lower laser energy in red and orange trace respectively

Operating details

Tomato and grapefruit sample preparation

Fresh red grapefruits (Star Ruby, *Citrus Paradisi*) were washed and the albedos (white part of peel) were separated from the flavedos (orange part of peel). Fresh albedos were cut in small pieces for a hot methanol extraction. In a flask, 33 mL of methanol was added to 5.00 g of fresh albedos. The mixture was heated at 60°C for 3 hours and constantly stirred with a magnetic mixer. Then, 1 µL of extraction solution was diluted with 1 mL methanol.

Tomato skin was obtained from "datterino" peel, a variety of cherry tomatoes (*Solanum lycopersicum* L.). Tomato extraction was prepared using 1.57 g of tomato skin treated with 9 mL methanol and heated at 60°C for 3 hours while constantly stirred with a magnetic mixer. Finally, 1 µL of extraction solution was diluted with 1 mL methanol.

After the extraction, the diluted solutions were directly submitted to ESI in an ion trap mass spectrometer to verify the presence of the ion at m/z 273 in the natural extracts.

CID experiments

CID experiments at variable energy were performed in a hybrid triple quadrupole linear ion trap mass spectrometer ($Q1q2Q_{LIT}$) (2000 Q-Trap -Applied Biosystems). Typical ESI conditions were a flow rate of 10 µL min^{-1}, declustering potential set to 80V, and entrance potential of 4 V. The ions of interest were first mass-selected in Q1, then collided with N_2 (at a nominal pressure of 2.8 × 10^{-5} mbar) in the quadrupole collision cell q2 at varying collision energies (E_{lab} = 4- 45 eV) and finally the so-obtained dissociation product patterns were monitored by scanning Q_{LIT} in normal mode operation. The breakdown curves are acquired by plotting the relative abundances of fragment ions as a function of the collision energy, converted to the center of mass frame ($E_{CM} = [m/(m + M)]E_{lab}$, in which m and M are the masses of the collision gas and of the ion, respectively) and the values of phenomenological threshold energies (TEs) of the various fragment ions may be obtained from linear extrapolation of the rise of these sigmoid breakdown curves to the baseline.

Ion mobility experiments

Typical ESI conditions of the Synapt G2 HDMS quadrupole/time-of-flight mass spectrometer, were a 10 µL min^{-1} flow rate (capillary voltage 2.27 kV, sampling cone voltage 20 V) and curtain gas (N_2) flow of 100 L h^{-1} at 35° C. Accurate mass experiments were performed using reference ions from CH_3COONa external standard via LockSpray interface. IMS experiments were recorded using wave velocity of 600 m s^{-1}, wave height of 40V and helium cell gas flow of 180 mL min^{-1}. All data analyses were carried out using the MassLynx4.1 and DriftScope 2.1 programs provided by Waters.

IRMPD experiments

Positively charged ions generated by ESI were mass-isolated in the cell of an Amazon Speed ETD mass spectrometer prior to irradiation with 1 macropulse from FELIX operated at a repetition rate of 10 Hz. Each pulse has an energy of 20 to 160 mJ and bandwidth of 0.5% of the center frequency. The IRMPD spectrum is obtained by plotting the photofragmentation yield R ($R = -\ln[1 - \sum I(\text{fragment ions})/\sum I(\text{all ions})]$) as a function of the wavenumber of the IR radiation. A linear correction is applied for variations in the laser power, and the wavelength is calibrated using a grating spectrometer.

Computational Details

The lowest energy conformations of candidate isomers of the sampled $[Nar+H]^+$ and $[ChNar+H]^+$ ions are obtained adopting two different strategies. Preliminary conformational searches were performed for each protonation site using the Conformer Distribution tool, together with MMFF molecular mechanics force field. Conformers were subsequently optimized using density functional theory (DFT) calculations at the B3LYP/6-311++G(d,p) level. Furthermore, planar geometries for each isomer, diversified by the orientation of 7-OH and 4'-OH for Nar (or 4'-OH and 4-OH in the case of ChNar) with torsion angles H-O-C7-C8 and H-O-C4'-C3' (or H-O-C4'-C3' and H-O-C4-C3) of 0° and 180° degrees were taken as the starting point for DFT optimizations. In the case of Naringenin, DFT calculations at the B3LYP/6-311++G(d,p) level of theory were performed for S-enantiomer geometries. For ChNAr, geometry optimizations were achieved starting from (2-E)-naringenin chalcone, (with the α,β-double bond in *trans* configuration). The IR frequencies were scaled by 0.975 as an approximate correction for anharmonicity and convoluted with a Lorentzian shape with a full width at half maximum of 15 cm^{-1} to simulate the experimental spectrum.

Table S1. Phenomenological threshold energies (TEs) for the energy resolved CID experiments of mass-selected $[Nar+H]^+$ and $[ChNar+H]^+$ (*m/z* 273) ions.

Fragment ion (m/z)	TE (eV) of $[Nar+H]^+$	TE (eV) of $[ChNar+H]^+$
153	1.00	1.15
147	0.70	1.00
119	2.30	2.50
91	3.30	3.60

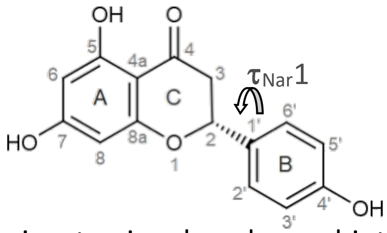

Table S2. Relative free energies, torsional angles and intramolecular H-bond length values of the isomers and conformers of protonated naringenin optimized in gas phase at B3LYP/6-311++G(d,p) level of theory.

A B

Structure	ΔG (kJ mol⁻¹)	τ_{Nar}1[a]	τ_{Nar}2[b]	τ_{Nar}3[c]	H-bond length (Å)
**Nar_1**	0.0	56.83°	−179.80°	179.91°	1.791 (d)
**Nar_1'**	1.1	56.79°	−179.89°	0.27°	1.792 (d)
**Nar_1a**	5.3	57.26°	0.35°	179.68°	1.796 (d)
**Nar_1a'**	6.3	57.47°	0.50°	0.49°	1.796 (d)
**Ts Nar 1_1'**	16.50	59.71°	−179.81°	-92.54	1.783 (d)
**Ts Nar 1-1a**	30.1	55.67°	−90.46	179.68°	1.792 (d)
**Nar_1'_Ax**	8.8	−172.02°	−179,95°	1.31°	1.786 (d)
**Nar_1_Ax**	10.0	−174.71°	−179,90°	178.02°	1.786 (d)
**Nar_1a'_Ax**	13.8	−174.05°	−0.07°	0.98°	1.791 (d)
**Nar_1a_Ax**	15.2	−176.30°	−0.11°	177.88°	1.790 (d)
**Nar_C1a**	9.6	50.31°	−0.35°	−179.57°	1.652 (e)
**Nar_C1a'**	10.1	49.13°	−0.30°	0.03°	1.651(e)
**Nar_C1**	19.9	50.18°	179.55°	−179.35°	1.661 (e)
**Nar_C1'**	20.5	48.81°	179.57°	−0.08°	1.661 (e)
**Nar_C2**	10.3	51.31°	179.88°	−179.54°	1.644 (e)
**Nar_C2'**	11.10	50.66°	179.89°	0.02°	1.645 (e)
**Nar_C2a**	18.9	51.41°	−0.08°	−179.82°	1.641 (e)
**Nar_C2a'**	19.7	51.04°	−0.32°	0.25°	1.641(e)
**Nar_2a**	25.0	59.08°	0.34°	179.00°	1.878 (e)
**Nar_2**	25.6	58.35°	−179.72°	179.26°	1.883 (e)
**Nar_2a'**	25.9	60.27°	0.45°	0.87°	1.878 (e)
**Nar_2'**	26.7	59.76°	−179.81°	0.65°	1.883 (e)
**Nar_3**	28.4	58.14°	−179.96°	179.39°	-
**Nar_3'**	29.5	59.14°	179.95 °	0.56°	-
**Nar_3a**	31.7	58.45°	0.05°	179.17°	-
**Nar_3a'**	32.7	59.53°	0.18°	0.71°	-
**Nar_4**	169.6	55.54°	-	−0.40°	1.705 (d)
**Nar_5**	172.1	103.33°	−179.83°	-	1.755 (d)

a) dihedral angle formed by C3-C2-C1'-C2' atoms
b) dihedral angle formed by C6-C7-O(7)-H(7) atoms
c) dihedral angle formed by C3'-C4'-O(4')-H(4') atoms
d) between OH(4) and O(5)
e) between OH(5) and O(4)

Table S3. CO bond length values of the isomers and conformers of protonated naringenin optimized in gas phase at B3LYP/6-311++G(d,p) level of theory (the computed IR position for C-O vibrational stretching mode are in parenthesis)

**Structure**	**C4-O** length (Å)	**C5-O lenght** (Å)	**C7-O lenght** (Å)	**C4'-O lenght** (Å)
**Nar_1**	1.305 (1504 cm^{-1})	1.360 (1225 cm^{-1})	1.332 (1459 cm^{-1})	1.355 (1275 cm^{-1})
**Nar_1'**	1.305	1.360	1.332	1.355
**Nar_1a**	1.305	1.360	1.333	1.355
**Nar_1a'**	1.305	1.360	1.333	1.355
**Ts Nar 1_1'**	1.304	1.360	1.332	1.377
**Ts Nar 1-1a**	1.301	1.361	1.358	1.354
**Nar_1'_Ax**	1.306 (1503 cm^{-1})	1.361 (1227 cm^{-1})	1.333 (1460 cm^{-1})	1.354 (1275 cm^{-1})
**Nar_1_Ax**	1.306	1.361	1.333	1.354
**Nar_1a'_Ax**	1.306	1.361	1.334	1.355
**Nar_1°_Ax**	1.306	1.361	1.333	1.355
**Nar_C1a**	1.228 (1675 cm^{-1})	1.298 (1476 cm^{-1})	1.325 (1423 cm–$^{-1}$)	1.353 (1279 cm^{-1})
**Nar_C1a'**	1.228	1.298	1.325	1.353
**Nar_C1**	1.227	1.298	1.327	1.353
**Nar_C1'**	1.227	1.298	1.327	1.353
**Nar_C2**	1.230 (1675 cm^{-1})	1.296 (1490 cm^{-1})	1.325 (1456 cm^{-1})	1.353 (1279 cm^{-1})
**Nar_C2'**	1.230	1.296	1.325	1.353
**Nar_C2a**	1.230	1.297	1.327	1.353
**Nar_C2a'**	1.230	1.297	1.237	1.353
**Nar_2a**	1.335 (1344 and 1490 cm^{-1})	1.332 (1490 cm^{-1})	1.329 (1448 cm^{-1})	1.354 (1275 cm^{-1})
**Nar_2**	1.334	1.333	1.333	1.354
**Nar_2a'**	1.335	1.332	1.329	1.355
**Nar_2'**	1.334	1.333	1.330	1.354
**Nar_3**	1.314 (1488 and (1357 cm^{-1})	1.337 (1488 cm^{-1})	1.332 (1455 cm^{-1})	1.355 (1275 cm^{-1})
**Nar_3'**	1.314	1.337	1.332	1.355
**Nar_3a**	1.314	1.336	1.332	1.355
**Nar_3a'**	1.314	1.337	1.332	1.355
**Nar_4**	1.227 (1675 cm^{-1})	1.322 (1446 and 1361 cm^{-1})	1.512 (865 cm^{-1})	1.357 (1269 cm^{-1})
**Nar_5**	1.233 (1664 and 1633 cm^{-1})	1.333 (1452 and 1313 cm^{-1})	1.352 (1483 and 1313 cm^{-1})	1.507 (735 cm^{-1})

Table S4. Computational data associated with the various structures considered.

Structure	E (Hartree)	ZPE (kJ mol^{-1})	Enthalpy Corr. 298 K (kJ mol^{-1})	H-TS 298 K (kJ mol^{-1})	Relative ΔH (298 K) (kJ mol^{-1})	Relative ΔG 298 K (kJ mol^{-1})
**Nar_1**	-955.581919	673.4087	713.6921	568.0628	0.0	0.0
**Nar_1’**	-955.581661	673.7368	713.9211	568.4712	0.9	1.1
**Nar_1a**	-955.579708	672.9764	713.4047	567.5268	5.5	5.3
**Nar_1a’**	-955.579462	673.2714	713.6096	567.8944	6.4	6.3
**Nar_1’_Ax**	-955.579250	674.3070	714.4814	569.8682	7.8	8.8
**Nar_1_Ax**	-955.579039	674.7843	714.8398	570.4631	8.7	10.0
**Nar_1a’_Ax**	-955.577219	674.0807	714.3812	569.5808	13.0	13.9
**Nar_1a_Ax**	-955.576862	674.4208	714.6523	569.9891	14.2	15.2
**Nar_2a**	-955.572005	672.5388	713.0750	567.0291	25.4	25.0
**Nar_2**	-955.571749	672.7364	713.2129	567.2723	26.2	25.9
**Nar_2a’**	-955.571725	672.4159	713.0011	566.8721	26.1	25.6
**Nar_2’**	-955.571392	672.6352	713.1495	567.1474	27.1	26.7
**Nar_3**	-955.570734	672.6426	713.1950	567.0725	28.9	28.4
**Nar_3’**	-955.570405	672.8664	713.3495	567.3559	29.9	29.5
**Nar_3a**	-955.569310	672.3299	712.9661	566.7067	32.4	31.7
**Nar_3a’**	-955.569060	672.5814	713.1452	567.0176	33.2	32.7
**Nar_4**	-955.514911	668.0051	708.8994	561.8114	171.1	169.6
**Nar_5**	-955.514335	669.0717	709.9908	562.7896	173.7	172.1
**Nar_C1a**	-955.577223	670.8016	710.5192	565.3585	9.2	9.6
**Nar_C1a’**	-955.576948	670.5764	710.3625	565.0773	9.7	10.1
**Nar_C1**	-955.572898	669.9862	710.0385	564.3196	20.0	19.9
**Nar_C1’**	-955.572570	669.7924	709.9049	564.0752	20.8	20.6
**Nar_C2**	-955.576723	670.1815	709.9753	564.7073	9.9	10.3
**Nar_C2’**	-955.576391	670.1483	709.9601	564.6562	10.8	11.1
**Nar_C2a**	-955.573031	669.3093	709.3883	563.6254	19.0	18.9
**Nar_C2a’**	-955.572714	669.3016	709.3850	563.6118	19.9	19.7
**Ts Nar 1_1’**	-955.574409	670.9346	711.4879	564.8655	17.5	16.5
**Ts Nar1_1a**	-955.568526	669.2640	710.1464	562.9841	31.6	30.1

Table S5. Positions of the IRMPD bands of protonated Naringenin, $[Nar+H]^+$, and computed vibrational modes for the lowest-energy **Nar_1**

Wavenumber (cm^{-1})		Vibrational mode[b]
IRMPD	Calculated[a]	
840	815 (53)	βC6H oop
	819 (53)	βC8H oop
	827 (59)	βC H oop (C2', C3' and C8)
867	861 (63)	σ CC breathing (B ring)
950	942 (23)	Rock CH2
1013-1040	1023 (78)	σ CC breathing (C ring)
1070	1069 (196)	σ C5-OH + σ O1-C8a + σ CC breathing (A ring)
1106	1107 (22)	β H ip (B ring)
1170	1152 (131)	β H ip (A ring)
	1157 (313)	β OH (C4')
	1168 (49)	β H ip (B ring)
	1177 (42)	Twist CH2 (C3) + β CH
	1193 (182)	σ C7-OH + β OH (C5) + Twist CH2 (C3) + β CH ip (A and C ring)
1240	1212 (52)	Twist CH2 (C3) + σ C3-C1'
	1225 (234)	σ C4-OH + β OH (C7) + σ C5-OH + β H ip (A ring)
	1250 (88)	Wag CH2 (C3) + β CH (C2)
1280	1275 (144)	σ C4'-OH + β CH ip (B ring)
	1285 (54)	β OH (C4 and C5) + β CH ip
1316	1306 (132)	β OH (C4) + β CH ip (C and B ring)
	1321 (46)	β CH (C2 and C3)
	1336 (42)	β OH (C4') + σ CC (B ring)
1356	**1355 (229)**[c]	σ CC (A and C ring)+ β H ip (A and C ring)
1390	1372 (53)	β CH (C3)
	1381 (104)	σ C4a–C8a + β CH (C2)
	1402 (69)	Sciss CH2 (C3)
1460	1420 (61)	β OH (C4) +β CH (A and C ring) + σ CC (A and C ring)
	1441 (37)	β CH ip (B ring) + β CH (C2) + σ CC (B ring)
	1459 (385)	σ C7-OH + β H ip (A ring) + σ CC (A ring) + σ C4-OH
1513	1504 (269)	σ C4-OH + β H ip (A ring) + σ CC (A ring)
	1512 (134)	σ CC (B ring)+ β C-H ip (B ring)
	1536 (218)	σ C4-C4a + σ CC (A ring)
1550	1557 (251)	σ C4-C4a + β OH ip (C4) + σ CC (A ring)
1623	1586 (33)	σ CC (B ring)
	1611 (160)	σ CC (B ring)
	1636 (980)	σ CC (A ring)

[a]Vibrational frequencies calculated at the B3LYP/6-311++G(d,p) level of theory are scaled by a factor of 0.975. The computed intensities (km mol^{-1}) are given in parenthesis. Bands with intensity lower than 20 km mol^{-1} are not reported.
[b] β = bending; σ = stretching; oop = out-of-plane; ip= in-plane; Rock= rocking; Twist = twisting; Wag = wagging; the numbering of the atoms follows Figure 1.
[c] this calculated IR feature belongs of **Nar_1a** conformer

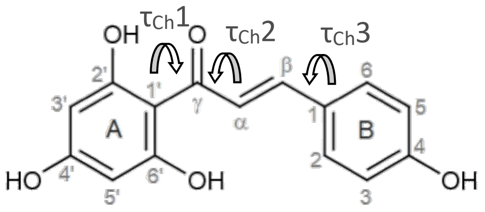

Table S6. Relative free energies, torsional angles and intramolecular H bond length values of the isomers and conformers of protonated naringenin chalcone optimized at B3LYP/6-311++G(d,p) level of theory.

**Structure**	**ΔG (kJ mol^{-1})**	**τ_{Ch}1^{a}**	**τ_{Ch}2^{b}**	**τ_{Ch}3^{c}**	**τ_{Ch}4^{d}**	**τ_{Ch}5^{e}**	H bond length (Å)
**ChNar_1**	0.0	−10.19°	−1.85°	0.18°	179.95°	179.87°	1.665 (f)
**ChNar_1a**	0.7	−9.96°	−1.51°	0.48°	−0.11°	179.85°	1.668 (f)
**ChNar_1’**	1.4	−10.28°	−1.67°	0.31°	179.87°	−0.23°	1.664 (f)
**ChNar_1a’**	1.9	−10.21°	−1.40°	0.59°	−0.13°	−0.19°	1.669 (f)
**ChNar_1a_sT**	23.8	−33.93°	160.57°	173.13°	−0.42°	−179.95°	1.916 (f)
**ChNar_1a’_sT**	24.1	−33.80°	160.57°	174.13°	−0.27°	0.04°	1.916 (f)
**ChNar_1_sT**	24.2	−33.21°	159.93°	174.00°	179.26°	−179.86°	1.896 (f)
**ChNar_1’_sT**	24.6	−33.08°	159.92°	174.00°	179.18°	−0.08°	1.895 (f)
**ChNar_2a**	20.6	−14.98°	−17.07°	-5.79°	−0.65°	−179.87°	1.757 (g)
**ChNar_2a’**	21.7	−15.09°	−17.22°	-6.45°	−0.53°	0.03°	1.758 (g)
**ChNar_2**	26.0	−15.35°	−17.35°	-6.03°	179.83°	−179.74°	1.762 (g)
**ChNar_2’**	27.2	−15.62°	−17.53°	-6.78°	179.72°	−0.02°	1.764 (g)
**ChNar_3a**	33.7	−27.90°	−15.76°	-6.22°	0.49°	179.76°	-
**ChNar_3**	34.5	−28.03°	−15.65°	-6.20°	−179.72°	179.78°	-
**ChNar_3a’**	34.9	−27.71°	−15.75°	-6.38°	0.62°	0.13°	-
**ChNar_3’**	35.9	−28.19°	−15.57°	-6.42°	−179.75°	0.04°	-
**ChNar_C1**	16.3	179.02	−1.14°	0.03°	−179.86°	−179.94°	1.523 (f)
**ChNar_C1’**	17.9	−0.73°	−1.00°	−1.31°	−179.89°	0.10°	1.523 (f)
**ChNar_C1a**	18.2	−0.78°	−1.06°	−1.30°	−0.30°	−179.93°	1.535 (f)
**ChNar_C1a’**	19.7	0.75°	−1.06°	−1.38°	0.02°	0.17°	1.535 (f)
**ChNar_C2a**	26.7	−0.70°	0.07°	0.83°	−0.29°	179.81°	1.461 (f)
**ChNar_C2a’**	28.2	−0.52°	0.22°	0.98	−0.23°	0.01°	1.461 (f)
**ChNar_C2**	42.1	2.32°	0.27°	0.67°	−179.44°	179.80°	1.449 (f)
**ChNar_C2’**	43.9	2.14°	0.24°	0.72°	−179.35°	0.05°	1.449 (f)
**ChNar_C3**	59.529	−0.95°	2.63°	0.03°	0.20°	0.14°	1.665 (g)
**ChNar_4**	201.049	−3.74°	−0.13°	1.72°	-	−0.26°	1.460 (g)
**ChNar_5**	202.129	1.24°	0.06°	3.76°	−0.11°	-	1.585 (d)

a) dihedral angle formed by C2’-C1’-Cγ-O(γ) atoms
b) dihedral angle formed by O(γ)-Cγ-Cα-Cβ atoms
c) dihedral angle formed by Cα-Cβ-C1-C2 atoms
d) dihedral angle formed by C3’-C4’-O(4’)-H(4’) atoms (A ring)
e) dihedral angle formed by C3-C4-O(4)-H(4) atoms (B ring)
f) between OH(γ) and O(2’)
g) between OH(2’) and O(γ)

Table S7. Computational data associated with the various structures considered.

Structure	E (Hartree)	ZPE (kJ mol^{-1})	Enthalpy Corr. 298 K (kJ mol^{-1})	H-TS 298 K (kJ mol^{-1})	Relative ΔH (298 K) (kJ mol^{-1})	Relative ΔG 298 K (kJ mol^{-1})
**ChNar_1**	-955.585352	668.6011	710.9572	560.8912	0.0	0.0
**ChNar_1a**	-955.585156	668.7693	711.1133	561.0557	0.7	0.7
**ChNar_1’**	-955.584777	668.4857	710.8517	560.7709	1.4	1.4
**ChNar_1a’**	-955.584644	668.6808	711.0217	560.9758	1.9	1.9
**ChNar_1a_sT**	-955.575909	667.4445	710.1533	559.9409	24.0	23.8
**ChNar_1a’_sT**	-955.575947	667.7455	710.3678	560.3038	24.1	24.1
**ChNar_1_sT**	-955.575939	667.7899	710.3619	560.4011	24.1	24.2
**ChNar_1’_sT**	-955.575939	668.0797	710.5654	560.7531	24.3	24.6
**ChNar_2a**	-955.577812	668.9689	710.9714	561.6867	19.8	20.6
**ChNar_2a’**	-955.577429	669.018	710.9972	561.7559	20.8	21.7
**ChNar_2**	-955.57563	668.7318	710.8368	561.3916	25.4	26.0
**ChNar_2’**	-955.575196	668.7834	710.8701	561.4592	26.6	27.2
**ChNar_3a**	-955.572168	667.7885	710.3775	560.004	34.0	33.7
**ChNar_3**	-955.5718	667.6632	710.2936	559.8502	34.9	34.5
**ChNar_3a’**	-955.571795	667.9592	710.5141	560.1989	35.1	34.9
**ChNar_3’**	-955.57139	667.8956	710.4674	560.1229	36.2	35.9
**ChNar_4**	-955.505254	660.3344	703.1491	551.6751	202.5	201.0
**ChNar_5**	-955.505744	662.7478	705.6293	554.0401	203.6	202.1
**ChNar_C1**	-955.578577	666.5119	707.6753	559.3643	14.5	16.3
**ChNar_C1’**	-955.577865	666.2917	707.5148	559.1038	16.2	17.9
**ChNar_C1a**	-955.577719	666.379	707.7086	559.1092	16.8	18.3
**ChNar_C1a’**	-955.577058	666.1162	707.5132	558.8007	18.3	19.7
**ChNar_C2a**	-955.574115	665.3562	706.6709	558.0845	25.2	26.7
**ChNar_C2a’**	-955.573425	665.1502	706.5251	557.8366	26.9	28.3
**ChNar_C2**	-955.56727	663.3942	705.3817	555.5507	41.9	42.1
**ChNar_C2’**	-955.566533	663.2296	705.2703	555.3487	43.7	43.9
**ChNar_C3**	-955.561274	663.6272	706.3694	557.2032	58.6	59.5
**Ts ChNar 1_1’**	-955.574541	665.0331	707.8777	556.3698	25.3	23.9
**Ts ChNar 1_1a**	-955.575656	665.4486	708.1306	556.9771	22.6	21.5

Table S8. Positions of the IRMPD bands of protonated naringenin chalcone [ChNar+H]$^+$ and computed vibrational modes for the lowest-energy **ChNar_1**

Wavenumber (cm^{-1})		Vibrational mode[b]
IRMPD	Calculated[a]	
836	829 (57)	βH oop (B ring)
953	990 (24)	βH oop (Cα and Cβ)
1036	1028 (108)	σ CC (A ring) + σ C-OH(c2’)
1070	1059 (52)	σ CC (Cα , Cγ, C1’ C6’ and C5’) + σ COH +
1160	1107 (54)	βH ip(B ring)
	1149 (464)	βH (O2’ , O4’ and C3’)
	1155 (709)	βOH (C4) + βH ip (C2 and C3)
	1160 (108)	βH ip (Aring)
	1165 (326)	βHip (B ring)
	1187 (190)	βH ip (O4’, O6’ and C5’)
1233	1221 (396)	σ C1- Cβ + βCH ip (B ring)
	1236 (416)	βCH ip (Cα and Cβ)+ σ C1’-Cγ
	1251 (265)	σ C2’-OH + βCH ip (A ring) + σ CC (A ring)
1293	1283 (317)	βCH ip (Cβ) + σ Cα-Cβ
	1299 (271)	σ C4-OH
1323	1318 (266)	βCH ip (B ring)
	1331 (27)	βOH ip (Cγ) + β ip CH (Cα)
1340	1346 (230)	βOH ip (C2’) + βOH ip + σ CC+ σ CγO
	1359 (51)	σ CC (B ring) + βOH ip (C4 and Cγ)
1380	1373 (61)	βCH ip (Cα) + βOH ip (C6’) + σ CC (A ring)
	1381 (348)	σ C6’O + σ CγO + βCH ip (Cβ)
1450	1434 (297)	βOH ip (Cγ) + σ CC
	1450 (63)	σ CC (B ring) + βH ip (B ring)
1530	1506 (186)	σ CC (A ring) + βH ip (A ring) + σ C4’OH
	1527 (656)	β OH ip (Cγ)
1550	1554 (837)	σ CC (B ring)
	1569 (2159)	σ Cα-Cβ
	1580 (126)	σ CC (A ring)
1616	1614 (49)	σ CC (B ring)
	1628 (467)	σ CC (A ring)

[a] Vibrational frequencies calculated at the B3LYP/6-311++G(d,p) level of theory are scaled by a factor of 0.975. The computed intensities (km mol^{-1}) are given in parenthesis. Bands with intensity lower than 20 km mol^{-1} are not reported.
[b] β = bending; σ = stretching; oop = out-of-plane; ip= in-plane; the numbering of the atoms follows Figure 1

Table S9: Extrapolated and calculated CCS of protonated species

Compound	CCS (Å^2) exp.	Optimized geometry	CCS (Å^2) calculated
$[Nar+H]^+$	160.9	**Nar_1**	160.8
		**Nar_1Ax**	162.4
$[ChNar+H]^+$	160.9	**ChNar_1**	160.1
		**ChNar_1_sT**	158.3

XYZ coordinates of optimized geometries of the most stable conformer of each family of rotamers/isomers.

Cartesian coordinates of all the other structures mentioned in this paper are available from the authors upon request.

Nar_1

H	-1.648741	-2.597665	1.183791
C	-2.199992	-1.726251	0.852092
C	-3.638140	0.518493	-0.046952
C	-1.500324	-0.642016	0.345634
C	-3.594800	-1.681034	0.897029
C	-4.324429	-0.558021	0.436547
C	-2.199367	0.539458	-0.100708
H	-5.406390	-0.581921	0.482404
O	-4.248424	1.647849	-0.496716
H	-5.210196	1.609070	-0.411803
C	-1.444121	1.664057	-0.435794
C	0.044556	1.613109	-0.353633
H	0.472857	2.293527	-1.092716
H	0.341387	1.991553	0.633133
O	-0.175187	-0.726290	0.314781
O	-1.933972	2.819388	-0.793012
H	-2.913464	2.802392	-0.802855
O	-4.335267	-2.689014	1.356247
H	-3.793583	-3.429165	1.661210
C	0.563892	0.195258	-0.577106
H	0.317063	-0.123688	-1.595066
C	2.030522	0.012707	-0.338176
C	4.782828	-0.330028	0.062261
C	2.620673	0.337909	0.892069
C	2.840682	-0.493199	-1.356897
C	4.208497	-0.660845	-1.167442
C	3.979175	0.166817	1.096972
H	2.014639	0.711387	1.710657
H	2.405787	-0.760604	-2.314444
H	4.824356	-1.051736	-1.970876
H	4.440017	0.408918	2.046500
O	6.104889	-0.466231	0.324583
H	6.574580	-0.814184	-0.441711

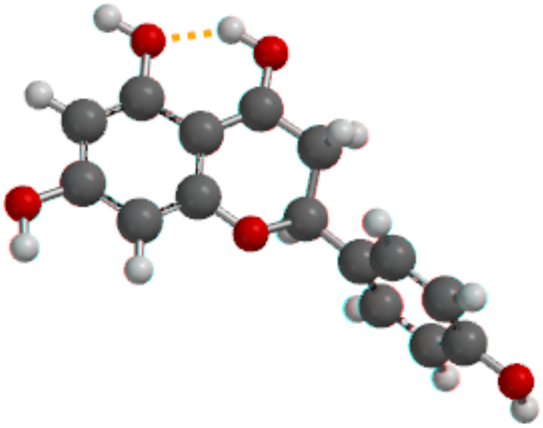

Nar_1'_Ax

H	0.251087	0.396155	-3.352348
C	-0.289461	0.932451	-2.582220
C	-1.717293	2.292192	-0.577211

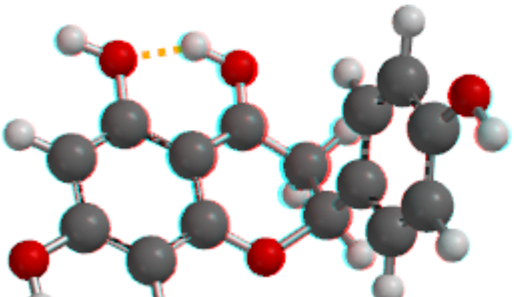

C	0.259019	1.019704	-1.312167
C	-1.529998	1.520102	-2.834578
C	-2.259094	2.196021	-1.826500
C	-0.429590	1.731081	-0.263587
H	-3.224835	2.619560	-2.073538
O	-2.335933	2.928839	0.454434
H	-3.183231	3.316209	0.197743
C	0.231269	1.913982	0.950187
C	1.617465	1.393411	1.131870
O	1.444456	0.457059	-1.115258
O	-0.241502	2.577994	1.970053
H	-1.133150	2.935060	1.775082
O	-2.121295	1.473161	-4.028408
H	-1.586366	1.002070	-4.681228
C	1.882127	0.162600	0.273897
H	2.956279	0.043875	0.139559
C	1.276678	-1.138109	0.738922
C	0.232467	-3.629470	1.514655
C	1.642668	-2.312670	0.067410
C	0.385543	-1.242482	1.815267
C	-0.132481	-2.470761	2.203713
C	1.127290	-3.545121	0.440664
H	2.341089	-2.263361	-0.761298
H	0.096590	-0.371508	2.392538
H	-0.810036	-2.552414	3.044379
H	1.428174	-4.442479	-0.090387
O	-0.305990	-4.797424	1.939480
H	0.024295	-5.540912	1.422310
H	2.299442	2.202641	0.838974
H	1.804315	1.192546	2.187592

Nar_C1a

C	-1.504240	-0.526494	0.235538
C	-2.214731	0.598626	-0.146110
C	-1.508723	1.813122	-0.560651
C	-0.005693	1.768835	-0.438715
H	0.435772	2.478059	-1.141198
H	0.252590	2.115143	0.569018
O	-0.214961	-0.647531	0.125907
O	-2.120291	2.815751	-0.919212
C	0.557022	0.375642	-0.702433
H	0.332056	0.069977	-1.727504
C	2.008659	0.181874	-0.422093
C	4.743961	-0.202562	0.050736

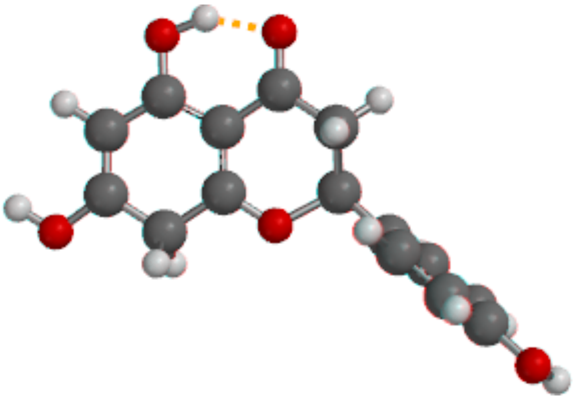

C	2.586161	0.569440	0.796982
C	2.824778	-0.406164	-1.392792
C	4.183324	-0.594050	-1.168355
C	3.935122	0.377212	1.037684
H	1.980331	1.016518	1.577585
H	2.400772	-0.716783	-2.342164
H	4.803709	-1.044281	-1.936213
H	4.386333	0.669691	1.977474
O	6.055558	-0.352979	0.346205
H	6.534104	-0.751757	-0.389555
C	-3.646143	0.593554	-0.027679
C	-4.363334	-0.516022	0.487210
H	-5.442739	-0.443930	0.549188
C	-3.677160	-1.616732	0.903599
C	-2.187557	-1.724730	0.815277
H	-1.777482	-1.925674	1.813395
H	-1.924740	-2.607968	0.219850
O	-4.224929	-2.705660	1.421909
H	-5.189406	-2.643488	1.484564
O	-4.328597	1.637011	-0.390310
H	-3.689527	2.346348	-0.707135

Nar_C2

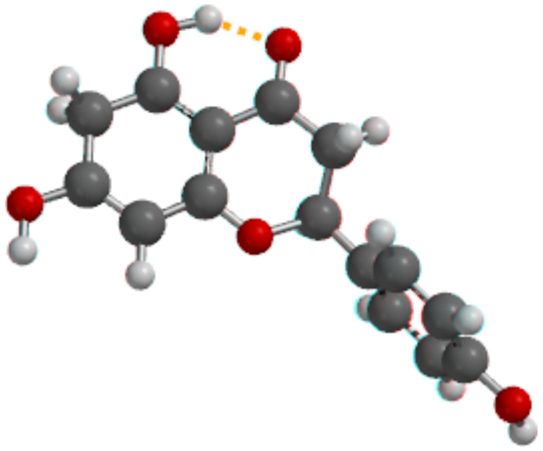

C	-1.480384	-0.578761	0.272719
C	-2.195611	0.583145	-0.145837
C	-1.483742	1.794237	-0.555016
C	0.016543	1.751858	-0.431045
H	0.460148	2.458149	-1.135211
H	0.276967	2.098213	0.576144
O	-0.185617	-0.657966	0.158297
O	-2.099409	2.793483	-0.922051
C	0.573829	0.354434	-0.686441
H	0.346496	0.041910	-1.708597
C	2.028225	0.165196	-0.412291
C	4.768188	-0.202079	0.045823
C	2.607481	0.546349	0.807674
C	2.844417	-0.408116	-1.391336
C	4.205452	-0.587575	-1.174020
C	3.959055	0.362314	1.041241
H	2.000789	0.980675	1.594809
H	2.418681	-0.714428	-2.341331
H	4.826078	-1.026676	-1.948142
H	4.411912	0.649683	1.981840
O	6.082496	-0.344884	0.334339

H	6.560724	-0.732868	-0.407355
C	-3.583790	0.593500	-0.048517
C	-3.490984	-1.714595	0.941622
O	-4.213329	-2.714034	1.426633
H	-3.668828	-3.458878	1.720658
O	-4.296879	1.617584	-0.398421
H	-3.665696	2.337626	-0.718081
C	-4.343240	-0.572562	0.488744
H	-5.050641	-0.926077	-0.273401
H	-4.974102	-0.232486	1.320092
C	-2.132272	-1.707703	0.838817
H	-1.522956	-2.548669	1.147639

Nar_2a

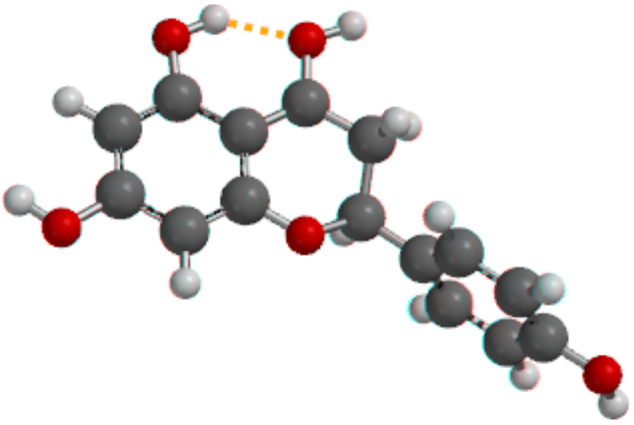

H	-1.648741	-2.597665	1.183791
C	-2.199992	-1.726251	0.852092
C	-3.638140	0.518493	-0.046952
C	-1.500324	-0.642016	0.345634
C	-3.594800	-1.681034	0.897029
C	-4.324429	-0.558021	0.436547
C	-2.199367	0.539458	-0.100708
H	-5.406390	-0.581921	0.482404
O	-4.248424	1.647849	-0.496716
H	-5.210196	1.609070	-0.411803
C	-1.444121	1.664057	-0.435794
C	0.044556	1.613109	-0.353633
H	0.472857	2.293527	-1.092716
H	0.341387	1.991553	0.633133
O	-0.175187	-0.726290	0.314781
O	-1.933972	2.819388	-0.793012
H	-2.913464	2.802392	-0.802855
O	-4.335267	-2.689014	1.356247
H	-3.793583	-3.429165	1.661210
C	0.563892	0.195258	-0.577106
H	0.317063	-0.123688	-1.595066
C	2.030522	0.012707	-0.338176
C	4.782828	-0.330028	0.062261
C	2.620673	0.337909	0.892069
C	2.840682	-0.493199	-1.356897
C	4.208497	-0.660845	-1.167442
C	3.979175	0.166817	1.096972
H	2.014639	0.711387	1.710657
H	2.405787	-0.760604	-2.314444
H	4.824356	-1.051736	-1.970876

H	4.440017	0.408918	2.046500
O	6.104889	-0.466231	0.324583
H	6.574580	-0.814184	-0.441711

Nar_3

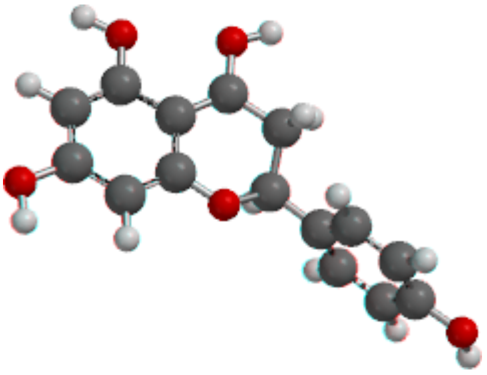

H	-1.648741	-2.597665	1.183791
C	-2.199992	-1.726251	0.852092
C	-3.638140	0.518493	-0.046952
C	-1.500324	-0.642016	0.345634
C	-3.594800	-1.681034	0.897029
C	-4.324429	-0.558021	0.436547
C	-2.199367	0.539458	-0.100708
H	-5.406390	-0.581921	0.482404
O	-4.248424	1.647849	-0.496716
H	-5.210196	1.609070	-0.411803
C	-1.444121	1.664057	-0.435794
C	0.044556	1.613109	-0.353633
H	0.472857	2.293527	-1.092716
H	0.341387	1.991553	0.633133
O	-0.175187	-0.726290	0.314781
O	-1.933972	2.819388	-0.793012
H	-2.913464	2.802392	-0.802855
O	-4.335267	-2.689014	1.356247
H	-3.793583	-3.429165	1.661210
C	0.563892	0.195258	-0.577106
H	0.317063	-0.123688	-1.595066
C	2.030522	0.012707	-0.338176
C	4.782828	-0.330028	0.062261
C	2.620673	0.337909	0.892069
C	2.840682	-0.493199	-1.356897
C	4.208497	-0.660845	-1.167442
C	3.979175	0.166817	1.096972
H	2.014639	0.711387	1.710657
H	2.405787	-0.760604	-2.314444
H	4.824356	-1.051736	-1.970876
H	4.440017	0.408918	2.046500
O	6.104889	-0.466231	0.324583
H	6.574580	-0.814184	-0.441711

Nar_4

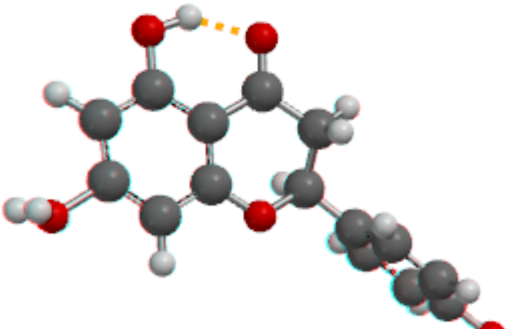

H	-1.653611	-2.490867	1.076289
C	-2.167607	-1.602986	0.736928
C	-3.565492	0.690436	-0.199483
C	-1.456464	-0.493264	0.237824

C	-3.531602	-1.468163	0.724949
C	-4.294236	-0.411294	0.288913
C	-2.145332	0.653772	-0.214932
H	-5.375533	-0.378418	0.316948
O	-4.246002	1.745378	-0.614431
H	-3.595495	2.439907	-0.897629
C	-1.383672	1.850029	-0.608237
C	0.112337	1.766045	-0.432860
H	0.595170	2.455523	-1.127265
H	0.350268	2.107691	0.581785
O	-0.128122	-0.589953	0.226721
O	-1.947552	2.862516	-1.011771
C	0.622936	0.343097	-0.652999
H	0.384015	0.025766	-1.673680
C	2.086310	0.137998	-0.402533
C	4.832402	-0.248577	0.011350
C	2.676352	0.474880	0.820210
C	2.895234	-0.400616	-1.409688
C	4.255993	-0.589711	-1.214718
C	4.035155	0.281106	1.032671
H	2.076112	0.880468	1.627365
H	2.457951	-0.673188	-2.364792
H	4.884513	-1.000574	-1.994933
H	4.477948	0.541789	1.988677
O	6.166080	-0.457052	0.148590
H	6.467576	-0.187718	1.023138
O	-4.273384	-2.673856	1.257516
H	-4.886581	-3.073920	0.612572
H	-4.725666	-2.516244	2.107503

Nar_5

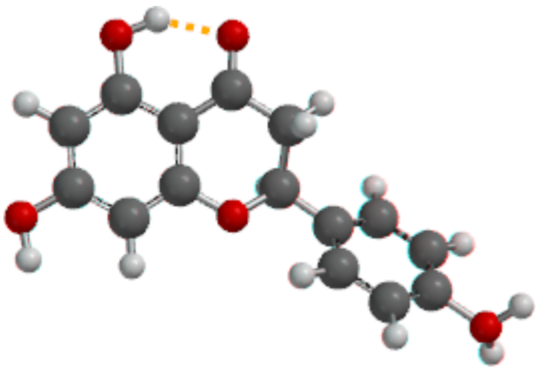

H	-2.034495	-2.286126	1.596410
C	-2.564120	-1.469790	1.120112
C	-3.940011	0.627446	-0.160016
C	-1.862249	-0.482977	0.460450
C	-3.969720	-1.393333	1.137260
C	-4.656573	-0.363704	0.500700
C	-2.511432	0.591822	-0.189672
H	-5.737346	-0.330072	0.523908
O	-4.611308	1.615475	-0.752691
H	-3.960811	2.243786	-1.142172
C	-1.752968	1.658680	-0.797253
C	-0.238511	1.556025	-0.680364
H	0.210605	2.056401	-1.540455

H	0.073987	2.093357	0.222701
O	-0.488737	-0.571874	0.479379
O	-2.263222	2.628327	-1.363066
O	-4.718870	-2.327548	1.763863
H	-4.160882	-2.996037	2.176402
C	0.192583	0.087865	-0.589684
H	-0.070575	-0.420956	-1.527324
C	1.680275	-0.059927	-0.355639
C	4.365905	-0.239634	0.037555
C	2.183807	-0.386700	0.907667
C	2.564616	0.165185	-1.418881
C	3.942977	0.079459	-1.236486
C	3.558950	-0.482370	1.124901
H	1.497228	-0.574957	1.721835
H	2.183237	0.405396	-2.404850
H	4.629418	0.250567	-2.057539
H	3.967755	-0.734255	2.095236
O	5.846515	-0.348770	0.297786
H	6.345095	0.485296	0.218000
H	6.298879	-1.076060	-0.168072

Nar_6

H	-1.653611	-2.490867	1.076289
C	-2.167607	-1.602986	0.736928
C	-3.565492	0.690436	-0.199483
C	-1.456464	-0.493264	0.237824
C	-3.531602	-1.468163	0.724949
C	-4.294236	-0.411294	0.288913
C	-2.145332	0.653772	-0.214932
H	-5.375533	-0.378418	0.316948
O	-4.246002	1.745378	-0.614431
H	-3.595495	2.439907	-0.897629
C	-1.383672	1.850029	-0.608237
C	0.112337	1.766045	-0.432860
H	0.595170	2.455523	-1.127265
H	0.350268	2.107691	0.581785
O	-0.128122	-0.589953	0.226721
O	-1.947552	2.862516	-1.011771
C	0.622936	0.343097	-0.652999
H	0.384015	0.025766	-1.673680
C	2.086310	0.137998	-0.402533
C	4.832402	-0.248577	0.011350
C	2.676352	0.474880	0.820210
C	2.895234	-0.400616	-1.409688

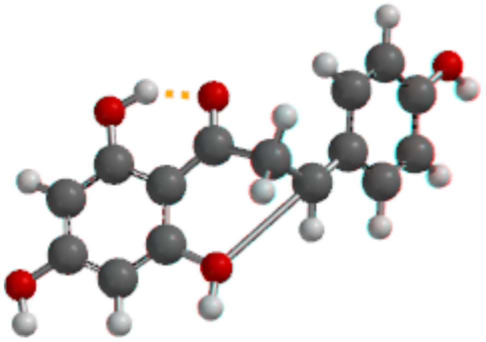

C	4.255993	-0.589711	-1.214718
C	4.035155	0.281106	1.032671
H	2.076112	0.880468	1.627365
H	2.457951	-0.673188	-2.364792
H	4.884513	-1.000574	-1.994933
H	4.477948	0.541789	1.988677
O	6.166080	-0.457052	0.148590
H	6.467576	-0.187718	1.023138
O	-4.273384	-2.673856	1.257516
H	-4.886581	-3.073920	0.612572
H	-4.725666	-2.516244	2.107503

Ts Nar 1-1’

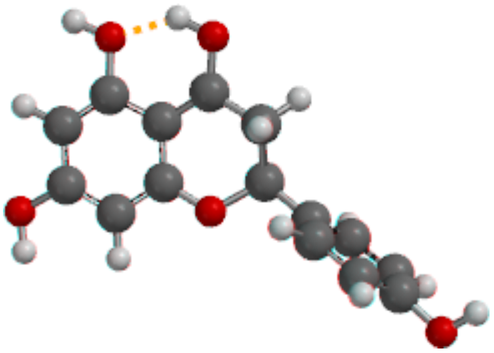

H	-1.637612	-2.616933	1.192971
C	-2.192570	-1.751012	0.853317
C	-3.641008	0.478560	-0.067522
C	-1.498781	-0.670615	0.332662
C	-3.587858	-1.710058	0.901027
C	-4.322282	-0.594890	0.429484
C	-2.202288	0.503740	-0.124778
H	-5.404026	-0.622201	0.477851
O	-4.255686	1.600716	-0.528531
H	-5.217511	1.559592	-0.443378
C	-1.451688	1.627467	-0.472225
C	0.037672	1.581332	-0.394167
H	0.461741	2.252522	-1.144040
H	0.338258	1.972649	0.586403
O	-0.172617	-0.750973	0.299369
O	-1.945431	2.778447	-0.836867
H	-2.925021	2.759184	-0.844048
O	-4.323501	-2.714943	1.373203
H	-3.779506	-3.450560	1.685522
C	0.558070	0.161391	-0.600581
H	0.315819	-0.168743	-1.615957
C	2.028730	-0.011314	-0.355239
C	4.775660	-0.333595	0.054796
C	2.597100	0.276943	0.891361
C	2.850126	-0.472516	-1.386995
C	4.218365	-0.632629	-1.187283
C	3.960077	0.109020	1.099812
H	1.975765	0.607671	1.716812
H	2.422514	-0.716309	-2.354072
H	4.858010	-1.002157	-1.979627
H	4.404797	0.305525	2.067915

O	6.113097	-0.568259	0.284073
H	6.641585	0.212947	0.088730

Ts Nar 1-1a

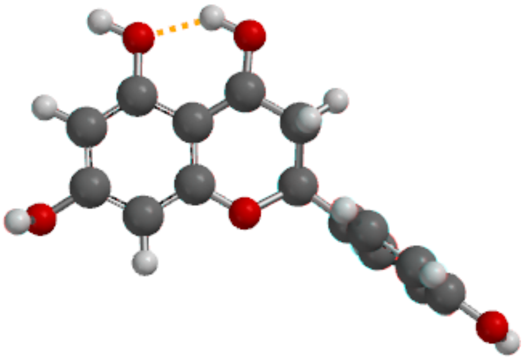

H	-1.652465	-2.619772	1.177277
C	-2.183469	-1.735910	0.850759
C	-3.615410	0.509126	-0.058671
C	-1.477971	-0.650754	0.340653
C	-3.571000	-1.687599	0.898298
C	-4.300775	-0.571128	0.429601
C	-2.179136	0.526089	-0.107762
H	-5.383659	-0.600424	0.451307
O	-4.225191	1.634987	-0.520739
H	-5.188013	1.592866	-0.449142
C	-1.421428	1.652411	-0.451502
C	0.065959	1.601496	-0.363502
H	0.498298	2.285242	-1.097011
H	0.355758	1.976019	0.627145
O	-0.154238	-0.737595	0.305207
O	-1.913683	2.800356	-0.817128
H	-2.894225	2.780263	-0.832147
O	-4.260785	-2.786214	1.299824
H	-4.482456	-2.772673	2.239350
C	0.584726	0.183195	-0.588271
H	0.333990	-0.136227	-1.605296
C	2.051464	-0.000922	-0.355051
C	4.802965	-0.358386	0.035597
C	2.652051	0.345883	0.864281
C	2.850867	-0.535957	-1.367647
C	4.218109	-0.711191	-1.183054
C	4.010232	0.168022	1.064254
H	2.054946	0.740939	1.679202
H	2.407390	-0.821631	-2.315869
H	4.825228	-1.125583	-1.981256
H	4.479243	0.426529	2.005365
O	6.125175	-0.499689	0.292785
H	6.587501	-0.871769	-0.466862

ChNar_1

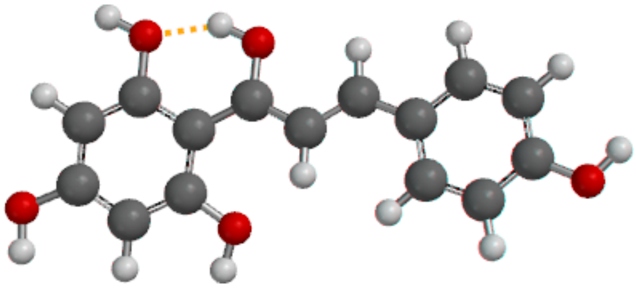

C	0.442541	3.564220	-0.792325
C	0.343183	3.602851	1.974926
C	0.181860	2.319394	-0.128512
C	0.660511	4.750842	-0.129469

C	0.613329	4.773832	1.267007
C	0.129041	2.408248	1.300706
H	0.866618	5.668117	-0.668149
H	0.288686	3.621178	3.058975
O	0.832184	5.957606	1.857042
H	0.782953	5.895291	2.818658
O	-0.160757	1.295131	2.010379
H	-0.199794	1.496067	2.953077
C	-0.025538	1.083851	-0.870211
C	-0.063914	-0.213464	-0.306061
H	0.093187	-0.305219	0.752156
C	-0.283699	-1.327798	-1.084081
H	-0.451630	-1.171596	-2.145052
C	-0.322548	-2.686492	-0.647782
C	-0.435880	-5.395788	0.086936
C	-0.564976	-3.697329	-1.608474
C	-0.135878	-3.086524	0.701013
C	-0.191574	-4.410960	1.064149
C	-0.622158	-5.030676	-1.255210
H	-0.709796	-3.415420	-2.645581
H	-0.051763	-4.724756	2.091001
H	-0.809219	-5.791064	-2.005987
O	-0.477379	-6.668281	0.513344
H	-0.650533	-7.280302	-0.212681
H	0.052938	-2.344471	1.466728
O	-0.177294	1.120626	-2.186556
H	-0.072392	2.023954	-2.540875
O	0.486188	3.545055	-2.159481
H	0.633504	4.423876	-2.529608

ChNar_1a_sT

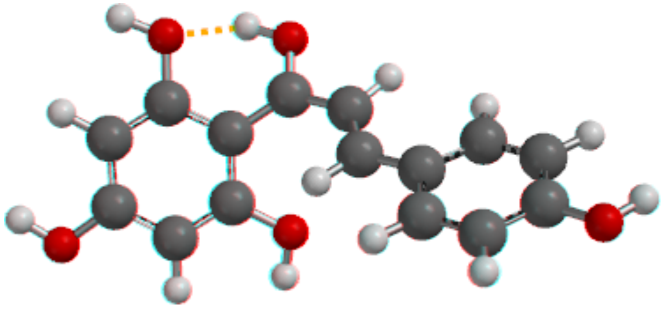

C	-0.325390	2.996463	-1.725518
C	-1.254877	3.208320	0.883261
C	-0.730755	1.805187	-1.060982
C	-0.317690	4.233323	-1.106714
C	-0.782384	4.335642	0.209793
C	-1.236686	1.969137	0.264012
H	0.027995	5.110902	-1.642948
H	-1.648660	3.325516	1.886035
O	-0.820092	5.497166	0.882780
H	-0.486464	6.231711	0.353632
O	-1.747072	0.882023	0.887457
H	-2.175424	1.139583	1.712699

C	-0.717833	0.522007	-1.752054
C	-0.432293	-0.742548	-1.206790
O	-0.994961	0.469367	-3.047988
H	-1.042423	1.354946	-3.443683
O	0.068097	2.859279	-3.025032
H	0.360716	3.698677	-3.399585
H	-0.667641	-1.571982	-1.863451
C	0.241911	-0.947184	-0.027255
H	0.552817	-0.071398	0.532693
C	0.636385	-2.196166	0.536425
C	1.480067	-4.591679	1.735945
C	1.438259	-2.191534	1.706414
C	0.268763	-3.451167	-0.009044
C	0.679048	-4.627515	0.577645
C	1.857894	-3.361612	2.298249
H	1.731898	-1.242006	2.140422
H	-0.349813	-3.500472	-0.896336
H	0.386570	-5.582052	0.152666
H	2.472435	-3.360941	3.189387
O	1.914671	-5.700441	2.353018
H	1.612932	-6.500555	1.904846

ChNar_2a

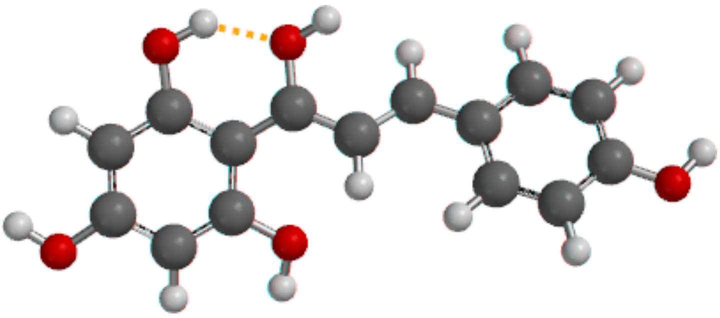

C	0.606601	3.633703	-0.801667
C	0.134558	3.640460	1.968108
C	0.228532	2.387992	-0.156149
C	0.775146	4.800455	-0.072548
C	0.545280	4.806053	1.300227
C	-0.024068	2.471097	1.265873
H	1.076115	5.695299	-0.603444
H	-0.066082	3.689754	3.031711
O	0.682262	5.900341	2.057207
H	0.964855	6.661949	1.534833
O	0.854400	3.749272	-2.109010
H	0.643303	2.927905	-2.576954
O	-0.454599	1.350951	1.891403
H	-0.642352	1.539647	2.819275
C	0.129098	1.164128	-0.870293
C	0.086645	-0.144324	-0.325136
H	0.352480	-0.239494	0.713751
C	-0.316826	-1.249950	-1.033230
H	-0.720562	-1.112885	-2.036279
C	-0.338130	-2.606757	-0.581651
C	-0.446667	-5.298975	0.214324

C	-0.908999	-3.595070	-1.418032
C	0.183679	-3.019583	0.671296
C	0.133158	-4.335931	1.063711
C	-0.968021	-4.919207	-1.032487
H	-1.315669	-3.307323	-2.381501
H	0.533954	-4.659337	2.016119
H	-1.411913	-5.662176	-1.686502
O	-0.463086	-6.563466	0.661684
H	-0.873225	-7.161702	0.024618
H	0.639357	-2.295335	1.334966
O	0.098425	1.295490	-2.220494
H	0.282347	0.457018	-2.663733

ChNar_3a

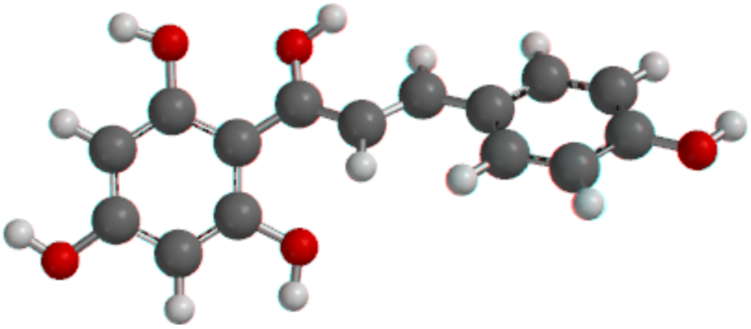

C	0.744500	3.536422	-0.765851
C	-0.014580	3.605258	1.932844
C	0.200127	2.347249	-0.172162
C	0.902723	4.702455	-0.031621
C	0.517613	4.738721	1.311223
C	-0.162618	2.437262	1.212754
H	1.336756	5.579551	-0.500806
H	-0.316232	3.668657	2.971513
O	0.634370	5.836590	2.072589
H	1.002786	6.579561	1.578997
O	1.156509	3.472848	-2.044454
H	1.561339	4.306819	-2.311564
O	-0.705353	1.340092	1.789359
H	-0.970676	1.536593	2.696174
C	0.009042	1.140781	-0.926472
C	0.043763	-0.165816	-0.373629
H	0.435989	-0.251776	0.627088
C	-0.427749	-1.277255	-1.026143
H	-0.945825	-1.140901	-1.975308
C	-0.378167	-2.635331	-0.580863
C	-0.353165	-5.331293	0.208293
C	-1.038505	-3.627207	-1.343159
C	0.304433	-3.046379	0.592466
C	0.319827	-4.364987	0.981004
C	-1.033910	-4.953338	-0.959328
H	-1.565613	-3.340508	-2.246759
H	0.843303	-4.686718	1.872458
H	-1.549961	-5.698960	-1.554726
O	-0.298344	-6.598326	0.647499
H	-0.785153	-7.197324	0.067747

H	0.834863	-2.319774	1.195326
O	-0.198327	1.316991	-2.226633
H	-0.103764	0.490039	-2.717854

ChNar_4

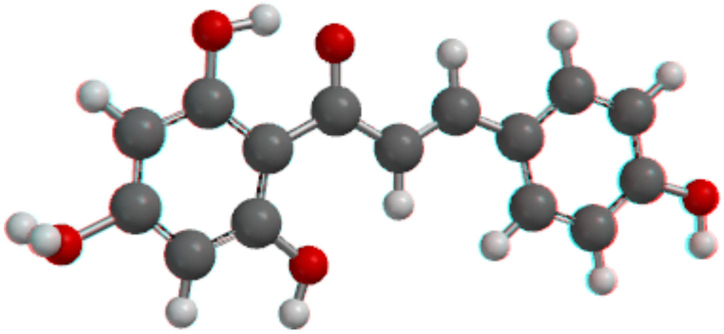

C	0.174334	3.446494	-0.955796
C	0.392483	3.557859	1.856068
C	0.093516	2.227843	-0.200937
C	0.373940	4.696118	-0.319694
C	0.470266	4.658782	1.038520
C	0.196935	2.325612	1.206828
H	0.435806	5.590864	-0.925291
H	0.473607	3.632755	2.932312
O	0.071785	3.479042	-2.262984
H	-0.081460	2.501832	-2.545071
O	0.097661	1.207574	1.958885
H	0.189298	1.409090	2.897084
C	-0.091755	0.955781	-0.992209
O	-0.226068	1.082780	-2.232621
C	-0.099376	-0.358798	-0.388992
H	0.026402	-0.447934	0.675170
C	-0.256618	-1.457031	-1.181052
H	-0.372138	-1.274012	-2.245901
C	-0.288649	-2.840014	-0.780134
C	-0.366034	-5.574084	-0.128335
C	-0.429213	-3.830259	-1.778160
C	-0.189769	-3.268746	0.560675
C	-0.228685	-4.611010	0.885460
C	-0.466884	-5.175655	-1.466352
H	-0.508032	-3.522954	-2.815401
H	-0.154720	-4.925925	1.921783
H	-0.572639	-5.932535	-2.233282
O	-0.408752	-6.900060	0.126046
H	-0.329393	-7.073736	1.070999
H	-0.086529	-2.542558	1.357994
O	0.684939	5.967691	1.765211
H	-0.054550	6.595344	1.658825
H	1.530293	6.399848	1.540352

ChNar_5

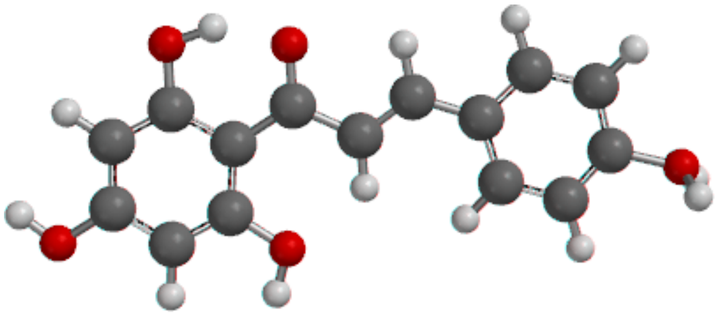

C	0.243688	3.879489	-0.940894
C	0.562125	3.933390	1.833357
C	0.229591	2.627064	-0.221858
C	0.404016	5.100996	-0.286307

C	0.563593	5.123504	1.089330
C	0.400239	2.723061	1.194202
H	0.403720	6.006651	-0.880843
H	0.692744	3.987966	2.908198
O	0.729793	6.263607	1.792848
H	0.721590	7.029508	1.206598
O	0.103217	3.928686	-2.260370
H	0.008055	2.990722	-2.581978
O	0.407211	1.576487	1.943513
H	0.536958	1.811065	2.869370
C	0.047041	1.404551	-0.995206
O	-0.068415	1.445821	-2.237568
C	-0.022545	0.051255	-0.363078
H	0.075181	-0.017145	0.707935
C	-0.200274	-1.026141	-1.144540
H	-0.278546	-0.857692	-2.214228
C	-0.292223	-2.416991	-0.702549
C	-0.465346	-5.058269	0.002134
C	-0.401468	-3.422569	-1.682135
C	-0.278609	-2.801519	0.654180
C	-0.365504	-4.137094	1.027497
C	-0.489164	-4.770159	-1.343294
H	-0.415640	-3.144242	-2.729042
H	-0.356515	-4.427046	2.072037
H	-0.569882	-5.544897	-2.095041
H	-0.204608	-2.052935	1.432121
O	-0.563338	-6.519488	0.353599
H	-1.381837	-6.786313	0.811090
H	0.225148	-6.901324	0.780922

ChNar_C1

C	0.050709	3.538527	-0.896854
C	-0.012115	2.287973	-0.139887
O	0.151219	3.569980	-2.128004
H	0.179822	2.084706	-2.465087
C	0.063867	1.033078	-0.884192
C	0.039885	-0.273764	-0.331119
H	-0.031281	-0.375112	0.735934
C	0.099821	-1.381318	-1.143887
H	0.158236	-1.206159	-2.213729
C	0.090318	-2.752467	-0.742618
C	0.074664	-5.482852	-0.082118
C	0.130425	-3.749238	-1.745816
C	0.044030	-3.176585	0.609886

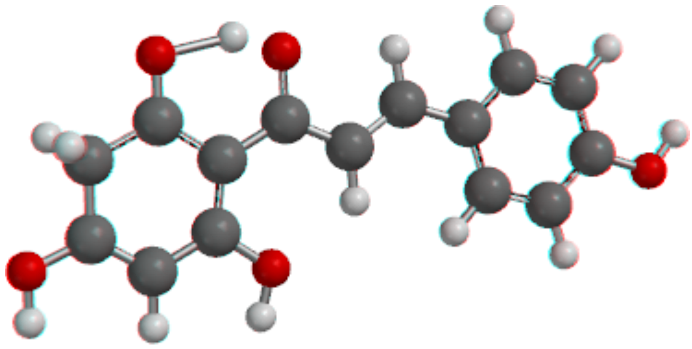

C	0.037249	-4.511738	0.937264
C	0.121592	-5.093443	-1.428847
H	0.167523	-3.448147	-2.787054
H	0.004460	-4.844164	1.967237
H	0.151019	-5.843308	-2.212408
O	0.064090	-6.767298	0.310985
H	0.092410	-7.369776	-0.442662
H	0.017473	-2.444905	1.408071
O	0.168293	1.112013	-2.188635
C	-0.001648	4.875644	-0.186877
H	0.910770	5.428530	-0.441649
H	-0.819046	5.460725	-0.624220
C	-0.154177	2.404886	1.255443
C	-0.225376	3.648684	1.953971
H	-0.339175	3.627615	3.033751
C	-0.154829	4.830815	1.287285
O	-0.232170	1.296111	1.992948
H	-0.328816	1.499167	2.932775
O	-0.210713	6.031811	1.849093
H	-0.308528	5.990008	2.811021

ChNar_C2a

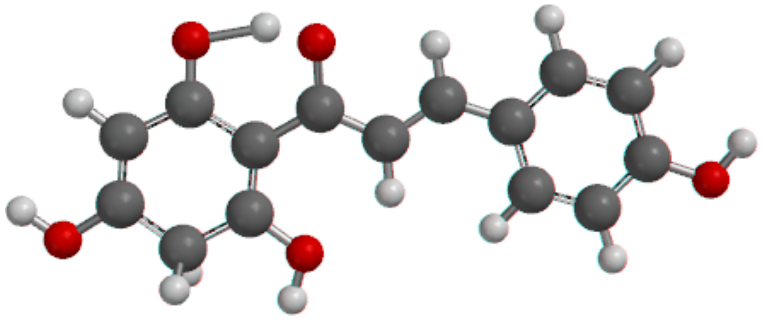

C	0.218496	2.288962	-0.157528
C	0.324475	2.383523	1.207053
O	0.249385	1.304910	1.991128
H	0.343412	1.531061	2.926288
C	0.025536	1.035920	-0.913272
C	-0.063531	-0.258846	-0.348499
H	0.013320	-0.358775	0.718712
C	-0.240409	-1.361436	-1.156127
H	-0.303986	-1.180734	-2.224973
C	-0.349756	-2.724346	-0.753597
C	-0.573385	-5.444216	-0.091189
C	-0.511158	-3.712114	-1.755521
C	-0.305651	-3.152795	0.598841
C	-0.415691	-4.482407	0.926879
C	-0.620898	-5.050498	-1.437616
H	-0.547844	-3.406577	-2.795447
H	-0.384730	-4.818953	1.955539
H	-0.742005	-5.793460	-2.218735
O	-0.673036	-6.722872	0.302119
H	-0.781330	-7.319360	-0.449322
H	-0.186474	-2.427837	1.394471

O	-0.067450	1.133238	-2.209591
C	0.307671	3.562623	-0.937659
C	0.511978	4.825116	-0.265668
H	0.573881	5.700244	-0.901855
C	0.620114	4.892762	1.073446
C	0.524916	3.678079	1.934603
H	1.437068	3.607287	2.543540
H	-0.302509	3.818196	2.644321
O	0.207669	3.544147	-2.176936
H	0.021091	2.125485	-2.470873
O	0.813868	5.997081	1.801310
H	0.876963	6.786590	1.246156

ChNar_C3

C	0.368354	3.654530	-0.980484
C	0.392462	3.852500	1.811707
C	0.178387	2.455954	-0.194726
C	0.566304	4.892522	-0.379743
C	0.579977	4.990049	1.002904
C	0.194935	2.621578	1.221833
H	0.707528	5.769578	-0.996266
H	0.398871	3.944164	2.893592
O	0.776835	6.212514	1.531550
H	0.770099	6.186706	2.495089
O	0.366122	3.628168	-2.309748
H	0.213027	2.699612	-2.602242
C	-0.009013	1.200089	-0.886814
O	-0.035443	1.127653	-2.125812
C	-0.293774	-1.282245	-1.043997
H	-0.365973	-1.017256	-2.097414
C	-0.311918	-2.622591	-0.712247
C	-0.373274	-5.368382	-0.155004
C	-0.429545	-3.593961	-1.765816
C	-0.223516	-3.100333	0.638432
C	-0.255712	-4.437765	0.912574
C	-0.459598	-4.933234	-1.497798
H	-0.494609	-3.243910	-2.789981
H	-0.190656	-4.798039	1.933756
H	-0.546661	-5.676779	-2.279824
O	-0.407208	-6.675338	0.043026
H	-0.347044	-6.917549	0.978160
H	-0.133162	-2.391099	1.451993
O	0.003061	1.513319	2.001217
H	0.027815	1.765750	2.931252
C	-0.187413	-0.128585	-0.126009
H	0.614734	-0.264541	0.604770

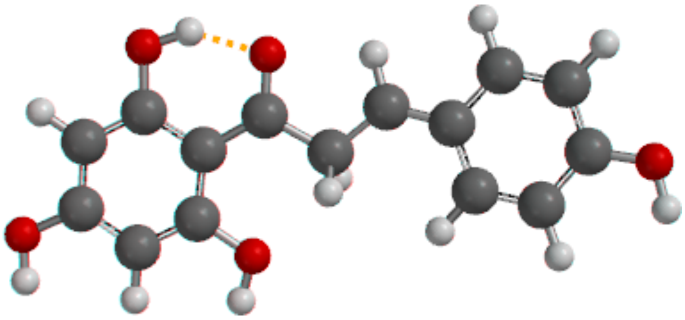

H	-1.093991	-0.063079	0.492069

Ts ChNar 1-1’

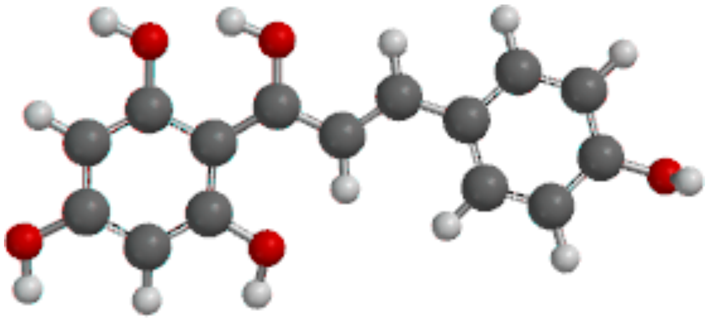

C	0.385164	3.571236	-0.814440
C	0.325091	3.603493	1.954816
C	0.167992	2.317328	-0.148156
C	0.577805	4.762232	-0.153433
C	0.550128	4.782536	1.244071
C	0.135161	2.403836	1.283410
H	0.750339	5.685518	-0.693583
H	0.287569	3.619544	3.039544
O	0.742632	5.970974	1.830909
H	0.709074	5.909515	2.793548
O	-0.108931	1.283217	1.996440
H	-0.131751	1.479952	2.940820
C	-0.018185	1.080611	-0.888679
C	-0.054816	-0.221434	-0.322483
H	0.093017	-0.312838	0.736937
C	-0.267533	-1.331764	-1.100976
H	-0.425056	-1.177818	-2.163789
C	-0.317443	-2.696556	-0.662503
C	-0.467239	-5.400707	0.066964
C	-0.563549	-3.699960	-1.627888
C	-0.150587	-3.091894	0.686083
C	-0.229859	-4.422202	1.045495
C	-0.643018	-5.034597	-1.271087
H	-0.700075	-3.413174	-2.664872
H	-0.121304	-4.732488	2.077698
H	-0.848106	-5.802755	-2.006415
O	-0.625489	-6.706390	0.443096
H	0.206847	-7.193458	0.425353
H	0.032543	-2.350819	1.454316
O	-0.154173	1.110518	-2.204336
H	-0.069916	2.015744	-2.561327
O	0.410798	3.554700	-2.181395
H	0.522868	4.437901	-2.554140

Ts ChNar 1-1a

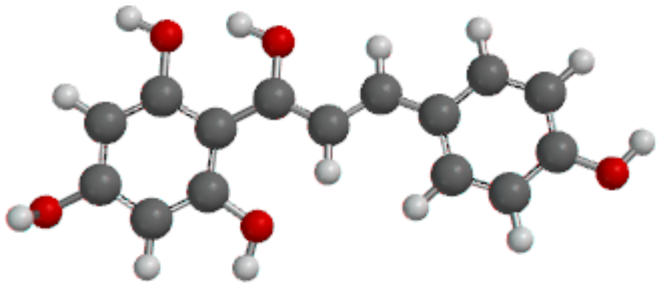

C	0.408889	3.554648	-0.781640
C	0.366104	3.598399	1.985969
C	0.196055	2.308388	-0.110354
C	0.600739	4.751520	-0.119024
C	0.586832	4.771082	1.274688
C	0.174747	2.393751	1.319240
H	0.748868	5.675289	-0.666393

H	0.323148	3.638254	3.068654
O	0.692396	5.960203	1.936439
H	1.606840	6.173598	2.158370
O	-0.061927	1.274016	2.039227
H	-0.083264	1.481988	2.981397
C	-0.012000	1.065420	-0.851432
C	-0.055909	-0.227564	-0.283739
H	0.091357	-0.317150	0.776057
C	-0.277368	-1.343431	-1.062969
H	-0.427251	-1.187526	-2.126659
C	-0.341045	-2.698462	-0.625484
C	-0.508855	-5.403949	0.110041
C	-0.575204	-3.708210	-1.590776
C	-0.191322	-3.098275	0.728763
C	-0.273965	-4.420651	1.091938
C	-0.658363	-5.039348	-1.237361
H	-0.692112	-3.425503	-2.631141
H	-0.164128	-4.735046	2.122192
H	-0.838227	-5.798770	-1.990756
O	-0.578724	-6.673629	0.537369
H	-0.744104	-7.286230	-0.190359
H	-0.012214	-2.357113	1.497569
O	-0.168047	1.102560	-2.166180
H	-0.072238	2.007805	-2.520675
O	0.426703	3.538718	-2.151078
H	0.513592	4.425218	-2.521892
